# Burden of gastroesophageal reflux disease among women of childbearing age, with projections to 2050: an analysis of the Global Burden of Disease study 2021

**DOI:** 10.3389/fgwh.2025.1673878

**Published:** 2025-09-19

**Authors:** Siyu Zhou, Yanping Wang, Nengyi Hou, Kun Hu, Shun Jiang, Junzhao You, Hongtao Tang, Jie Zeng, Minghui Pang

**Affiliations:** 1 Department of Gastrointestinal Surgery, The Affiliated Hospital, Southwest Medical University, Luzhou, China; 2 Department of Gastroenterology, Union Hospital, Tongji Medical College, Huazhong University of Science and Technology, Wuhan, China; 3 Department of Geriatric General Surgery, Sichuan Provincial People’s Hospital, School of Medicine, University of Electronic Science and Technology, Chengdu, China; 4 School of Clinical Medicine, North Sichuan Medical College, Nanchong, China

**Keywords:** Global Burden of Disease, gastroesophageal reflux disease, women of childbearing age, epidemiology, disease burden projection

## Abstract

**Background:**

Gastroesophageal reflux disease (GERD) is a common chronic digestive disorder characterized by the reflux of gastroduodenal contents into the esophagus, causing uncomfortable symptoms and potential tissue damage. It affects over 1 billion people worldwide, imposing substantial economic and health burdens. Notably, women of childbearing age face unique challenges due to hormonal fluctuations, pregnancy, and gender-specific social roles, yet systematic global analyses of GERD burden in this population remain scarce.

**Methods:**

This study evaluated the global, regional, and national burden of GERD among WCBA from 1990 to 2021 and projected trends through 2050. Data were sourced from the 2021 Global Burden of Disease (GBD) study, including incidence, prevalence, years lived with disability (YLDs), and their age-standardized rates. Temporal trends were analyzed using joinpoint regression (average annual percentage change, AAPC), and future projections were generated via Bayesian age-period-cohort models. Associations with the Socio-demographic Index (SDI) were explored.

**Results:**

Globally, the number of incident and prevalent GERD cases among WCBA increased by 64.09% and 66.44% from 1990 to 2021, reaching almost 99.1 million and 245.2 million in 2021, respectively. The AAPCs for age-standardized incidence rate (ASIR), age-standardized prevalence rate (ASPR), and age-standardized YLD rate (ASYR) were 0.24, 0.23, and 0.23, respectively. Regionally, South Asia had the highest absolute burden, while Tropical Latin America had the highest ASRs. Nationally, the Republic of India reported the highest incidence, the People's Republic of China the highest prevalence, Brazil the highest ASRs, and Norway the lowest. SDI was negatively correlated with GERD burden, with the most notable upward trends in middle SDI regions. By age, burden increased with age and peaked in the 25–29 years group. Joinpoint analysis showed accelerated growth post-2011. Projections to 2,050 forecast continued rises in incidence, prevalence, and ASRs.

**Conclusion:**

The global GERD burden among WCBA is increasing, with marked regional, national, and SDI-related disparities. Physiological characteristics, lifestyle changes, and healthcare accessibility are key drivers. Targeted interventions such as strengthening primary care, lifestyle guidance, and region-specific policies are critical to mitigate risks. This study fills a research gap, providing evidence to inform global strategies for GERD prevention and management in this population.

## 1 Introduction

GERD is a chronic digestive disorder defined as a condition characterized by the reflux of gastroduodenal contents into the esophagus, causing symptoms such as heartburn, acid regurgitation, and esophageal chest pain, or complications including reflux esophagitis and Barrett's esophagus (1–3). Its pathogenesis primarily involves an imbalance between weakened anti-reflux defenses and enhanced aggressive effects of refluxate (1, 4). Beyond typical esophageal symptoms, it presents with extra-esophageal manifestations such as laryngitis, refractory asthma, dental erosion, and chronic cough (5, 6). Based on esophageal mucosal histology, GERD is classified into non-erosive reflux disease, erosive esophagitis, and Barrett's esophagus, with diagnosis following the Montreal Global Consensus definition and classified under International Classification of Diseases (ICD), 10th Revision, using codes R12.11, K21–K21.9, and K22.7–K22.719 in the GBD 2021 study (3, 5, 7). Additionally, GERD may act as a significant risk factor for conditions including esophagitis, cancer, cardiovascular diseases, head and neck diseases, respiratory diseases, and mental disorders (1, 8–12). Globally, over 1 billion people are affected by GERD; its recurrent symptoms and complications severely impair health-related quality of life, and its chronicity and high prevalence impose substantial economic burdens on patients, families, healthcare systems, and society (13).

The GBD 2017 Gastroesophageal Reflux Disease Collaborators have systematically analyzed the global burden of GERD (13). Multiple published systematic reviews and cross-sectional studies have evaluated GERD incidence and prevalence globally and in specific regions or countries, with consistent findings indicating a growing global GERD burden (13, 14). Notably, previous studies have confirmed significant gender disparities in GERD, where compared with men, women consistently exhibit higher age-standardized incidence, prevalence, and YLDs, exacerbated by women-specific physiological and hormonal factors (13, 15). However, existing research primarily focuses on mechanisms underlying gender differences or is limited to small, region-specific samples, lacking systematic analysis of global epidemiological characteristics and burden trends among WCBA (16). It is worth noting that the COVID-19 pandemic, as a recent major public health event, may have impacted the epidemic trend of GERD among women of childbearing age, as increased sedentary behavior, higher intake of high-sugar and high-fat diets, and elevated psychological stress from home quarantine during the pandemic may have exacerbated their GERD risk, while the tilt of medical resources and restriction or delay of routine diagnosis and treatment may also have affected the disease burden (17).

WCBA in this study refers to females aged 15–49 years, an age range determined by the typical span of the female fertility physiological cycle. It takes into account three aspects: physiological aspects, where 15 years old is a landmark age when most women begin to have fertility; epidemiological characteristics, as women in this stage face a series of special health and psychological challenges related to reproduction; and public health policy formulation, where this age range is often used as the benchmark population for reproduction-related health services to facilitate targeted health interventions and management (18).

Given these unique physiological and social contexts, WCBA face unique challenges from GERD due to physiological traits such as hormonal fluctuations and pregnancy, as well as social roles such as childbearing and family responsibilities (19). During pregnancy, elevated estrogen and progesterone levels reduce lower esophageal sphincter tone, while mechanical compression of the stomach by the enlarging uterus significantly increases the incidence of GERD symptoms (20, 21). Additionally, WCBA often encounter lifestyle changes such as dietary adjustments and weight gain, psychological stress, medication use such as contraceptives, and medication restrictions such as cautious use of proton pump inhibitors during pregnancy, further complicating GERD prevention and management (16). Moreover, GERD not only compromises quality of life but may also raise the risk of pregnancy complications, including preterm birth and low birth weight, and exert lasting impacts on maternal and child health (17–19). Meanwhile, lifestyle factors such as sedentary behavior, obesity, and dietary habits, which have been identified as key modifiable risk factors in recent GBD analyses, further aggravate the burden of disease among this population (14).

Despite the significant clinical importance of GERD in WCBA, critical gaps remain in global burden research. First, existing epidemiological data are predominantly from Western countries, with limited studies and small samples in regions like Asia. Second, the impact of gender disparities on GERD burden is not fully elucidated, and while some studies suggest women experience more severe symptoms and greater psychological influence, global gender comparisons based on GBD data are lacking. Third, long-term trends such as age, period, and cohort effects and mechanisms underlying regional disparities, including SDI (Socio-demographic Index, a multi-dimensional tool quantifying national and regional development levels, calculated as the geometric mean of total fertility rate among women under 25, average years of schooling among adults, and lag-adjusted per capita income, ranging from 0 to 1 with higher values indicating better development and categorized into high, high-middle, middle, low-middle, and low SDI) in GERD among WCBA, are under-explored (22).

The GBD database, the most comprehensive global health data platform, covers disease burden data from 1990 to 2021 across 204 countries and territories, including core indicators such as incidence, prevalence, YLDs (years lived with disability, a key indicator measuring non-fatal health loss due to diseases, injuries, or health conditions), and disability-adjusted life years (DALYs, a key indicator measuring total health loss due to diseases, injuries, or health conditions, integrating both fatal health loss from premature death and non-fatal health loss captured by YLDs) (23). Its standardized methodologies, such as the DisMod-MR 2.1 model and multi-dimensional analytical frameworks such as age-period-cohort models, provide a scientific basis for systematically evaluating the global burden of GERD in WCBA. DisMod-MR 2.1 is a Bayesian meta-regression tool for disease modeling, which can generate internally consistent estimates of prevalence, incidence, remission rate, and mortality by gender, location, year, and age group. The tool also provides these measurements for locations lacking original epidemiological data by estimating cascading prevalence across the five levels of the GBD geographical hierarchy. Epidemiological data from higher-level locations serve as priors for estimating epidemiological parameters of lower-level locations. Additionally, DisMod-MR 2.1 uses covariates such as SDI to inform the prevalence and incidence in locations with missing data.

Based on the above research background and analysis, we propose the following core research hypothesis: The global burden of GERD among women of childbearing age has shown a continuous growth trend over the past three decades. Notably, this growth exhibits significant heterogeneity across different geographical regions and areas with varying levels of socioeconomic development. Furthermore, through the analysis of epidemiological data, we predict that the GERD burden in this population will continue to rise by 2050.

By integrating GBD data, this study aims to quantify the burden of GERD (including incidence, prevalence, and YLDs) among WCBA at global, regional, and national levels from 1990 to 2021; to analyze trends in burden and driving factors from 1990 to 2021, and explore the impact of SDI on burden distribution; and to identify high-burden regions and propose targeted intervention strategies.

This study fills existing research gaps, provides critical data support for global GERD prevention and management in WCBA, assists health policymakers in optimizing resource allocation, promotes evidence-based public health interventions such as lifestyle guidance and pregnancy management guidelines, and offers methodological references for subsequent studies to advance GERD epidemiological research.

## 2 Materials and methods

### 2.1 Study design and data sources

This study is a retrospective observational study using secondary data from the GBD Study 2021, focusing on trend analysis and future projections of GERD burden among WCBA. The GBD Study 2021 comprehensively evaluates the burden of 371 diseases, injuries, and health conditions, as well as 88 risk factors across 204 countries and territories, 21 regions, and 5 SDI levels, utilizing the most recent epidemiological data and refined standard methods to guarantee precision and thoroughness (24–26). To ensure data reliability, the data sources for the 2021 GBD study strictly adhered to inclusion and exclusion criteria. Included sources encompassed routine statistics, surveillance observations, literature reviews, etc., all of which were required to conform to standardized definitions and be compatible with core modeling tools. Non-public, low-quality, and methodologically inconsistent sources were excluded, along with data that could not estimate core outcomes, were incompatible with modeling, introduced interference, posed challenges in quantifying uncertainty, or exhibited significant bias, thereby safeguarding the reliability of the results (24, 27). Specifically, we obtained data on GERD in women aged 15–49 years from 1990 to 2021, focusing on incidence, prevalence, YLDs, and their corresponding ASRs stratified by age groups (15–19, 20–24, 25–29, 30–34, 35–39, 40–44, and 45–49 years), regions, and countries. All data in this study are derived from the official GBD 2021 database, which can be accessed through the relevant links on the Global Health Data Exchange (GHDx) platform: https://vizhub.healthdata.org/gbd-results/ (data query entry) and https://ghdx.healthdata.org/gbd-2021 (data catalog). To ensure the rigor of the methodology, data analysis strictly follows the standard operating procedures of GBD research, and various statistical methods are used to achieve a robust interpretation of the data. Each step of the analysis process complies with the requirements of the GATHER guidelines for cross-sectional studies. For specific data processing procedures, see the flowchart (Supplementary Figure S1). In addition, the research protocols and statistical codes related to GERD estimation can be obtained from the website: https://ghdx.healthdata.org/gbd-2021/code. Detailed information on incidence, prevalence, and YLDs can be accessed via the IHME website (http://ghdx.healthdata.org/gbd-results-tool) (28). Each estimate is presented as counts and age-standardized rates per 100,000 population, with 95% uncertainty intervals (UI) (29). Specifically, the 95% uncertainty intervals (UI) are calculated by generating 100 random draws from the estimated distribution and taking the 2.5th and 97.5th percentiles of these 100 draws (29).

### 2.2 Statistical Methods

This study applied Joinpoint Regression Analysis using Joinpoint Regression Software (version 4.9.0.0) to calculate the average annual percentage change (AAPC) and annual percentage change (APC) in age-standardized rates (ASRs) of GERD among women of childbearing age (WCBA) from 1990 to 2021, achieved by fitting a series of connected line segments (30). The model posits that temporal trends are manifested through a limited number of joinpoints, with log-linear trends existing between consecutive joinpoints (31). The minimum number of joinpoints required for model fitting is determined via Monte Carlo permutation tests to ensure statistically significant goodness of fit (*p* < 0.05), and their selection is based on the criterion of minimizing the mean squared error (MSE) using the grid search method (31). To guarantee the reproducibility of the analysis, the maximum number of joinpoints is restricted to 4, and the robustness of this model is verified through grid search and Monte Carlo tests (31). Frontier Analysis was utilized to evaluate the relationship between GERD burden in WCBA and sociodemographic development levels (32). This was done by developing frontier models based on age-standardized incidence rates (ASIR), age-standardized prevalence rates (ASPR), age-standardized years lived with disability rates (ASYR), and the sociodemographic index (SDI). The core of this method lies in identifying the theoretically attainable minimum ASR value for each country or region based on its current development level, which serves as a benchmark for optimal performance (32). It quantifies the gap between a country or territory's current burden and its potential minimum burden, thereby pinpointing areas where improvements can be made. The Bayesian Age-Period-Cohort (BAPC) model was employed to project the burden of GERD in WCBA through 2050. Constructed within the framework of the Generalized Linear Model (GLM), this model assumes that age, period, and cohort effects evolve over time, and its predictions are optimized through second-order random walk smoothing and the Integrated Nested Laplace Approximation (INLA) method (33). The BAPC model presumes the independence of age, period, and cohort effects, with prior distributions derived from historical data (33). Its validity is confirmed by back-testing projections for 2010–2021 against observed Global Burden of Disease (GBD) data, showing a deviation of <0.05.

### 2.3 Statistical Analysis

GERD burden in WCBA was quantified using incidence, prevalence, and YLDs (with 95% UI), with annual ASRs of these metrics stratified by age group, region, country, and SDI. ASRs (per 100,000 population) were standardized using the global standard population to enable comparisons across populations with different age structures (34). For joinpoint regression, after model fitting, AAPC, APC, and their 95% confidence intervals (CI) were calculated for each trend segment; trends were classified as upward (95% CI lower bound >0), downward (upper bound <0), or stable (CI includes 0) (35). For BAPC, interactions of age, period, and cohort effects were analyzed using R packages (BAPC and INLA), with future trends predicted by incorporating population structure changes and exponential growth patterns (33). All analyses were performed using R software (version 4.3.1) with a significance level of *p* < 0.05.

## 3 Results

### 3.1 The global burden and the trend of GERD among WCBA

**Figure 1:**
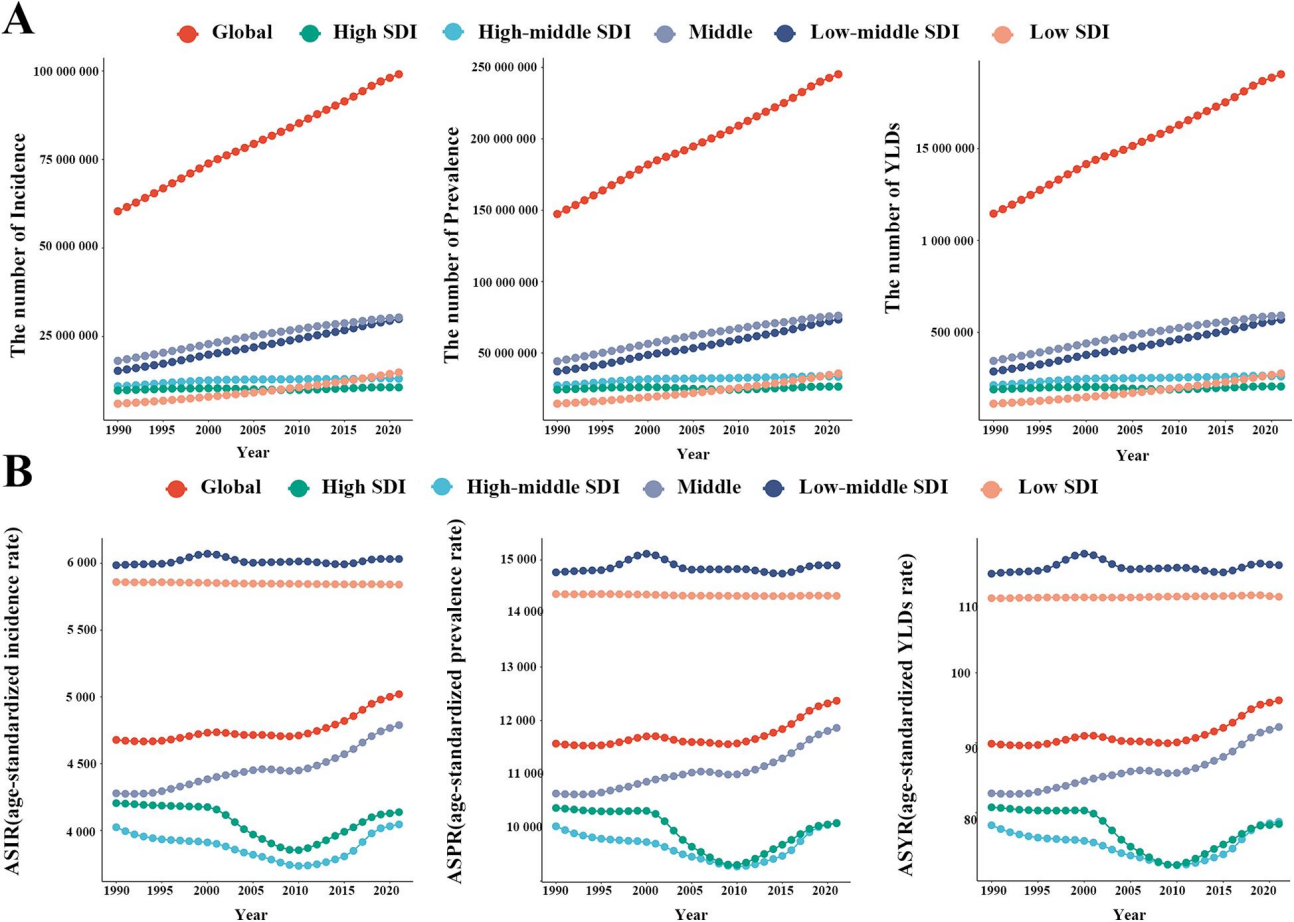
The number and age-standardized rate of incidence, prevalence, and YLDs of gastroesophageal reflux disease in women of childbearing age globally and in different SDI regions from 1990 to 2021. **(A)** Number; **(B)** Age-standardized rate. ASIR, age-standardized incidence rate; ASPR, age-standardized prevalence rate; YLDs, years lived with disability; ASYR, age-standardized YLD rate; SDI, sociodemographic index.

Globally, an estimated 99,063,156 incident cases (95% UI: 70,003,846–132,649,268) and 245,243,624 prevalent cases (95% UI: 178,638,601–325,359,465) of GERD were reported in WCBA in 2021, representing increases of 64.09% and 66.44% since 1990, respectively (Figure 1A; Table 1). From 1990 to 2021, the global ASIR of GERD in WCBA increased from 4,677.86 per 100,000 (95% UI: 3,293.34–6,286.58) to 5,020.12 per 100,000 (95% UI: 3,548.92–6,725.79), with an AAPC of 0.24 (95% CI: 0.21–0.27) (Figure 1B; Table 1). The ASPR rose from 11,568.5 per 100,000 (95% UI: 8,409.18–15,401.05) to 12,369.87 per 100,000 (95% UI: 9,013.75–16,410.22), with an AAPC of 0.23 (95% CI: 0.19–0.27) (Figure 1B; Table 1). In 2021, global YLDs due to GERD in WCBA were 1,904,157 (95% UI: 911,835–3,571,347), with the ASYR of 96.08 per 100,000 (95% UI: 45.97–180.08) (Figure 1; Table 1). Both YLDs and ASYR increased from 1990 to 2021, with an ASYR AAPC of 0.23 (95% CI: 0.19–0.27) (Figure 1; Table 1).

**Table 1:** Incidence, prevalence, and YLDs of gastroesophageal reflux disease in women of childbearing age in 1990 and 2021, and estimated average annual percentage changes from 1990 to 2021.

Location	Incidence					Prevalence					YLDs				
	Number of cases, 1990	ASIR per 100,000 people, 1990 (95% UI)	Number of cases, 2021	ASIR per 100,000 people, 2021 (95% UI)	AAPC, 1990 to 2021 (95% CI)	Number of cases, 1990	ASPR per 100,000 people, 1990 (95% UI)	Number of cases, 2021	ASPR per 100,000 people, 2021 (95% UI)	AAPC, 1990 to 2021 (95% CI)	Number of cases, 1990	ASYR per 100,000 people, 1990 (95% UI)	Number of cases, 2021	ASYR per 100,000 people, 2021 (95% UI)	AAPC, 1990 to 2021 (95% CI)
Global	60,372,888 (42,591,738, 81,200,618)	4,677.86 (3,293.34, 6,286.58)	99,063,156 (70,003,846, 132, 649,268)	5,020.12 (3,548.92, 6,725.79)	0.24 (0.21, 0.27)	147,349,101 (107,247,564, 196,093,930)	11,568.5 (8,409.18, 15,401.05)	245,243,624 (178,638, 601,325,359,465)	12,369.87 (9,013.75, 16,410.22)	0.23 (0.19, 0.27)	1,145,812 (546,056, 2,151,858)	89.86 (42.9, 169.1)	1,904,157 (911,835, 3,571,347)	96.08 (45.97, 180.08)	0.23 (0.19, 0.23)
SDI															
High SDI	9,792,213 (6,828,698, 13,273,219)	4,204.43 (2,931.51, 5,701.79)	10,657,619 (7,428,425, 14,460,626)	4,136.94 (2,890.19, 5,613.73)	−0.03 (−0.09, 0.03)	24,315,929 (17,583,221, 32,789,909)	10,366.09 (7,495.43, 13,970.19)	26,419,774 (18,850,722, 35,837,563)	10,079.89 (7,203.44, 13,648.98)	−0.06 (−0.13, −0.01)	189,428 (91,131, 357,594)	80.77 (38.85, 152.41)	205,216 (97,925, 387,224)	78.38 (37.28, 147.37)	−0.06 (−0.14, −0.01)
High-middle SDI	10,983,414 (7,675,030, 14,945,342)	4,023.86 (2,806.09, 5,477.45)	13,096,680 (9,252,273, 17,741,198)	4,045.19 (2,870.16, 5,483.25)	−0.07 (−0.02, 0.15)	27,203,913 (19,649,571, 36,712,845)	10,024.7 (7,236.44, 13,532.95)	33,387,042 (24,538,007, 44,501,084)	10,089.09 (7,428.86, 13,434.25)	0.07 (−0.02, 0.16)	212,390 (100,565, 399,455)	78.21 (37.1, 147.32)	260,103 (125,344, 486,169)	78.72 (37.82, 146.62)	0.07 (−0.02, 0.16)
Middle SDI	18,152,512 (12,905,446, 24,251,789)	4,277.93 (3,035.79, 5,700.01)	30,400,560 (21,612,071, 40,395,838)	4,788.5 (3,406.01, 6,373.64)	0.38 (0.34, 0.41)	44,179,171 (32,193,485, 58,426,670)	10,635.81 (7,744.67, 14,062.93)	76,110,400 (55,446,948, 100,488,252)	11,862.52 (8,646.5, 15,663.16)	0.36 (0.33, 0.39)	344,371 (164,341, 643,794)	82.77 (39.63, 154.99)	591,478 (283,942, 1,110,879)	92.25 (44.22, 173.12)	0.36 (0.33, 0.4)
Low-middle SDI	15,329,654 (10,866,755, 20,459,028)	5,985.66 (4,238.46, 7,964.11)	29,955,896 (21,243,596, 39,941,171)	6,032.14 (4,274.7, 8,036.71)	0.02 (−0.02, 0.06)	37,023,913 (26,937,847, 48,906,363)	14,769.67 (10,726.54, 19,510.21)	73,485,563 (53,431,457, 97,067,929)	14,900.43 (10,825.76, 19,680.16)	−0.02 (−0.04, 0.08)	286,701 (137,493, 536,442)	114.18 (54.9, 214.21)	569,523 (273,160, 1,065,711)	115.4 (55.42, 216.14)	0.03 (−0.03, 0.09)
Low SDI	6,049,336 (4,261,569, 8,158,289)	5,859.41 (4,116.2, 7,893.99)	14,868,140 (10,470,481, 20,053,078)	5,840.64 (4,103.46, 7,867.49)	−0.01 (−0.01, −0.01)	14,462,266 (10,458,542, 19,264,727)	14,363.81 (10,355.93, 19,167.97)	35,626,726 (25,757,271, 47,473,100)	14,329.17 (10,333.75, 19,123.75)	−0.01 (−0.01, −0.01)	111,645 (53,026, 208,093)	110.68 (52.72, 207.47)	276,171 (131,037, 515,852)	110.88 (52.79, 208.06)	0.01 (0, 0.01)

YLDs, years lived with disability; ASIR, age-standardized incidence rate; ASPR, age-standardized prevalence rate; ASYR, age-standardized YLD rate; UI, uncertainty interval; AAPC, average annual percentage change; CI, confidence interval.

**Figure 2:**
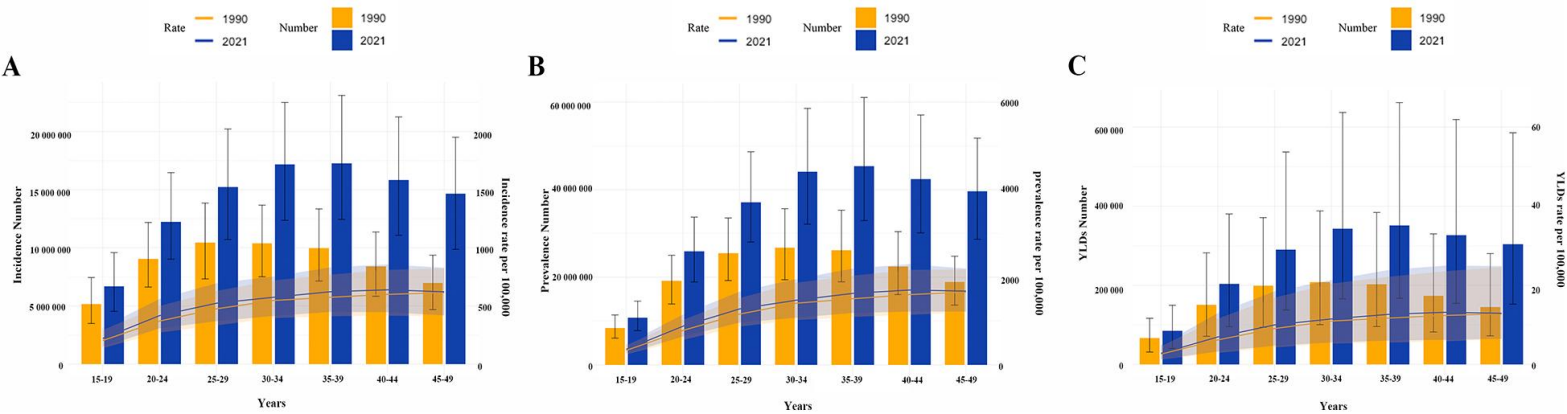
Age-specific number and rate of incidence, prevalence, and YLDs of gastroesophageal reflux disease in women of childbearing age in 1990 and 2021. **(A)** Incidence; **(B)** Prevalence; **(C)** YLDs. YLDs, years lived with disability.

**Figure 3:**
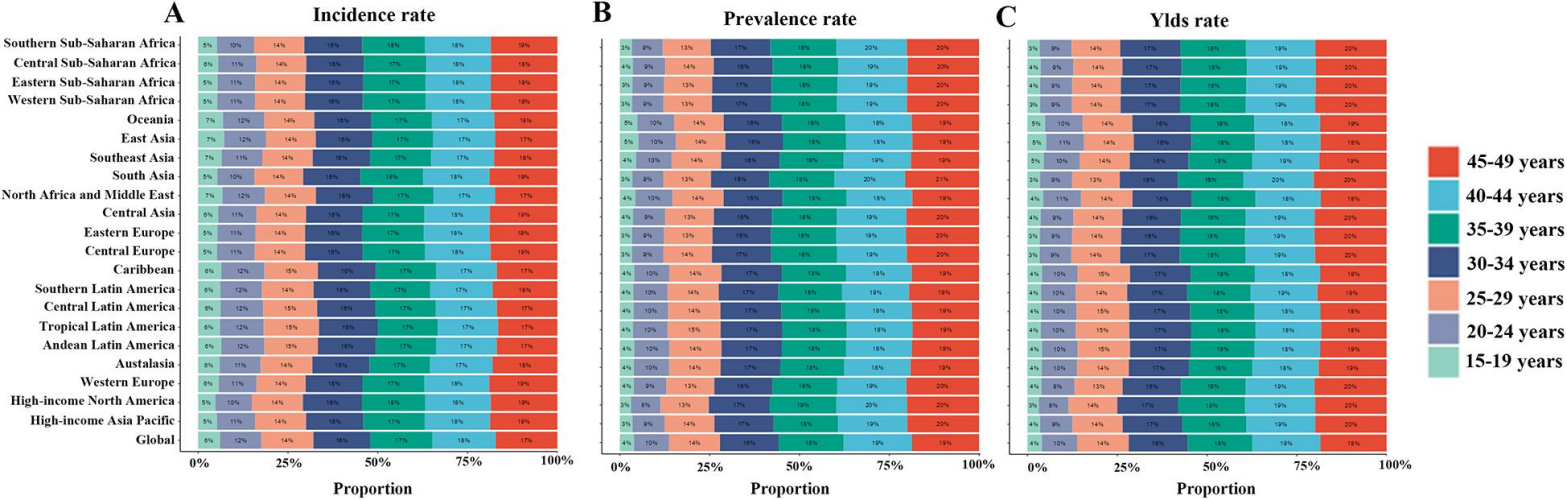
Age-specific percentages of incidence, prevalence, and YLDs of gastroesophageal reflux disease in women of childbearing age across 21 regions and globally in 2021. **(A)** Incidence rate; **(B)** Prevalence rate; **(C)** YLD rate. YLDs, years lived with disability.

Globally, stratification of WCBA with GERD by 5-year age groups showed that incidence, prevalence, and YLD rates increased gradually with age, plateauing after the 25–29 years group (Figures 2,3). In 2021, rates rose gradually in the 15–19, 20–24, and 25–29 years groups, with smaller differences among the 30–34, 35–39, 40–44, and 45–49 years groups (Figures 2,3). For example, incidence rates across age groups in 2021 were 6%, 12%, 14%, 16%, 17%, 18%, and 17%, respectively. Regional trends were generally consistent with the global pattern (Figure 3).

### 3.2 The regional burden and the trend of GERD among WCBA

**Figure 4:**
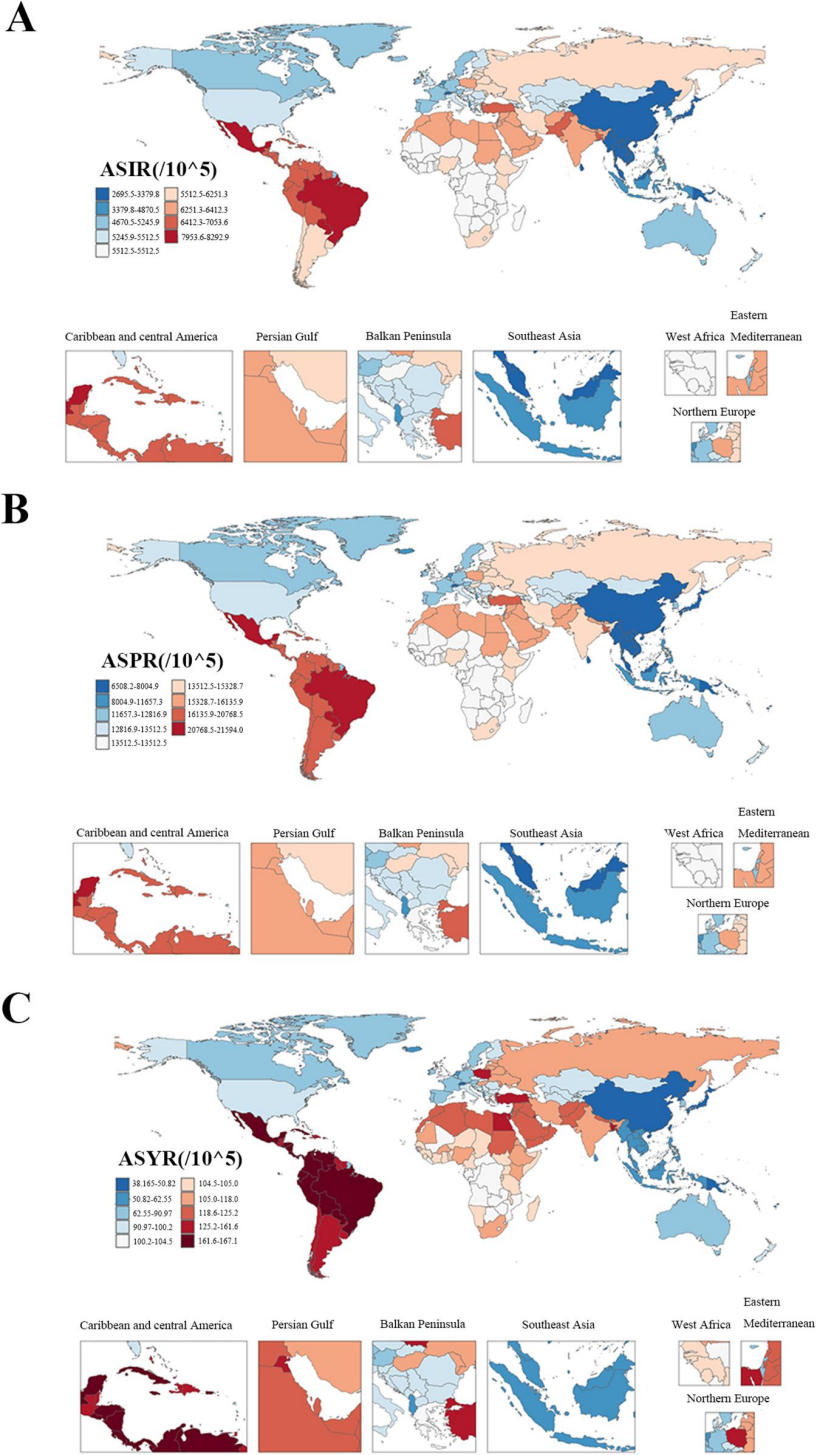
The ASRs of incidence, prevalence, and YLDs of gastroesophageal reflux disease in women of childbearing age among countries and territories in 2021. (**A**) ASIR; **(B)** ASPR; **(C)** ASYR. ASR, age-standardized rate; ASIR, age-standardized incidence rate; ASPR, age-standardized prevalence rate; YLDs, years lived with disability; ASYR, age-standardized YLD rate.

Among the 21 regions covered by the GBD 2021 study, South Asia had the highest absolute burden of GERD among WCBA in 2021, with 30,956,482 incident cases (95% UI: 21,751,940–41,542,386), 75,194,636 prevalent cases (95% UI: 54,259,710–100,075,430), and 582,074 YLDs (95% UI: 279,765–1,102,621) (Supplementary Tables S1–S3). Oceania had the lowest, with 91,898 new cases (95% UI: 62,917–126,828), 220,454 prevalent cases (95% UI: 155,746–305,093), and 1,720 YLDs (95% UI: 802–3,255) (Supplementary Tables S1–S3). In terms of ASRs, Tropical Latin America had the highest ASIR (8,287.96 per 100,000, 95% UI: 6,065.62–10,575.44), ASPR (21,591.02 per 100,000, 95% UI: 16,003.23–27,685.91), and ASYR (167.07 per 100,000, 95% UI: 81–305.32) (Figure 4; Supplementary Tables S1–S3). East Asia had the lowest ASIR (2,322.89 per 100,000, 95% UI: 1,578.78–3,246.17), ASPR (5,639.71 per 100,000, 95% UI: 3,951.4–7,792.01), and ASYR (44.32 per 100,000, 95% UI: 20.72–84.5) (Figure 4; Supplementary Tables S1–S3). From 1990 to 2021, High-income North America showed the most significant increases in ASIR(AAPC = 0.07, 95% CI: 0.02–0.12), ASPR(AAPC = 0.07, 95% CI: 0.02–0.11), and ASYR (AAPC = 0.07, 95% CI: 0.02–0.12), while Andean Latin America showed the largest decreases (ASIR AAPC = −0.47, 95% CI: −0.60 to −0.33; ASPR AAPC = −0.45, 95% CI: −0.60 to −0.30; ASYR AAPC = −0.47, 95% CI: −0.60 to −0.33) (Supplementary Figure S2, Tables S1–S3).

### 3.3 The national burden and the trend of GERD among WCBA

Among the 204 countries and territories included in 2021, the Republic of India had the highest incidence (23,565,252 cases, 95% UI: 16,553,672–31,745,151); the People's Republic of China had the highest prevalence (19,491,437 cases, 95% UI: 13,595,210–27,027,515); and the Republic of India had the most YLDs (441,264 years, 95% UI: 212,534–834,357) (Supplementary Tables S4–S6). In terms of ASRs, the Federative Republic of Brazil had the highest ASIR (8,292.86 per 100,000, 95% UI: 6,066.62–10,575.88), ASPR (21,594 per 100,000, 95% UI: 16,001.78–27,686.18), and ASYR (167.08 per 100,000, 95% UI: 80.99–305.37) (Supplementary Tables S4–S6). The Kingdom of Norway had the lowest ASIR (2,151.27 per 100,000, 95% UI: 1,457.92–2,998.37), ASPR (4,903.75 per 100,000, 95% UI: 3,435.79–6,783.27), and ASYR (38.17 per 100,000, 95% UI: 17.73–72.52) (Supplementary Tables S4–S6). From 1990 to 2021, the Republic of Turkey showed the most significant increases in ASIR, ASPR, and ASYR (AAPC = 0.18, 95% CI: 0.15–0.22; AAPC = 0.27, 95% CI: 0.23–0.32; AAPC = 0.27, 95% CI: 0.24–0.30, respectively) (Supplementary Tables S4–S6). The United States of America showed the largest decreases (AAPC = −0.37, 95% CI: −0.46 to −0.28; AAPC = −0.48, 95% CI: −0.64 to −0.33; AAPC = −0.50, 95% CI: −0.64 to −0.36, respectively) (Supplementary Tables S4–S6).

### 3.4 The burden of GERD among WCBA at the SDI regional level and its trends

**Figure 5:**
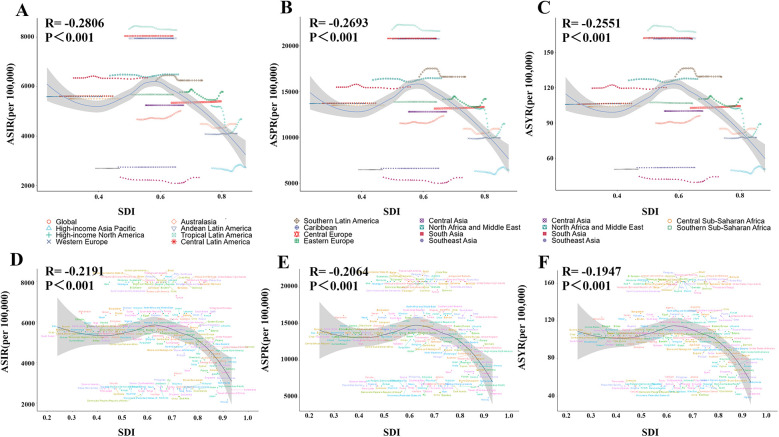
The relationship between ASIR/ASPR/ASYR of gastroesophageal reflux disease in women of childbearing age and SDI across 21 regions and 204 countries in 2021. **(A/D)** ASIR; **(B/E)** ASPR; **(C/F)** ASYR. ASIR, age-standardized incidence rate; ASPR, age-standardized prevalence rate; YLDs, years lived with disability; ASYR, age-standardized YLD rate; SDI, sociodemographic index.

This study also explored GERD burden in WCBA across five SDI regions (Figure 5; Table 1). In 2021, middle SDI regions had the highest absolute burden, with 30,400,560 incident cases (95% UI: 21,612,071–40,395,838), 76,110,400 prevalent cases (95% UI: 55,446,948–100,488,252), and 591,478 YLDs (95% UI: 283,942–1,110,879) (Figure 1A; Table 1). High SDI regions had the lowest, with 10,657,619 new cases (95% UI: 7,428,425–14,460,626), 26,419,774 prevalent cases (95% UI: 18,850,722–35,837,563), and 205,216 YLDs (95% UI: 97,925–387,224) (Figure 1A; Table 1). In terms of age-standardized rates, low-middle SDI regions had the highest ASIR (6,032.14 per 100,000, 95% UI: 4,274.7–8,036.71), ASPR (14,900.43 per 100,000, 95% UI: 10,825.76–19,680.16), and ASYR (115.4 per 100,000, 95% UI: 55.42–216.14), while high and high-middle SDI regions had lower ASIR, ASPR, and ASYR (Figure 1B; Table 1).

From 1990 to 2021, ASIR, ASPR, and ASYR showed no significant overall trends in high, high-middle, low-middle, and low SDI regions, but increased significantly in middle SDI regions (ASIR AAPC = 0.38, 95% CI: 0.34–0.41; ASPR AAPC = 0.36, 95% CI: 0.33–0.39; ASYR AAPC = 0.36, 95% CI: 0.33–0.40) (Figure 1B; Table 1). Analysis of associations between ASIR, ASPR, ASYR, and SDI across 21 regions and 204 countries from 1990 to 2021 showed that GERD burden in WCBA decreased with increasing SDI (Figure 5). At the regional level, GERD burden showed a “gradual decline–gradual rise–redecline” pattern with increasing SDI, with an overall negative correlation (ASIR: *R* = −0.2806, *p* < 0.001; ASPR: *R* = −0.2693, *p* < 0.001; ASYR: *R* = −0.2551, *p* < 0.001), and trends in ASIR, ASPR, and ASYR were broadly consistent (Figure 5). At the national level, ASIR, ASPR, and ASYR in WCBA were also negatively correlated with SDI (ASIR: *R* = −0.2191, *p* < 0.001; ASPR: *R* = −0.2064, *p* < 0.001; ASYR: *R* = −0.1947, *p* < 0.001), with ASR trends similar to those at the regional level (Figure 5).

### 3.5 Temporal joinpoint analysis of GERD burden among WCBA

**Figure 6:**
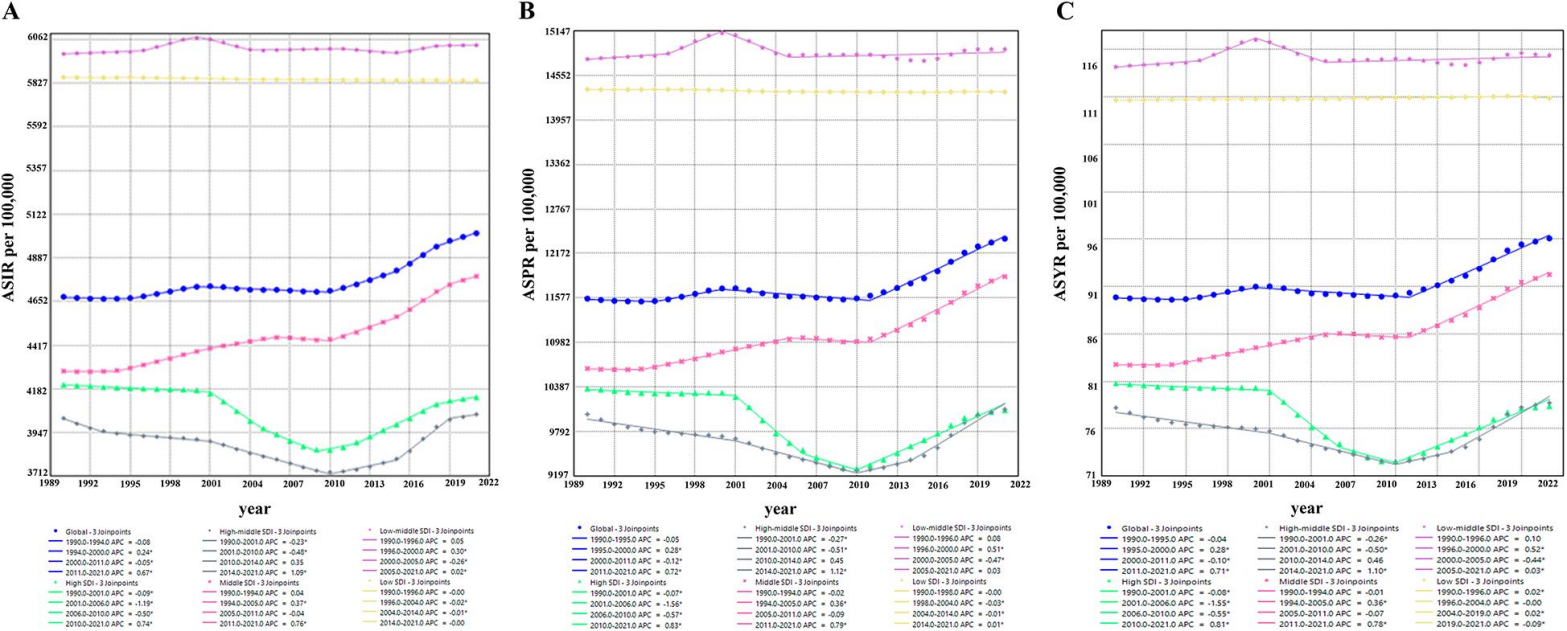
Joinpoint regression analysis of ASIR, ASPR, and ASYR for gastroesophageal reflux disease in women of childbearing age in different SDI regions from 1990 to 2021. **(A)** ASIR in different SDI regions; **(B)** ASPR in different SDI regions; **(C)** ASYR in different SDI regions. ASIR, age-standardized incidence rate; ASPR, age-standardized prevalence rate; YLDs, years lived with disability; ASYR, age-standardized YLD rate; SDI, sociodemographic index.

Joinpoint regression analysis showed overall upward trends in global ASIR, ASPR, and ASYR for GERD in WCBA from 1990 to 2021 (ASIR: AAPC = 0.24, 95% CI: 0.21–0.27, *p* < 0.001; ASPR: AAPC = 0.23, 95% CI: 0.19–0.27, *p* < 0.001; ASYR: AAPC = 0.23, 95% CI: 0.19–0.27, *p* < 0.001) (Figure 6; Table 1). ASIR, ASPR, and ASYR showed slight declines in 1990–1994 and 2000–2011, and increases in 1994–2000 and 2011–2021, with significant acceleration in 2011–2021 (ASIR: AAPC = 0.67; ASPR: AAPC = 0.72; ASYR: AAPC = 0.71) (Figure 6). Joinpoint regression by SDI showed that ASIR, ASPR, and ASYR fluctuated slightly but remained stable overall in low-middle and low SDI regions from 1990 to 2021; middle SDI regions showed a clear upward trend across all periods; and high and high-middle SDI regions showed a “stable–decline–rise” pattern. Trends in ASIR, ASPR, and ASYR were generally consistent globally and across SDI regions (Figure 6).

### 3.6 Frontier analysis of GERD burden among WCBA and national development

**Figure 7:**
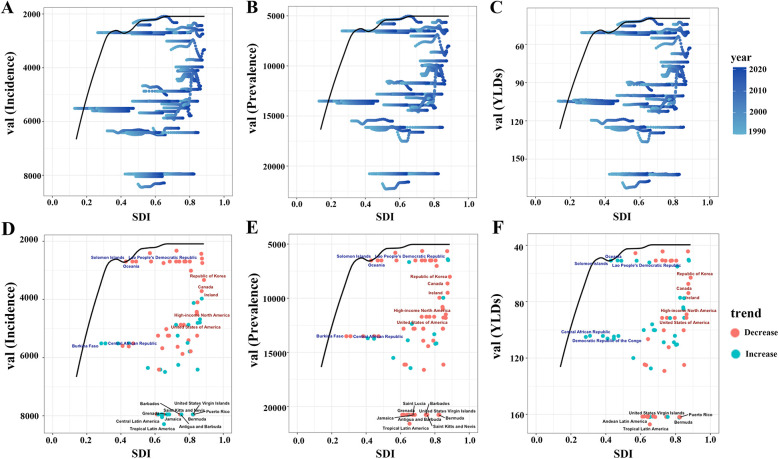
Frontier analysis of SDI and ASIR/ASPR/ASYR of gastroesophageal reflux disease in women of childbearing age in 204 countries and regions. **(A)** Trajectories of ASIR (per 100,000) of gastroesophageal reflux disease in women of childbearing age in 204 countries from 1990 to 2021. The black curve marks the frontier—the lowest burden observed at each level of the SDI. **(D)** Country-level ASIR (per 100,000) of gastroesophageal reflux disease in women of childbearing age in 2021 plotted against the same frontier. **(B/E)** Corresponding plots for ASPR. **(C/F)** Corresponding plots for ASYR. ASIR, age-standardized incidence rate; ASPR, age-standardized prevalence rate; YLDs, years lived with disability; ASYR, age-standardized YLD rate; SDI, sociodemographic index.

Frontier analysis explored relationships between ASIR, ASPR, ASYR, and SDI from 1990 to 2021 to investigate associations between GERD burden in WCBA and national development status (Figure 7). We adopted the lowest ASIR, ASPR, and ASYR among high SDI countries as “optimal burden levels” and examined the differences between each country's burden and these optimal benchmarks (Figure 7). This highlighted considerable scope for improving disease intervention and management (Figure 7). Analysis of SDI and ASYR identified countries or regions with significant deviations between SDI and expected disease burden, primarily low-middle SDI countries such as Oceania, the Central African Republic, and the Democratic Republic of the Congo (Figure 7). These countries had health metrics far above the frontier, indicating heavy, poorly controlled burdens (Figure 7). In contrast, high SDI countries such as the Republic of Korea and Canada had relatively low burdens but still exceeded theoretical minimums (Figure 7). Additionally, health metrics in the United States and Tropical Latin America showed no significant improvement with increasing SDI, deviating markedly from expected trends (Figure 7). Results indicated that high SDI countries were generally closer to the efficiency frontier, while low-middle SDI countries lagged significantly (Figure 7). Burden disparities among high SDI countries highlighted uneven progress in reducing GERD burden in WCBA across SDI levels, emphasizing the need for targeted public health policies and resource optimization to narrow global health gaps (Figure 7).

### 3.7 Projections of global GERD burden among WCBA

**Figure 8:**
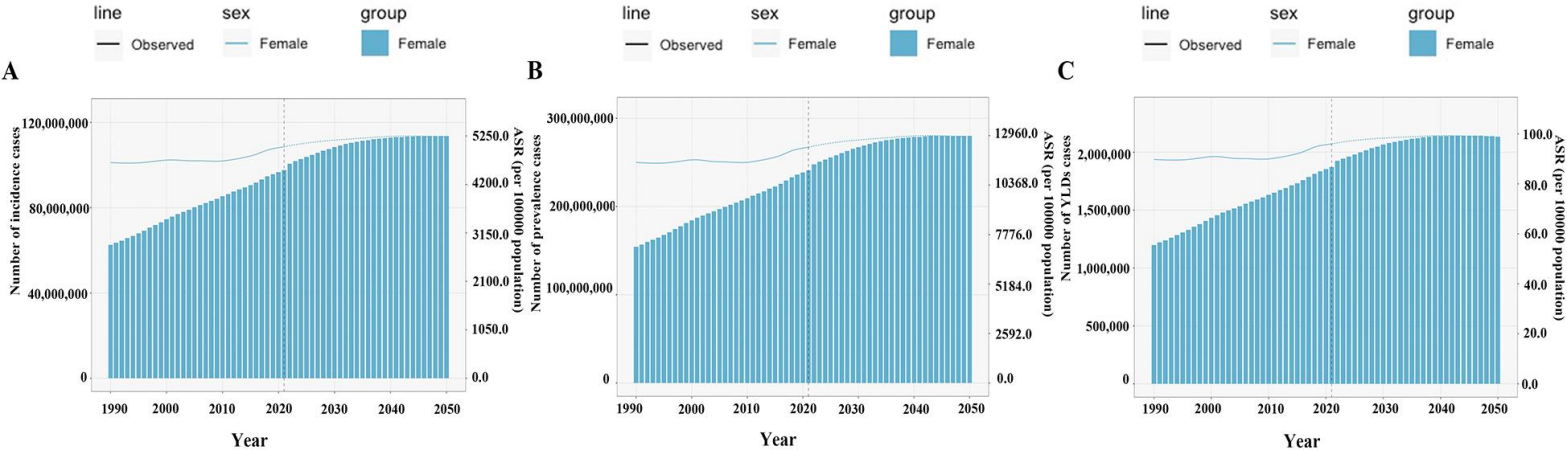
The predicted burden of gastroesophageal reflux disease in women of childbearing age globally to 2050. **(A)** Incidence; **(B)** Prevalence; **(C)** YLDs; ASR, age-standardized rate. YLDs, years lived with disability.

BAPC modeling projected a continued upward trend in global GERD burden in WCBA by 2050 (Figure 8). Specifically, incidence is projected to reach 113,550,943 cases, prevalence 280,016,399 cases, and YLDs 713,433 years by 2050; ASIR, ASPR, and ASYR are projected to be 5,229.11 per 100,000, 12,894.97 per 100,000, and 98.35 per 100,000, respectively (Figure 8).

## 4 Discussion

Based on the GBD 2021 database, this study is the first to systematically analyze the burden, spatiotemporal trends, and future projections of GERD among WCBA at global, regional, and national levels from 1990 to 2021, revealing key characteristics of GERD burden in this specific population. Previous studies have focused on the general population, children, the elderly, or mechanistic research, whereas this study targets WCBA, a group with unique risk factors and disease pathogenesis (4, 36, 37). Results showed that global incidence, prevalence, and YLDs of GERD in WCBA are increasing, with significant regional disparities, a negative correlation with SDI, specific age patterns, and projected future increases, providing critical insights for understanding the epidemiology of GERD in this population and formulating targeted prevention strategies.

Over the past three decades, GERD burden in this population has increased continuously, characterized by dual rises in case counts and ASRs. Despite modest increases in ASIR, ASPR, and ASYR, absolute case counts (64.09% increase in incidence, 66.44% in prevalence) and YLDs grew significantly, closely linked to global population growth (especially expansion of the childbearing-aged cohort) and urbanization-driven sedentary behavior, high-fat, high-sugar diets, and rising obesity rates (13, 14). The increase in absolute GERD burden in WCBA may result from both demographic changes and shifts in chronic disease epidemiology, while improved early detection and diagnosis due to advances in global healthcare may partially explain the rise in standardized rates (13). Additionally, delayed childbearing and population growth globally may extend this high-risk window (38). Notably, acceleration post-2011 (AAPCs 0.67–0.72) may relate to increased sedentary behavior from smartphone use, westernization of high-fat and high-sugar diets, and heightened stress factors particularly impactful for WCBA (13, 14). We also found that age distribution showed a “gradual rise after 25, plateau after 40” pattern, linked to pregnancy-related hormonal changes such as progesterone reducing lower esophageal sphincter tone and parity. To address this, specific data on SDI-stratified risk factors have been incorporated into the paper. Among these, the 25–29 age group marks a critical starting point for the rising burden, directly linked to physiological changes triggered by the peak of first pregnancies. The age-standardized rate in the 30–34 age group further increases and remains elevated, a trend not only associated with the sustained peak pregnancy rate in this stage but also compounded by social factors such as irregular diets and weight gain stemming from work pressure and family responsibilities (19). These age-specific patterns profoundly reflect the unique vulnerabilities of women of childbearing age across different life stages. For young women aged 25–29, risks are centered on pregnancy-related physiological changes. In low-SDI regions with limited resources, insufficient basic healthcare further amplifies such vulnerabilities (39). Women aged 35 and above, in particular, gradually face the accumulation of metabolic risks, including the interplay of unhealthy dietary habits, sedentary lifestyles, and obesity-induced elevated intra-abdominal pressure with weakened esophageal function—collectively forming age-corresponding risk profiles (40). Additionally, women of childbearing age face specific restrictions on medication use. For instance, proton pump inhibitors (PPIs) require cautious use during pregnancy, which increases treatment complexity, prolongs the treatment course, and further contributes to a higher number of affected individuals across all age groups (20). This stratified characteristic suggests that interventions should consider both life stages and resource conditions. For example, integrating antenatal GERD management into prenatal care in low-SDI regions and enhancing metabolic risk screening for middle-aged women in middle-high SDI regions. Joinpoint analysis reveals that the burden of GERD increased significantly after 2011, which may be associated with factors such as global population growth (particularly WCBA) and accelerated urbanization since 2011 (13, 15).

Global GERD burden in WCBA shows marked national and regional differentiation. Regionally, South Asia and Tropical Latin America have the highest absolute burdens, with Tropical Latin America having the highest ASRs (ASIR = 8,287.96 per 100,000), consistent with findings in the general population (13). South Asia's high burden may relate to its large population, dietary patterns such as spicy foods, low-fiber diets, and limited healthcare access; Tropical Latin America's high rates may reflect improved case detection from enhanced diagnostic capacity (13, 41, 42). In contrast, East Asia has the lowest standardized rates, potentially due to low obesity, traditional low-fat, high-fiber diets, early intervention, and a shrinking childbearing-aged cohort from aging (42, 43). High-Income North America showed significant burden increases, possibly from urban high-fat diets, rising obesity, and hidden cases due to PPI dependence; Latin America showed significant declines (AAPC = −0.47), potentially from strengthened public health interventions, preserved traditional diets, and integration of GERD screening into prenatal care (42, 44). This is consistent with the conclusion in other studies that unhealthy dietary habits are more prevalent in regions such as the Americas, which in turn leads to a higher burden of GERD in these regions (45). Obesity, a key modifiable risk factor, interacts with SDI to shape these patterns (42). High-SDI regions have higher obesity prevalence due to sedentary lifestyles and high-calorie diets, but their robust healthcare systems—including routine prenatal screening and accessible proton pump inhibitors—mitigate GERD severity, resulting in lower ASRs (46, 47). In contrast, low-middle SDI regions face a “double burden.” Rising obesity from rapid urbanization and Westernized diets—high in fat and low in fiber—coincides with limited access to anti-reflux therapies, driving up YLDs (42). Dietary habits further amplify disparities. South Asia's high consumption of spicy foods and low fiber intake exacerbate reflux, while East Asia's traditional low-fat diets and lower obesity rates contribute to its low ASRs (41, 42, 48).

Nationally, the Republic of India and the People's Republic of China's large case counts reflect their large populations, high fertility rates, lifestyle changes, and limited healthcare resources (49–51). Brazil's high ASRs may relate to unhealthy diets and uneven healthcare resource distribution (52). Norway's low rates may stem from early screening, such as incorporating GERD symptom assessment into routine care and prenatal care, robust healthcare systems, healthy lifestyles, and gender-equal social environments, offering a global reference for GERD control (13, 14). Over the past three decades, Turkey showed the most significant ASR increases, linked to westernized diets, rising obesity, uneven healthcare access, and inadequate pregnancy management; the United States showed the largest declines, benefiting from early screening, precise diagnosis, improved medication access and utilization, and comprehensive prenatal care, significantly mitigating GERD risk.

This study is the first to confirm a negative correlation between GERD burden in WCBA and SDI, contrary to the typical pattern of higher chronic disease burdens in high SDI regions (13, 20). This discrepancy may reflect disease-specific traits in that, as a lifestyle-related disorder, GERD is effectively controlled in high SDI regions through early diagnosis and interventions such as prenatal PPIs, while low-middle SDI regions face high rates due to rising obesity, weak pregnancy-related health management, and delayed diagnosis (14, 20). Secondly, the distribution of GERD risk factors also shows obvious SDI-stratified characteristics. High body mass index (BMI), as a key risk factor, is exacerbated in low and low-middle SDI regions. This is closely related to the westernization of diets and rising obesity rates amid urbanization in these regions, which is consistent with the conclusion in the WHO Global Nutrition Report that the prevalence of obesity in low-income regions continues to climb (https://www.who.int/news-room/fact-sheets/detail/obesity-and-overweight). In addition, in low and low-middle SDI regions, the widespread presence of diets low in vegetables and the consumption of chewing tobacco have become important factors exacerbating the GERD burden (42). Meanwhile, smoking, as a risk factor, has a more significant impact on GERD in high SDI regions due to the long history of tobacco consumption and high prevalence (15, 53). Such SDI-stratified differences in risk factors further exacerbate the imbalance in GERD burden across regions with different development levels. Middle SDI regions have the highest absolute case counts, low-middle SDI regions the highest standardized rates, and high SDI regions the lowest burden, potentially explained by factors such as earlier screening and treatment in high SDI regions, which effectively control disease progression; limited resources in low-middle SDI regions causing delayed diagnosis, inadequate treatment, persistent symptoms, and elevated YLDs, alongside high pregnancy rates and poor prenatal GERD management exacerbating burden in WCBA; and rapid urbanization in middle SDI regions driving dietary shifts (high-fat or high-sugar) and reduced physical activity, boosting obesity-related GERD risk (13, 14, 20, 42). This is consistent with the findings of other studies that the burden of GERD is the highest in low-middle SDI regions and shows a negative correlation with SDI, both reflecting the dual pressures of westernized lifestyle transition and insufficient medical resources (45). Notably, middle SDI regions such as Turkey show the steepest ASIR and ASPR increases (AAPCs 0.18–0.27), requiring prioritized prevention. Burden disparities across SDI regions highlight the significant impact of socioeconomic development on disease burden. Frontier analysis showed that low SDI countries' burdens far exceed SDI-based expectations, suggesting their healthcare systems should prioritize GERD screening, including popularizing symptom questionnaires and basic treatments such as antacids. High SDI countries, while having lower rates, still have unmet optimization potential, with a focus needed on managing obesity-related GERD. Healthcare access acts as a critical mediator (46, 54). High-SDI regions like Norway integrate GERD screening into prenatal care, enabling early intervention, while low-SDI regions with under-resourced primary care face delayed diagnosis, which prolongs symptoms and inflates prevalence (46, 55). These factors—obesity, diet, and healthcare access—interact with SDI to drive the observed trends, with middle-SDI regions like Turkey being most vulnerable due to unmanaged urbanization and transitioning lifestyles (42, 48).

Limitations of this study include that GBD estimates are limited by underlying data quality; incomplete or poor-quality data from some countries may introduce bias; although GBD uses advanced statistical algorithms for data calibration and modeling, the parameter settings and theoretical basis of the model itself may still have a significant impact on the calculation results of key indicators; the analysis focuses solely on WCBA, and future comparative studies including men and other age groups are needed to identify additional influencing factors; BAPC model projections are based on historical trends, and actual burdens may deviate if lifestyles or medical interventions change significantly. Despite potential model biases and missing data in GBD, these issues do not undermine the validity of overall trend analyses of GERD burden in WCBA.

## 5 Conclusions

This study reveals a continuous increase in global GERD burden among WCBA from 1990 to 2021, with significant disparities across regions, countries, and SDI levels. Key drivers include WCBA's physiological traits, such as pregnancy and hormonal fluctuations, and social factors such as lifestyle and healthcare accessibility. Future projections indicate a worsening burden by 2050 without targeted interventions. Findings emphasize the need to consider age, regional, and national differences in lifestyle and disease risk when formulating public health policies, with integrated strategies such as personalized lifestyle advice, public health interventions, health education, and economic incentives to mitigate disease impact. This study provides a scientific basis for global GERD prevention and control, highlights the importance of focusing on WCBA, and offers a foundation for formulating precise public health strategies.

## Data Availability

The original contributions presented in the study are included in the article/Supplementary Material, further inquiries can be directed to the corresponding author.
