# Supplementary material for: Burden of gastroesophageal reflux disease among women of childbearing age, with projections to 2050: an analysis of the Global Burden of Disease study 2021
Source: Front Glob Womens Health. 2025 Sep 19;6:1673878. doi: 10.3389/fgwh.2025.1673878 (PMC12491323; doi:10.3389/fgwh.2025.1673878)
Supplement: Supplementary file 1 [file Datasheet1.docx]

**Burden of gastroesophageal reflux disease among women of childbearing age, with projections to 2050: an analysis of the global burden of disease study 2021**

Siyu Zhou^1, #^, Yanping Wang^2, #^, Nengyi Hou^3, #^, Kun Hu^3^, Shun Jiang^1^, Junzhao You^3^, Hongtao Tang^4^, Jie Zeng^3^, Minghui Pang^1, 3, *^

^1^Department of Gastrointestinal Surgery, the Affiliated Hospital, Southwest Medical University, Luzhou, China

^2^Department of Gastroenterology, Union Hospital, Tongji Medical College, Huazhong University of Science and Technology, Wuhan, China

^3^Department of Geriatric General Surgery, Sichuan Provincial People's Hospital, School of Medicine, University of Electronic Science and Technology, Chengdu, China

^4^School of Clinical Medicine, North Sichuan Medical College, Nanchong, China

^#^ These authors have contributed equally to this work.

* Corresponding authors: Minghui Pang, E-mail: mhpang2025@163.com


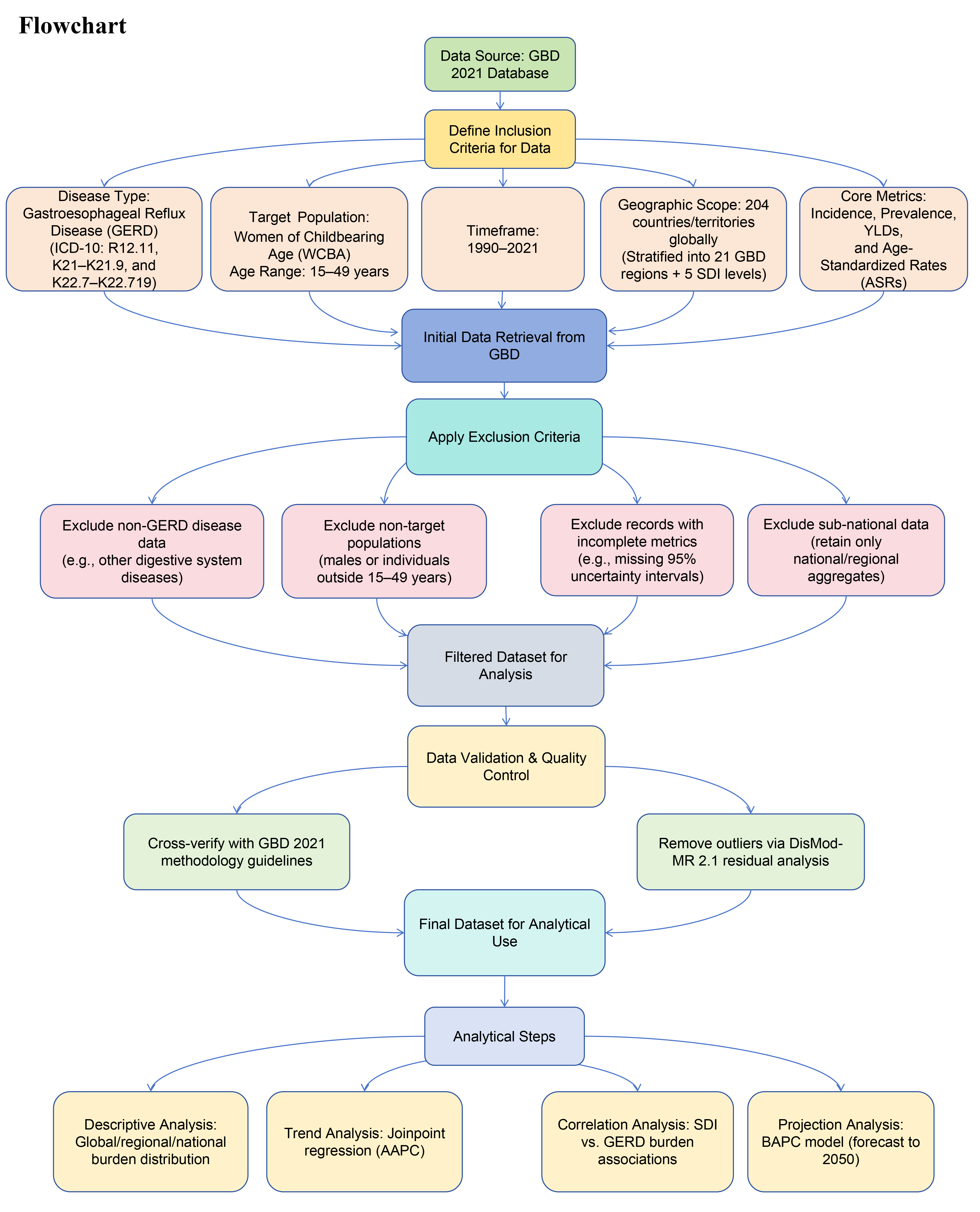


**Figure S1. Flowchart for Data Extraction, Processing and Analysis of Global Gastroesophageal Reflux Disease Burden Among Women of Childbearing Age Using GBD 2021 Database.**


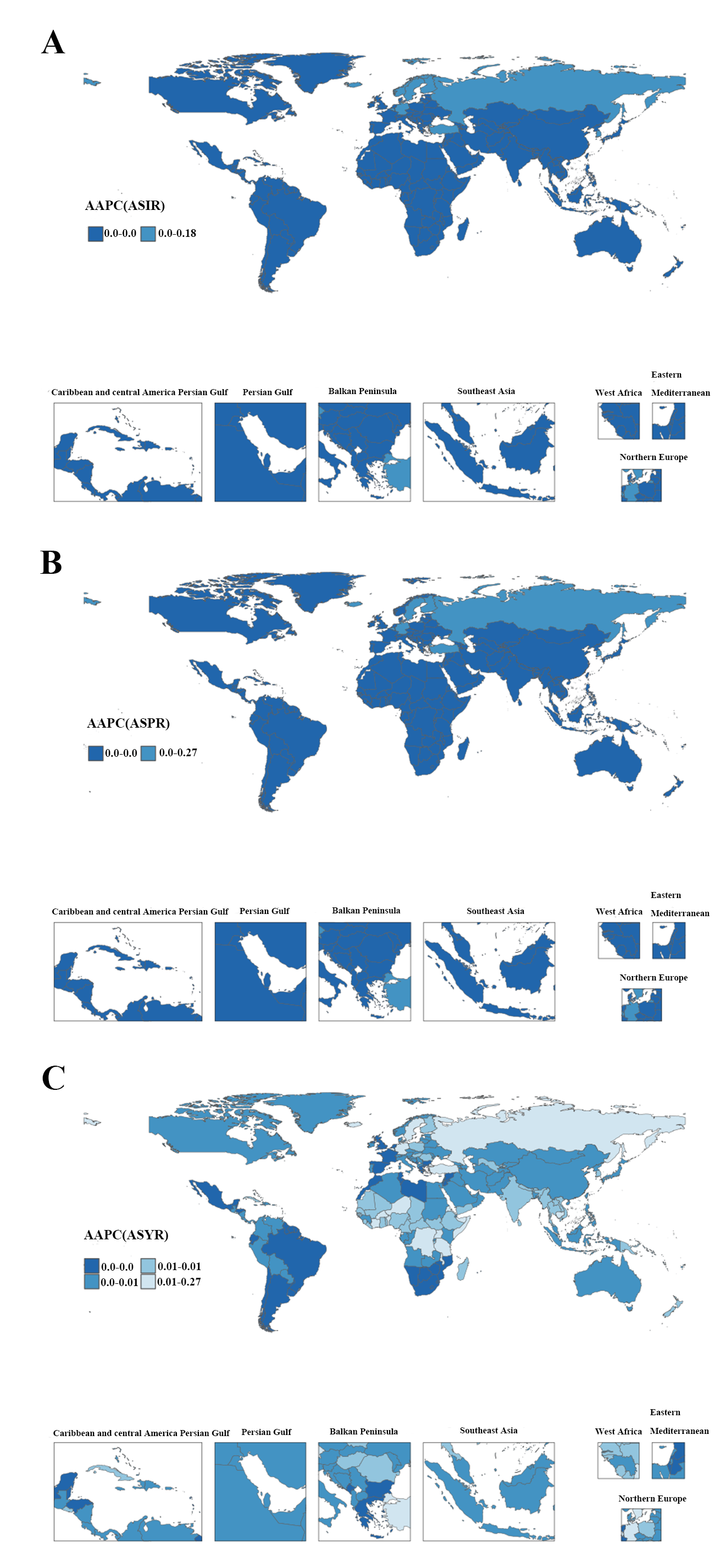


**Figure S2. The AAPC of ASIR, ASPR, and ASYR of gastroesophageal reflux disease in women of childbearing age among countries and territories in 2021.**
A. ASIR; B. ASPR; C. ASYR
AAPC, average annual percentage change; ASIR, age-standardized incidence rate; ASPR, age-standardized prevalence rate; ASYR, age-standardized YLD rate; YLDs, years lived with disability.

| Table S1. The incidence and age-standardized incidence rates of gastroesophageal reflux disease in women of childbearing age in 1990 and 2021, and estimated average annual percentage changes from 1990 to 2021 | | | | | | | |
| --- | --- | --- | --- | --- | --- | --- | --- |
|  | 1990 | |  | 2021 | |  | 1990 to 2021 |
| Location | Number of cases | ASIR per 100,000 people (95% UI) |  | Number of cases | ASIR per 100,000 people (95% UI) |  | AAPC (95% CI) |
| Andean Latin America | 715729 (513366,933457) | 7953.65 (5710.25,10303.75) |  | 1397122 (1003454,1809769) | 30956482 |  | -0.00 (-0.01 - 0.00) |
| Australasia | 244184 (170379,333059) | 4489.6 (3132.09,6125.73) |  | 339650 (236544,463400) | 4488.41 (3131.14,6124.09) |  | -0.00 (-0.04 - 0.03) |
| Caribbean | 715034 (512850,930968) | 7953.65 (5710.25,10303.75) |  | 966143 (693752,1250615) | 7953.65 (5710.25,10303.75) |  | -0.00 (-0.01 - 0.00) |
| Central Asia | 835558 (584829,1140768) | 5245.87 (3660.51,7175.37) |  | 1308922 (914154,1787758) | 5245.87 (3660.51,7175.37) |  | 0.00 (-0.00 - 0.00) |
| Central Europe | 1678143 (1178215,2262839) | 5345.9 (3752.9,7209.12) |  | 1512111 (1060221,2037907) | 5404.66 (3798.43,7285.56) |  | 0.05 (0.04 - 0.05) |
| Central Latin America | 3184575 (2307292,4153895) | 8046.28 (5835.32,10411.15) |  | 5526437 (4008120,7144127) | 8048.06 (5837.2,10411.08) |  | -0.00 (-0.01 - -0.00) |
| Central Sub-Saharan Africa | 627432 (434419,853879) | 5512.52 (3810.61,7510.54) |  | 1672178 (1157188,2277085) | 5512.52 (3810.61,7510.54) |  | 0.02 (0.01 - 0.03) |
| East Asia | 7524564 (5141046,10499459) | 2337.99 (1591.02,3267.2) |  | 8178900 (5530542,11441718) | 2322.89 (1578.78,3246.17) |  | -0.00 (-0.04 - 0.03) |
| Eastern Europe | 3289519 (2311520,4451663) | 5777.36 (4057.86,7823.1) |  | 3056648 (2144768,4137903) | 5789.7 (4069.54,7839.02) |  | 0.01 (-0.03 - 0.04) |
| Eastern Sub-Saharan Africa | 2190908 (1532686,2975448) | 5596.98 (3905.67,7595) |  | 5516634 (3857890,7492583) | 5597.74 (3906.58,7595.68) |  | 0.01 (0.01 - 0.02) |
| High-income Asia Pacific | 1252703 (857378,1726598) | 2698.18 (1850.8,3713.38) |  | 1136132 (774697,1562083) | 2746.01 (1882.64,3769.52) |  | 0.07 (0.02 - 0.12) |
| High-income North America | 4046289 (2810118,5473635) | 5231.31 (3629.47,7086.76) |  | 4089396 (2832059,5566626) | 4683.66 (3247.04,6375.72) |  | -0.47 (-0.60 - -0.33) |
| North Africa and Middle East | 4734360 (3342041,6393943) | 6447.92 (4545.09,8695.22) |  | 10414807 (7435936,13964348) | 6493.59 (4635.57,8707.87) |  | 0.03 (0.02 - 0.04) |
| Oceania | 39664 (27177,54685) | 2695.47 (1843.92,3724.76) |  | 91898 (62917,126828) | 2695.47 (1843.92,3724.76) |  | 0.01 (0.01 - 0.01) |
| South Asia | 15257490 (10730738,20521203) | 6348.46 (4455.3,8523.64) |  | 30956482 (21751940,41542386) | 6355.11 (4461.08,8525.07) |  | 0.01 (0.00 - 0.02) |
| Southeast Asia | 3139449 (2171798,4312981) | 2744.82 (1893.38,3780.15) |  | 5115982 (3527334,7048375) | 2748.05 (1896.09,3784.84) |  | 0.01 (0.01 - 0.01) |
| Southern Latin America | 764988 (533860,1041087) | 6251.28 (4360.79,8506.27) |  | 1115953 (777827,1517868) | 6251.28 (4360.79,8506.27) |  | -0.01 (-0.03 - 0.02) |
| Southern Sub-Saharan Africa | 696622 (488366,941519) | 5677.56 (3968.52,7674.71) |  | 1231670 (861864,1664056) | 5677.98 (3968.22,7677.41) |  | -0.02 (-0.03 - -0.02) |
| Tropical Latin America | 3216871 (2350777,4118209) | 8330.99 (6097.58,10612.37) |  | 5174366 (3793774,6577215) | 8287.96 (6065.62,10575.44) |  | -0.03 (-0.05 - -0.02) |
| Western Europe | 3992986 (2776078,5447142) | 4082.09 (2839.54,5572.17) |  | 4066576 (2830779,5566566) | 4099.92 (2863.76,5608.29) |  | 0.02 (0.01 - 0.02) |
| Western Sub-Saharan Africa | 2225819 (1559359,3023471) | 5610.27 (3919.97,7615.26) |  | 6195149 (4339521,8414777) | 5615.66 (3925.52,7620.34) |  | 0.02 (0.01 - 0.02) |
| ASIR, age-standardized incidence rate; UI, uncertainty interval; AAPC, average annual percentage change; CI, confidence interval. | | | | | | | |

| Table S2. The prevalence and age-standardized prevalence rates of gastroesophageal reflux disease in women of childbearing age in 1990 and 2021, and estimated average annual percentage changes from 1990 to 2021 | | | | | | | |
| --- | --- | --- | --- | --- | --- | --- | --- |
|  | 1990 | |  | 2021 | |  | 1990 to 2021 |
| Location | Number of cases | ASPR per 100,000 people (95% UI) |  | Number of cases | ASPR per 100,000 people (95% UI) |  | AAPC (95% CI) |
| Andean Latin America | 1818123 (1331710,2365740) | 20768.52 (15234.9,26934.84) |  | 3650896 (2677838,4736797) | 20768.52 (15234.9,26934.84) |  | 0.00 (0.00 - 0.00) |
| Australasia | 598974 (427093,814977) | 10964.4 (7822.85,14904.94) |  | 841630 (599591,1144807) | 10960.66 (7819.42,14899.85) |  | -0.00 (-0.05 - 0.04) |
| Caribbean | 1829999 (1340974,2379468) | 20768.52 (15234.9,26934.84) |  | 2532805 (1858659,3283014) | 20768.52 (15234.9,26934.84) |  | 0.00 (0.00 - 0.00) |
| Central Asia | 2007899 (1439055,2718716) | 12816.86 (9160.74,17373.36) |  | 3223633 (2304757,4371310) | 12816.86 (9160.74,17373.36) |  | 0.00 (0.00 - 0.00) |
| Central Europe | 4159814 (2990844,5589603) | 13155.87 (9467.8,17658.74) |  | 3823553 (2745355,5132256) | 13327.4 (9596.18,17879.65) |  | 0.04 (0.04 - 0.04) |
| Central Latin America | 7987561 (5879392,10374704) | 20791.64 (15318.64,26903.21) |  | 14319109 (10547410,18522824) | 20797.19 (15318.56,26912.97) |  | 0.00 (0.00 - 0.00) |
| Central Sub-Saharan Africa | 1497090 (1065874,2019763) | 13512.46 (9575.55,18277.37) |  | 4004401 (2847499,5406532) | 13512.46 (9575.55,18277.37) |  | 0.00 (-0.00 - 0.00) |
| East Asia | 18051241 (12709990,24856772) | 5686.71 (3994.76,7844.4) |  | 20277048 (14166901,28084741) | 5639.71 (3951.4,7792.01) |  | -0.01 (-0.05 - 0.02) |
| Eastern Europe | 8135960 (5879349,10998972) | 14166.99 (10231.55,19137.31) |  | 7705714 (5563755,10410701) | 14210.92 (10275.58,19170.6) |  | 0.01 (-0.03 - 0.05) |
| Eastern Sub-Saharan Africa | 5194821 (3715581,6965087) | 13701 (9759,18434.81) |  | 13146910 (9391632,17640531) | 13702.94 (9759.76,18437.62) |  | 0.00 (0.00 - 0.00) |
| High-income Asia Pacific | 2964370 (2097663,4069030) | 6353.81 (4504.49,8709.69) |  | 2743321 (1935573,3760420) | 6488.03 (4596.07,8871.45) |  | 0.07 (0.02 - 0.11) |
| High-income North America | 10359840 (7416543,13972642) | 13267.48 (9498.1,17879.76) |  | 10151145 (7191464,13879762) | 11495.97 (8150.63,15710.74) |  | -0.45 (-0.60 - -0.30) |
| North Africa and Middle East | 11661432 (8383412,15602776) | 16287.24 (11691.32,21823.15) |  | 26456432 (19501407,34877992) | 16454.98 (12132.72,21681.61) |  | 0.03 (0.03 - 0.04) |
| Oceania | 93939 (66449,129727) | 6508.19 (4593.83,9018) |  | 220454 (155746,305093) | 6508.19 (4593.83,9018) |  | 0.00 (-0.00 - 0.00) |
| South Asia | 36562738 (26383041,48658786) | 15490.23 (11163.07,20617.24) |  | 75194636 (54259710,100075430) | 15512.59 (11187.93,20644.74) |  | 0.00 (-0.00 - 0.01) |
| Southeast Asia | 7472123 (5295237,10206063) | 6651.37 (4699.36,9110.55) |  | 12462930 (8803939,17071872) | 6659.48 (4707.07,9117.58) |  | 0.00 (0.00 - 0.00) |
| Southern Latin America | 2023068 (1453986,2716244) | 16616.49 (11938.51,22309.36) |  | 2992756 (2149376,4017335) | 16616.49 (11938.51,22309.36) |  | -0.01 (-0.02 - 0.00) |
| Southern Sub-Saharan Africa | 1659394 (1189283,2234143) | 13884 (9913.26,18732.52) |  | 3009271 (2149699,4059800) | 13888.16 (9912.08,18730.95) |  | 0.00 (0.00 - 0.00) |
| Tropical Latin America | 8235934 (6073585,10671761) | 21763.04 (16051.06,28104.87) |  | 13667770 (10130070,17498629) | 21591.02 (16003.23,27685.91) |  | -0.03 (-0.04 - -0.02) |
| Western Europe | 9755019 (7006155,13141296) | 9914.58 (7121.81,13352.58) |  | 10055005 (7220048,13611139) | 9964.86 (7169,13473.86) |  | 0.02 (0.00 - 0.03) |
| Western Sub-Saharan Africa | 5279763 (3778711,7078025) | 13729.15 (9786.93,18469.63) |  | 14764207 (10556646,19806340) | 13743.09 (9797.02,18485.43) |  | 0.00 (0.00 - 0.00) |
| ASPR, age-standardized prevalence rate; UI, uncertainty interval; AAPC, average annual percentage change; CI, confidence interval. | | | | | | | |

| Table S3. The YLDs and age-standardized YLD rates of gastroesophageal reflux disease in women of childbearing age in 1990 and 2021, and estimated average annual percentage changes from 1990 to 2021 | | | | | | | |
| --- | --- | --- | --- | --- | --- | --- | --- |
|  | 1990 |  |  | 2021 | |  | 1990 to 2021 |
| Location | Number of cases | ASYR per 100,000 people (95% UI) |  | Number of cases | ASYR per 100,000 people (95% UI) |  | AAPC (95% CI) |
| Andean Latin America | 14194 (6885,26118) | 161.89 (78.95,297.07) |  | 28465 (13900,52247) | 161.91 (79.09,297.08) |  | -0.00 (-0.01 - 0.00) |
| Australasia | 4649 (2177,8832) | 85.12 (39.86,161.66) |  | 6539 (3094,12446) | 85.21 (40.21,161.98) |  | -0.00 (-0.04 - 0.03) |
| Caribbean | 14243 (6954,26190) | 161.46 (79.12,296.36) |  | 19665 (9623,35998) | 161.27 (78.85,295.27) |  | -0.00 (-0.01 - 0.00) |
| Central Asia | 15711 (7393,29751) | 100.13 (47.23,189.76) |  | 25184 (11912,47925) | 100.14 (47.32,190.4) |  | 0.00 (-0.00 - 0.00) |
| Central Europe | 32481 (15456,60971) | 102.76 (48.93,192.79) |  | 29853 (14199,56176) | 104.23 (49.4,195.25) |  | 0.05 (0.04 - 0.05) |
| Central Latin America | 62370 (30166,115518) | 162.05 (78.76,299.31) |  | 111427 (54121,206171) | 161.86 (78.59,299.55) |  | -0.00 (-0.01 - -0.00) |
| Central Sub-Saharan Africa | 11496 (5402,21367) | 103.55 (48.77,193.46) |  | 30969 (14443,58008) | 104.32 (48.73,196.08) |  | 0.02 (0.01 - 0.03) |
| East Asia | 141628 (66076,269237) | 44.56 (20.85,84.99) |  | 159111 (74634,304692) | 44.32 (20.72,84.5) |  | -0.00 (-0.04 - 0.03) |
| Eastern Europe | 63381 (29964,118773) | 110.37 (52.23,206.87) |  | 59876 (28262,112482) | 110.66 (52.19,207.37) |  | 0.01 (-0.03 - 0.04) |
| Eastern Sub-Saharan Africa | 40188 (19009,74923) | 105.77 (50.19,198.44) |  | 102098 (48226,190977) | 106.2 (50.37,199.69) |  | 0.01 (0.01 - 0.02) |
| High-income Asia Pacific | 23232 (10896,44297) | 49.82 (23.33,94.7) |  | 21482 (10097,41090) | 50.88 (23.8,96.44) |  | 0.07 (0.02 - 0.12) |
| High-income North America | 80573 (38997,151426) | 103.2 (49.91,193.95) |  | 78542 (37595,148736) | 89.01 (42.52,168.36) |  | -0.47 (-0.60 - -0.33) |
| North Africa and Middle East | 90553 (42873,168662) | 126.21 (60,235.92) |  | 204781 (98874,379270) | 127.38 (61.54,235.98) |  | 0.03 (0.02 - 0.04) |
| Oceania | 732 (344,1392) | 50.62 (23.92,96.46) |  | 1720 (802,3255) | 50.75 (23.68,96.11) |  | 0.01 (0.01 - 0.01) |
| South Asia | 282618 (135551,533675) | 119.55 (57.47,226.44) |  | 582074 (279765,1102621) | 120.01 (57.73,227.52) |  | 0.01 (0.00 - 0.02) |
| Southeast Asia | 58529 (27605,110763) | 52.03 (24.6,98.58) |  | 97600 (46130,185076) | 52.17 (24.65,98.89) |  | 0.01 (0.01 - 0.01) |
| Southern Latin America | 15751 (7486,29515) | 129.33 (61.49,242.43) |  | 23252 (11057,43715) | 129.16 (61.39,242.69) |  | -0.01 (-0.03 - 0.02) |
| Southern Sub-Saharan Africa | 12863 (6096,24031) | 107.39 (51.03,201.41) |  | 23103 (10970,43460) | 106.57 (50.62,200.62) |  | -0.02 (-0.03 - -0.02) |
| Tropical Latin America | 63871 (30867,118240) | 168.55 (81.84,312.07) |  | 105687 (51321,193112) | 167.07 (81,305.32) |  | -0.03 (-0.05 - -0.02) |
| Western Europe | 75918 (36156,143081) | 77.18 (36.73,145.34) |  | 78127 (37206,148066) | 77.51 (36.77,146.32) |  | 0.02 (0.01 - 0.02) |
| Western Sub-Saharan Africa | 40828 (19333,76217) | 105.92 (50.35,198.88) |  | 114602 (54140,213837) | 106.48 (50.46,199.6) |  | 0.02 (0.01 - 0.02) |
| YLDs, years lived with disability; ASYR, age-standardized YLD rate; UI, uncertainty interval; AAPC, average annual percentage change; CI, confidence interval. | | | | | | | |

| Table S4. The incidence and age-standardized incidence rates of gastroesophageal reflux disease in women of childbearing age in 1990 and 2021, and estimated average annual percentage changes from 1990 to 2021 | | | | | | | |
| --- | --- | --- | --- | --- | --- | --- | --- |
|  | 1990 | |  | 2021 | |  | 1990 to 2021 |
| Location | Number of cases | ASIR per 100,000 people (95% UI) |  | Number of cases | ASIR per 100,000 people (95% UI) |  | AAPC (95% CI) |
| American Samoa | 308 (211,425) | 2695.47 (1843.92,3724.76) |  | 312 (213,433) | 2695.47 (1843.92,3724.76) |  | 0.00 (0.00 - 0.00) |
| Antigua and Barbuda | 1270 (913,1651) | 7953.65 (5710.25,10303.75) |  | 1985 (1425,2563) | 7953.65 (5710.25,10303.75) |  | -0.00 (-0.00 - 0.00) |
| Arab Republic of Egypt | 805026 (564745,1087436) | 6412.25 (4494.31,8653.8) |  | 1640621 (1151299,2214399) | 6412.25 (4494.31,8653.8) |  | -0.00 (-0.00 - -0.00) |
| Argentine Republic | 496884 (346549,676495) | 6251.28 (4360.79,8506.27) |  | 757799 (528242,1031044) | 6251.28 (4360.79,8506.27) |  | -0.01 (-0.02 - 0.00) |
| Australia | 200574 (139544,274544) | 4427 (3080.39,6062.36) |  | 280031 (194544,383502) | 4427 (3080.39,6062.36) |  | 0.00 (-0.01 - 0.01) |
| Barbados | 5435 (3909,7049) | 7953.65 (5710.25,10303.75) |  | 5863 (4207,7562) | 7953.65 (5710.25,10303.75) |  | -0.00 (-0.00 - 0.00) |
| Belize | 3087 (2212,4041) | 7953.65 (5710.25,10303.75) |  | 9372 (6725,12179) | 7953.65 (5710.25,10303.75) |  | -0.00 (-0.00 - 0.00) |
| Bermuda | 1418 (1020,1830) | 7953.65 (5710.25,10303.75) |  | 1138 (817,1463) | 7953.65 (5710.25,10303.75) |  | -0.00 (-0.00 - 0.00) |
| Bolivarian Republic of Venezuela | 369754 (265567,481639) | 7953.65 (5710.25,10303.75) |  | 558440 (401132,720593) | 7953.65 (5710.25,10303.75) |  | -0.00 (-0.00 - 0.00) |
| Bosnia and Herzegovina | 56675 (39408,77643) | 4872.99 (3385.36,6682.06) |  | 38209 (26494,52464) | 4872.99 (3385.36,6682.06) |  | 0.00 (0.00 - 0.00) |
| Brunei Darussalam | 1961 (1358,2701) | 3011.23 (2074.55,4151.77) |  | 3897 (2685,5370) | 3011.23 (2074.55,4151.77) |  | 0.00 (0.00 - 0.00) |
| Burkina Faso | 106910 (73975,145573) | 5512.52 (3810.61,7510.54) |  | 279019 (193221,379794) | 5512.52 (3810.61,7510.54) |  | 0.00 (0.00 - 0.00) |
| Canada | 284845 (196058,390791) | 3713.93 (2555.54,5099.78) |  | 327272 (224614,449724) | 3713.93 (2555.54,5099.78) |  | 0.00 (-0.00 - 0.00) |
| Central African Republic | 32799 (22703,44629) | 5512.52 (3810.61,7510.54) |  | 71057 (49177,96761) | 5512.52 (3810.61,7510.54) |  | 0.00 (0.00 - 0.00) |
| Commonwealth of Dominica | 1279 (916,1668) | 7953.65 (5710.25,10303.75) |  | 1308 (939,1694) | 7953.65 (5710.25,10303.75) |  | -0.00 (-0.00 - 0.00) |
| Commonwealth of the Bahamas | 5610 (4029,7302) | 7953.65 (5710.25,10303.75) |  | 8675 (6224,11219) | 7953.65 (5710.25,10303.75) |  | -0.00 (-0.00 - 0.00) |
| Cook Islands | 118 (81,163) | 2695.47 (1843.92,3724.76) |  | 116 (80,161) | 2695.47 (1843.92,3724.76) |  | 0.00 (0.00 - 0.00) |
| Czech Republic | 129568 (89834,178149) | 4872.99 (3385.36,6682.06) |  | 123213 (85306,169216) | 4872.99 (3385.36,6682.06) |  | 0.00 (0.00 - 0.00) |
| Democratic People's Republic of Korea | 133835 (91460,184394) | 2407.97 (1643.19,3325.27) |  | 162565 (110666,224585) | 2407.97 (1643.19,3325.27) |  | -0.00 (-0.00 - -0.00) |
| Democratic Republic of Sao Tome and Principe | 1268 (878,1727) | 5512.52 (3810.61,7510.54) |  | 2929 (2026,3991) | 5512.52 (3810.61,7510.54) |  | 0.00 (0.00 - 0.00) |
| Democratic Republic of the Congo | 433013 (299807,589302) | 5512.52 (3810.61,7510.54) |  | 1086875 (752223,1479917) | 5512.52 (3810.61,7510.54) |  | 0.00 (0.00 - 0.00) |
| Democratic Republic of Timor-Leste | 4838 (3316,6664) | 2695.47 (1843.92,3724.76) |  | 8684 (5949,11977) | 2695.47 (1843.92,3724.76) |  | 0.00 (0.00 - 0.00) |
| Democratic Socialist Republic of Sri Lanka | 120841 (82753,166737) | 2695.47 (1843.92,3724.76) |  | 154632 (105689,213996) | 2695.47 (1843.92,3724.76) |  | 0.00 (0.00 - 0.00) |
| Dominican Republic | 141334 (101368,184600) | 7953.65 (5710.25,10303.75) |  | 229987 (165126,298207) | 7953.65 (5710.25,10303.75) |  | -0.00 (-0.00 - 0.00) |
| Eastern Republic of Uruguay | 46928 (32723,63863) | 6251.28 (4360.79,8506.27) |  | 53377 (37165,72658) | 6251.28 (4360.79,8506.27) |  | -0.00 (-0.00 - -0.00) |
| Federal Democratic Republic of Ethiopia | 592866 (413872,806161) | 5733.57 (3990.59,7795.67) |  | 1456806 (1016730,1981981) | 5733.57 (3990.59,7795.67) |  | 0.00 (0.00 - 0.00) |
| Federal Democratic Republic of Nepal | 270836 (190093,366088) | 6319.42 (4428.63,8530.32) |  | 556983 (390917,752073) | 6319.42 (4428.63,8530.32) |  | 0.00 (0.00 - 0.00) |
| Federal Republic of Germany | 748298 (518022,1030798) | 3694.1 (2557.38,5095.22) |  | 682559 (473324,940613) | 3754.38 (2610.02,5170.53) |  | 0.05 (0.04 - 0.06) |
| Federal Republic of Nigeria | 1035760 (722886,1409096) | 5733.57 (3990.59,7795.67) |  | 2991132 (2085544,4070979) | 5733.57 (3990.59,7795.67) |  | 0.00 (0.00 - 0.00) |
| Federal Republic of Somalia | 87896 (60836,119770) | 5512.52 (3810.61,7510.54) |  | 243725 (168584,332062) | 5512.52 (3810.61,7510.54) |  | 0.00 (0.00 - 0.00) |
| Federated States of Micronesia | 580 (398,801) | 2695.47 (1843.92,3724.76) |  | 682 (467,942) | 2695.47 (1843.92,3724.76) |  | 0.00 (0.00 - 0.00) |
| Federative Republic of Brazil | 3143826 (2297365,4024782) | 8335.79 (6101.82,10619.3) |  | 5021312 (3680269,6378053) | 8292.86 (6066.62,10575.88) |  | -0.02 (-0.04 - 0.00) |
| French Republic | 515942 (354590,711906) | 3501.68 (2405.84,4830.41) |  | 519552 (355859,717284) | 3501.68 (2405.84,4830.41) |  | -0.01 (-0.06 - 0.04) |
| Gabonese Republic | 11082 (7677,15069) | 5512.52 (3810.61,7510.54) |  | 25639 (17739,34907) | 5512.52 (3810.61,7510.54) |  | 0.00 (0.00 - 0.00) |
| Georgia | 72483 (50649,98973) | 5245.87 (3660.51,7175.37) |  | 44240 (30846,60474) | 5245.87 (3660.51,7175.37) |  | 0.00 (0.00 - 0.00) |
| Grand Duchy of Luxembourg | 4038 (2797,5617) | 3971.9 (2750.77,5528.94) |  | 6652 (4593,9269) | 3971.9 (2750.77,5528.94) |  | 0.00 (0.00 - 0.00) |
| Greenland | 611 (422,835) | 4121.36 (2836.33,5657.11) |  | 535 (369,734) | 4121.36 (2836.33,5657.11) |  | 0.00 (0.00 - 0.00) |
| Grenada | 1466 (1052,1912) | 7953.65 (5710.25,10303.75) |  | 2048 (1468,2654) | 7953.65 (5710.25,10303.75) |  | -0.00 (-0.00 - 0.00) |
| Guam | 926 (634,1275) | 2695.47 (1843.92,3724.76) |  | 982 (671,1357) | 2695.47 (1843.92,3724.76) |  | 0.00 (0.00 - 0.00) |
| Hashemite Kingdom of Jordan | 48529 (34053,65648) | 6412.25 (4494.31,8653.8) |  | 193013 (135295,260752) | 6412.25 (4494.31,8653.8) |  | -0.00 (-0.00 - -0.00) |
| Hellenic Republic | 126231 (86455,173966) | 4930.75 (3379.29,6792.9) |  | 115487 (78817,159555) | 4930.75 (3379.29,6792.9) |  | 0.00 (0.00 - 0.00) |
| Hungary | 144742 (100439,197640) | 5494.41 (3820.5,7500.65) |  | 127849 (88281,174778) | 5494.41 (3820.5,7500.65) |  | 0.00 (0.00 - 0.00) |
| Independent State of Papua New Guinea | 24557 (16827,33852) | 2695.47 (1843.92,3724.76) |  | 69235 (47408,95522) | 2695.47 (1843.92,3724.76) |  | 0.00 (0.00 - 0.00) |
| Independent State of Samoa | 914 (626,1261) | 2695.47 (1843.92,3724.76) |  | 1260 (862,1739) | 2695.47 (1843.92,3724.76) |  | 0.00 (0.00 - 0.00) |
| Ireland | 34506 (23899,48057) | 3971.9 (2750.77,5528.94) |  | 49118 (33883,68591) | 3971.9 (2750.77,5528.94) |  | 0.00 (0.00 - 0.00) |
| Islamic Republic of Afghanistan | 127861 (89284,173512) | 6412.25 (4494.31,8653.8) |  | 426992 (299132,577114) | 6412.25 (4494.31,8653.8) |  | -0.00 (-0.00 - -0.00) |
| Islamic Republic of Iran | 710898 (497147,967037) | 6106.19 (4263.9,8291.64) |  | 1507946 (1053648,2043787) | 6110.87 (4268.24,8293.08) |  | 0.00 (-0.02 - 0.02) |
| Islamic Republic of Mauritania | 23950 (16586,32596) | 5512.52 (3810.61,7510.54) |  | 54858 (37958,74704) | 5512.52 (3810.61,7510.54) |  | 0.00 (0.00 - 0.00) |
| Islamic Republic of Pakistan | 1430167 (1000427,1927041) | 6585.7 (4610.86,8839.59) |  | 3842978 (2691664,5166945) | 6585.7 (4610.86,8839.59) |  | -0.00 (-0.00 - -0.00) |
| Jamaica | 44765 (32108,58453) | 7953.65 (5710.25,10303.75) |  | 62017 (44553,80338) | 7953.65 (5710.25,10303.75) |  | -0.00 (-0.00 - 0.00) |
| Japan | 808257 (549837,1123602) | 2431.03 (1661.73,3367.8) |  | 658248 (446804,915035) | 2430.31 (1661.15,3366.96) |  | -0.00 (-0.02 - 0.01) |
| Kingdom of Bahrain | 7230 (5094,9731) | 6412.25 (4494.31,8653.8) |  | 21340 (14960,28782) | 6412.25 (4494.31,8653.8) |  | -0.00 (-0.00 - -0.00) |
| Kingdom of Belgium | 105574 (72674,144811) | 4214.73 (2901.33,5785.48) |  | 111799 (77233,154359) | 4275.65 (2958.59,5891.6) |  | 0.05 (0.04 - 0.05) |
| Kingdom of Bhutan | 8125 (5705,10997) | 6319.42 (4428.63,8530.32) |  | 12996 (9126,17538) | 6319.42 (4428.63,8530.32) |  | 0.00 (0.00 - 0.00) |
| Kingdom of Cambodia | 64062 (43893,88318) | 2695.47 (1843.92,3724.76) |  | 121393 (83122,167500) | 2695.47 (1843.92,3724.76) |  | 0.00 (0.00 - 0.00) |
| Kingdom of Denmark | 63355 (43498,87522) | 4693.76 (3231.16,6470.33) |  | 61642 (42306,85107) | 4693.76 (3231.16,6470.33) |  | -0.00 (-0.01 - 0.01) |
| Kingdom of Eswatini | 9717 (6726,13226) | 5512.52 (3810.61,7510.54) |  | 16672 (11547,22686) | 5512.52 (3810.61,7510.54) |  | 0.00 (0.00 - 0.00) |
| Kingdom of Lesotho | 19723 (13639,26871) | 5512.52 (3810.61,7510.54) |  | 26414 (18286,35953) | 5512.52 (3810.61,7510.54) |  | 0.00 (0.00 - 0.00) |
| Kingdom of Morocco | 383141 (268977,517396) | 6412.25 (4494.31,8653.8) |  | 626854 (439312,845868) | 6412.25 (4494.31,8653.8) |  | -0.00 (-0.00 - -0.00) |
| Kingdom of Norway | 23215 (15778,32340) | 2147.28 (1460.54,2989.93) |  | 27500 (18593,38347) | 2151.27 (1457.92,2998.37) |  | 0.01 (0.01 - 0.01) |
| Kingdom of Saudi Arabia | 194960 (136902,263429) | 6412.25 (4494.31,8653.8) |  | 680468 (477793,916789) | 6412.25 (4494.31,8653.8) |  | -0.00 (-0.00 - -0.00) |
| Kingdom of Spain | 354825 (242392,487894) | 3698.41 (2527.66,5085.79) |  | 400671 (272159,552725) | 3698.41 (2527.66,5085.79) |  | -0.00 (-0.02 - 0.02) |
| Kingdom of Sweden | 73820 (50351,102078) | 3441.89 (2352.93,4752.81) |  | 83609 (57423,115454) | 3595.18 (2474.43,4962.47) |  | 0.14 (0.13 - 0.15) |
| Kingdom of Thailand | 418917 (286984,577555) | 2695.47 (1843.92,3724.76) |  | 463993 (316753,642998) | 2695.47 (1843.92,3724.76) |  | 0.00 (0.00 - 0.00) |
| Kingdom of the Netherlands | 125742 (85991,174397) | 3094.88 (2117.48,4293.4) |  | 118585 (81026,164293) | 3094.88 (2117.48,4293.4) |  | 0.00 (-0.00 - 0.00) |
| Kingdom of Tonga | 559 (383,773) | 2695.47 (1843.92,3724.76) |  | 660 (452,913) | 2695.47 (1843.92,3724.76) |  | 0.00 (0.00 - 0.00) |
| Kyrgyz Republic | 51663 (36163,70547) | 5245.87 (3660.51,7175.37) |  | 90631 (63320,123795) | 5245.87 (3660.51,7175.37) |  | 0.00 (0.00 - 0.00) |
| Lao People's Democratic Republic | 24539 (16807,33852) | 2695.47 (1843.92,3724.76) |  | 52687 (36079,72679) | 2695.47 (1843.92,3724.76) |  | 0.00 (0.00 - 0.00) |
| Lebanese Republic | 46776 (32813,63163) | 6412.25 (4494.31,8653.8) |  | 99062 (69495,133472) | 6412.25 (4494.31,8653.8) |  | -0.00 (-0.00 - -0.00) |
| Malaysia | 116359 (79724,160443) | 2695.47 (1843.92,3724.76) |  | 228026 (156116,314653) | 2695.47 (1843.92,3724.76) |  | 0.00 (0.00 - 0.00) |
| Mongolia | 24479 (17117,33471) | 5245.87 (3660.51,7175.37) |  | 46082 (32174,62953) | 5245.87 (3660.51,7175.37) |  | 0.00 (0.00 - 0.00) |
| Montenegro | 7599 (5281,10418) | 4872.99 (3385.36,6682.06) |  | 7405 (5137,10165) | 4872.99 (3385.36,6682.06) |  | 0.00 (0.00 - 0.00) |
| New Zealand | 43610 (30303,59406) | 4805.87 (3338.17,6547.77) |  | 59619 (41378,81144) | 4805.87 (3338.17,6547.77) |  | 0.00 (-0.00 - 0.00) |
| North Macedonia | 24865 (17275,34099) | 4872.99 (3385.36,6682.06) |  | 27807 (19295,38140) | 4872.99 (3385.36,6682.06) |  | 0.00 (0.00 - 0.00) |
| Northern Mariana Islands | 365 (250,502) | 2695.47 (1843.92,3724.76) |  | 316 (216,439) | 2695.47 (1843.92,3724.76) |  | 0.00 (0.00 - 0.00) |
| Palestine | 26002 (18247,35149) | 6412.25 (4494.31,8653.8) |  | 80057 (56188,108078) | 6412.25 (4494.31,8653.8) |  | -0.00 (-0.00 - -0.00) |
| People's Democratic Republic of Algeria | 345230 (242448,466413) | 6412.25 (4494.31,8653.8) |  | 740891 (519612,999139) | 6412.25 (4494.31,8653.8) |  | -0.00 (-0.00 - -0.00) |
| People's Republic of Bangladesh | 1465966 (1032270,1958329) | 6553.5 (4616.19,8728.25) |  | 2978273 (2097998,3967070) | 6553.5 (4616.19,8728.25) |  | -0.01 (-0.05 - 0.04) |
| People's Republic of China | 7250188 (4949115,10117862) | 2332.91 (1586.05,3260.51) |  | 7861242 (5314944,10999812) | 2316.14 (1573.61,3237.22) |  | -0.02 (-0.06 - 0.01) |
| Plurinational State of Bolivia | 115811 (83079,150929) | 7953.65 (5710.25,10303.75) |  | 246495 (176998,319744) | 7953.65 (5710.25,10303.75) |  | -0.00 (-0.00 - 0.00) |
| Portuguese Republic | 113246 (77423,157749) | 4461.08 (3051.2,6212.63) |  | 112098 (76560,156150) | 4461.08 (3051.2,6212.63) |  | 0.00 (0.00 - 0.00) |
| Principality of Andorra | 610 (422,847) | 3971.9 (2750.77,5528.94) |  | 877 (603,1225) | 3971.9 (2750.77,5528.94) |  | 0.00 (0.00 - 0.00) |
| Principality of Monaco | 301 (208,420) | 3971.9 (2750.77,5528.94) |  | 305 (210,425) | 3971.9 (2750.77,5528.94) |  | 0.00 (0.00 - 0.00) |
| Puerto Rico | 75933 (54498,98386) | 7953.65 (5710.25,10303.75) |  | 61486 (44099,79344) | 7953.65 (5710.25,10303.75) |  | -0.00 (-0.00 - 0.00) |
| Republic of Albania | 26473 (18255,36205) | 3360.94 (2312.66,4611.16) |  | 21305 (14650,29216) | 3360.94 (2312.66,4611.16) |  | -0.00 (-0.00 - -0.00) |
| Republic of Angola | 117405 (81290,159783) | 5512.52 (3810.61,7510.54) |  | 393164 (272023,535482) | 5512.52 (3810.61,7510.54) |  | 0.00 (0.00 - 0.00) |
| Republic of Armenia | 44727 (31327,60983) | 5245.87 (3660.51,7175.37) |  | 41442 (28943,56571) | 5245.87 (3660.51,7175.37) |  | 0.00 (0.00 - 0.00) |
| Republic of Austria | 89377 (60094,125158) | 4401.37 (2961.04,6163.26) |  | 92880 (62346,130099) | 4401.37 (2961.04,6163.26) |  | 0.00 (0.00 - 0.00) |
| Republic of Azerbaijan | 93397 (65421,127394) | 5245.87 (3660.51,7175.37) |  | 151086 (105557,206181) | 5245.87 (3660.51,7175.37) |  | 0.00 (0.00 - 0.00) |
| Republic of Belarus | 145211 (101107,198599) | 5628.33 (3912.91,7706.52) |  | 129975 (90189,178037) | 5628.33 (3912.91,7706.52) |  | -0.00 (-0.00 - -0.00) |
| Republic of Benin | 56172 (38915,76388) | 5512.52 (3810.61,7510.54) |  | 165027 (114219,224644) | 5512.52 (3810.61,7510.54) |  | 0.00 (0.00 - 0.00) |
| Republic of Botswana | 16087 (11141,21894) | 5512.52 (3810.61,7510.54) |  | 37677 (26075,51309) | 5512.52 (3810.61,7510.54) |  | 0.00 (0.00 - 0.00) |
| Republic of Bulgaria | 104530 (72513,143593) | 4872.99 (3385.36,6682.06) |  | 76136 (52748,104525) | 4872.99 (3385.36,6682.06) |  | 0.00 (0.00 - 0.00) |
| Republic of Burundi | 64475 (44661,87716) | 5512.52 (3810.61,7510.54) |  | 158444 (109773,215682) | 5512.52 (3810.61,7510.54) |  | 0.00 (0.00 - 0.00) |
| Republic of Cabo Verde | 3948 (2736,5369) | 5512.52 (3810.61,7510.54) |  | 8234 (5702,11208) | 5512.52 (3810.61,7510.54) |  | 0.00 (0.00 - 0.00) |
| Republic of Cameroon | 120554 (83460,164051) | 5512.52 (3810.61,7510.54) |  | 402739 (278869,548226) | 5512.52 (3810.61,7510.54) |  | 0.00 (0.00 - 0.00) |
| Republic of Chad | 67611 (46807,92045) | 5512.52 (3810.61,7510.54) |  | 191854 (132795,261258) | 5512.52 (3810.61,7510.54) |  | 0.00 (0.00 - 0.00) |
| Republic of Chile | 221139 (154562,300681) | 6251.28 (4360.79,8506.27) |  | 304715 (212378,414082) | 6251.28 (4360.79,8506.27) |  | -0.00 (-0.00 - -0.00) |
| Republic of Colombia | 663914 (476598,865136) | 7953.65 (5710.25,10303.75) |  | 1055062 (757658,1365830) | 7953.65 (5710.25,10303.75) |  | -0.00 (-0.00 - 0.00) |
| Republic of Costa Rica | 59988 (43093,78099) | 7953.65 (5710.25,10303.75) |  | 105551 (75899,136418) | 7953.65 (5710.25,10303.75) |  | -0.00 (-0.00 - 0.00) |
| Republic of Croatia | 60567 (42069,83047) | 4872.99 (3385.36,6682.06) |  | 46936 (32556,64417) | 4872.99 (3385.36,6682.06) |  | 0.00 (0.00 - 0.00) |
| Republic of Cuba | 238024 (170568,309505) | 7953.65 (5710.25,10303.75) |  | 206483 (148125,266298) | 7953.65 (5710.25,10303.75) |  | -0.00 (-0.00 - 0.00) |
| Republic of Cyprus | 7961 (5513,11078) | 3971.9 (2750.77,5528.94) |  | 15534 (10734,21654) | 3971.9 (2750.77,5528.94) |  | 0.00 (0.00 - 0.00) |
| Republic of C么te d'Ivoire | 137707 (95389,187282) | 5512.52 (3810.61,7510.54) |  | 348566 (241410,474462) | 5512.52 (3810.61,7510.54) |  | 0.00 (0.00 - 0.00) |
| Republic of Djibouti | 4911 (3399,6687) | 5512.52 (3810.61,7510.54) |  | 17825 (12337,24279) | 5512.52 (3810.61,7510.54) |  | 0.00 (0.00 - 0.00) |
| Republic of Ecuador | 190146 (136398,248027) | 7953.65 (5710.25,10303.75) |  | 374643 (268978,485702) | 7953.65 (5710.25,10303.75) |  | -0.00 (-0.00 - 0.00) |
| Republic of El Salvador | 97650 (69976,127534) | 7953.65 (5710.25,10303.75) |  | 141359 (101401,183292) | 7953.65 (5710.25,10303.75) |  | -0.00 (-0.00 - 0.00) |
| Republic of Equatorial Guinea | 5058 (3502,6884) | 5512.52 (3810.61,7510.54) |  | 18927 (13106,25763) | 5512.52 (3810.61,7510.54) |  | 0.00 (0.00 - 0.00) |
| Republic of Estonia | 22182 (15415,30370) | 5628.33 (3912.91,7706.52) |  | 16864 (11696,23099) | 5628.33 (3912.91,7706.52) |  | -0.00 (-0.00 - -0.00) |
| Republic of Fiji | 5093 (3488,7025) | 2695.47 (1843.92,3724.76) |  | 6162 (4216,8517) | 2695.47 (1843.92,3724.76) |  | 0.00 (0.00 - 0.00) |
| Republic of Finland | 65397 (45061,90131) | 4904.24 (3382.39,6755.79) |  | 58583 (40198,80167) | 4920.61 (3387,6732.43) |  | 0.01 (0.01 - 0.01) |
| Republic of Ghana | 180613 (125073,245754) | 5512.52 (3810.61,7510.54) |  | 485757 (336243,661165) | 5512.52 (3810.61,7510.54) |  | 0.00 (0.00 - 0.00) |
| Republic of Guatemala | 136644 (97960,178413) | 7953.65 (5710.25,10303.75) |  | 338907 (243135,440938) | 7953.65 (5710.25,10303.75) |  | -0.00 (-0.00 - 0.00) |
| Republic of Guinea | 71702 (49626,97621) | 5512.52 (3810.61,7510.54) |  | 170492 (118064,232062) | 5512.52 (3810.61,7510.54) |  | 0.00 (0.00 - 0.00) |
| Republic of Guinea-Bissau | 11839 (8197,16116) | 5512.52 (3810.61,7510.54) |  | 27247 (18871,37086) | 5512.52 (3810.61,7510.54) |  | 0.00 (0.00 - 0.00) |
| Republic of Guyana | 15363 (11022,20054) | 7953.65 (5710.25,10303.75) |  | 16027 (11490,20807) | 7953.65 (5710.25,10303.75) |  | -0.00 (-0.00 - 0.00) |
| Republic of Haiti | 116835 (83874,152263) | 7953.65 (5710.25,10303.75) |  | 278582 (200235,361415) | 7953.65 (5710.25,10303.75) |  | -0.00 (-0.00 - 0.00) |
| Republic of Honduras | 78201 (56061,102164) | 7953.65 (5710.25,10303.75) |  | 219559 (157566,285597) | 7953.65 (5710.25,10303.75) |  | -0.00 (-0.00 - 0.00) |
| Republic of Iceland | 2224 (1521,3060) | 3435.91 (2347.54,4730.95) |  | 2877 (1963,3946) | 3476.48 (2375.44,4770.16) |  | 0.04 (0.04 - 0.04) |
| Republic of India | 12082395 (8495410,16290762) | 6295.48 (4418.91,8480) |  | 23565252 (16553672,31745151) | 6295.48 (4418.91,8480) |  | -0.00 (-0.00 - -0.00) |
| Republic of Indonesia | 1269314 (876391,1752280) | 2789.74 (1920.25,3859.78) |  | 2142608 (1473161,2965934) | 2789.74 (1920.25,3859.78) |  | 0.00 (0.00 - 0.00) |
| Republic of Iraq | 241025 (169171,325813) | 6412.25 (4494.31,8653.8) |  | 658660 (461817,889187) | 6412.25 (4494.31,8653.8) |  | -0.00 (-0.00 - -0.00) |
| Republic of Italy | 686631 (478249,939671) | 4726.97 (3295.53,6470.75) |  | 623796 (432519,854037) | 4728.65 (3295.84,6474.07) |  | 0.00 (-0.02 - 0.02) |
| Republic of Kazakhstan | 211924 (148227,289308) | 5245.87 (3660.51,7175.37) |  | 259404 (181022,354421) | 5245.87 (3660.51,7175.37) |  | 0.00 (0.00 - 0.00) |
| Republic of Kenya | 261369 (182464,355688) | 5733.57 (3990.59,7795.67) |  | 705400 (492084,959572) | 5733.57 (3990.59,7795.67) |  | 0.00 (0.00 - 0.00) |
| Republic of Kiribati | 484 (332,667) | 2695.47 (1843.92,3724.76) |  | 848 (580,1170) | 2695.47 (1843.92,3724.76) |  | 0.00 (0.00 - 0.00) |
| Republic of Korea | 410578 (281359,565481) | 3329.31 (2274.35,4591.04) |  | 418984 (284719,577895) | 3329.31 (2274.35,4591.04) |  | 0.01 (-0.07 - 0.08) |
| Republic of Latvia | 37746 (26207,51661) | 5628.33 (3912.91,7706.52) |  | 23714 (16433,32486) | 5628.33 (3912.91,7706.52) |  | -0.00 (-0.00 - -0.00) |
| Republic of Liberia | 28290 (19602,38471) | 5512.52 (3810.61,7510.54) |  | 72653 (50265,98962) | 5512.52 (3810.61,7510.54) |  | 0.00 (0.00 - 0.00) |
| Republic of Lithuania | 53935 (37557,73526) | 5739.05 (3998.83,7827.77) |  | 35134 (24423,47919) | 5739.05 (3998.83,7827.77) |  | -0.00 (-0.00 - -0.00) |
| Republic of Madagascar | 135288 (93681,184135) | 5512.52 (3810.61,7510.54) |  | 370491 (256380,504450) | 5512.52 (3810.61,7510.54) |  | 0.00 (0.00 - 0.00) |
| Republic of Malawi | 113270 (78431,154120) | 5512.52 (3810.61,7510.54) |  | 249503 (172761,339778) | 5512.52 (3810.61,7510.54) |  | 0.00 (0.00 - 0.00) |
| Republic of Maldives | 1172 (803,1614) | 2695.47 (1843.92,3724.76) |  | 3231 (2212,4462) | 2695.47 (1843.92,3724.76) |  | 0.00 (0.00 - 0.00) |
| Republic of Mali | 98930 (68478,134669) | 5512.52 (3810.61,7510.54) |  | 271556 (188002,369678) | 5512.52 (3810.61,7510.54) |  | 0.00 (0.00 - 0.00) |
| Republic of Malta | 3931 (2717,5479) | 3971.9 (2750.77,5528.94) |  | 4045 (2792,5638) | 3971.9 (2750.77,5528.94) |  | 0.00 (0.00 - 0.00) |
| Republic of Mauritius | 7938 (5440,10941) | 2695.47 (1843.92,3724.76) |  | 8765 (5989,12127) | 2695.47 (1843.92,3724.76) |  | 0.00 (0.00 - 0.00) |
| Republic of Moldova | 64343 (44802,87996) | 5628.33 (3912.91,7706.52) |  | 54130 (37582,74113) | 5628.33 (3912.91,7706.52) |  | -0.00 (-0.00 - -0.00) |
| Republic of Mozambique | 161916 (112037,220562) | 5512.52 (3810.61,7510.54) |  | 380025 (263007,517298) | 5512.52 (3810.61,7510.54) |  | 0.00 (0.00 - 0.00) |
| Republic of Namibia | 16948 (11734,23067) | 5512.52 (3810.61,7510.54) |  | 35269 (24405,48015) | 5512.52 (3810.61,7510.54) |  | 0.00 (0.00 - 0.00) |
| Republic of Nauru | 63 (43,87) | 2695.47 (1843.92,3724.76) |  | 74 (51,103) | 2695.47 (1843.92,3724.76) |  | 0.00 (0.00 - 0.00) |
| Republic of Nicaragua | 66482 (47660,86954) | 7953.65 (5710.25,10303.75) |  | 143585 (103139,186206) | 7953.65 (5710.25,10303.75) |  | -0.00 (-0.00 - 0.00) |
| Republic of Niue | 13 (9,18) | 2695.47 (1843.92,3724.76) |  | 10 (7,14) | 2695.47 (1843.92,3724.76) |  | 0.00 (0.00 - 0.00) |
| Republic of Palau | 108 (74,149) | 2695.47 (1843.92,3724.76) |  | 105 (72,146) | 2695.47 (1843.92,3724.76) |  | 0.00 (0.00 - 0.00) |
| Republic of Panama | 46652 (33469,60786) | 7953.65 (5710.25,10303.75) |  | 85118 (61083,110291) | 7953.65 (5710.25,10303.75) |  | -0.00 (-0.00 - 0.00) |
| Republic of Paraguay | 73045 (52864,95377) | 8124.4 (5887.48,10542.4) |  | 153054 (110981,198889) | 8124.4 (5887.48,10542.4) |  | 0.00 (0.00 - 0.00) |
| Republic of Peru | 409772 (293889,534501) | 7953.65 (5710.25,10303.75) |  | 775984 (557477,1004323) | 7953.65 (5710.25,10303.75) |  | -0.00 (-0.00 - 0.00) |
| Republic of Poland | 615111 (433819,827430) | 6343.57 (4463.51,8535.21) |  | 613000 (431582,823417) | 6343.57 (4463.51,8535.21) |  | 0.00 (-0.00 - 0.00) |
| Republic of Rwanda | 81779 (56665,111277) | 5512.52 (3810.61,7510.54) |  | 183686 (127164,250134) | 5512.52 (3810.61,7510.54) |  | 0.00 (0.00 - 0.00) |
| Republic of San Marino | 248 (172,345) | 3971.9 (2750.77,5528.94) |  | 304 (209,424) | 3971.9 (2750.77,5528.94) |  | 0.00 (0.00 - 0.00) |
| Republic of Senegal | 86320 (59751,117518) | 5512.52 (3810.61,7510.54) |  | 201681 (139592,274563) | 5512.52 (3810.61,7510.54) |  | 0.00 (0.00 - 0.00) |
| Republic of Serbia | 116180 (80693,159235) | 4872.99 (3385.36,6682.06) |  | 105051 (72856,144193) | 4872.99 (3385.36,6682.06) |  | 0.00 (0.00 - 0.00) |
| Republic of Seychelles | 461 (316,634) | 2695.47 (1843.92,3724.76) |  | 673 (460,931) | 2695.47 (1843.92,3724.76) |  | 0.00 (0.00 - 0.00) |
| Republic of Sierra Leone | 51848 (35931,70501) | 5512.52 (3810.61,7510.54) |  | 116035 (80356,157902) | 5512.52 (3810.61,7510.54) |  | 0.00 (0.00 - 0.00) |
| Republic of Singapore | 31908 (21967,43834) | 3376.27 (2319.11,4647.52) |  | 55002 (37660,75922) | 3392.42 (2330.93,4678.77) |  | 0.02 (0.01 - 0.02) |
| Republic of Slovenia | 24894 (17301,34110) | 4872.99 (3385.36,6682.06) |  | 22416 (15533,30791) | 4872.99 (3385.36,6682.06) |  | 0.00 (0.00 - 0.00) |
| Republic of South Africa | 515595 (359758,701038) | 5733.57 (3990.59,7795.67) |  | 904242 (629800,1228547) | 5733.57 (3990.59,7795.67) |  | 0.00 (0.00 - 0.00) |
| Republic of South Sudan | 64329 (44540,87554) | 5512.52 (3810.61,7510.54) |  | 118187 (81755,161154) | 5512.52 (3810.61,7510.54) |  | 0.00 (0.00 - 0.00) |
| Republic of Sudan | 281605 (197676,380448) | 6412.25 (4494.31,8653.8) |  | 695576 (488330,939305) | 6412.25 (4494.31,8653.8) |  | -0.00 (-0.00 - -0.00) |
| Republic of Suriname | 7411 (5311,9658) | 7953.65 (5710.25,10303.75) |  | 11668 (8376,15096) | 7953.65 (5710.25,10303.75) |  | -0.00 (-0.00 - 0.00) |
| Republic of Tajikistan | 57267 (40088,78255) | 5245.87 (3660.51,7175.37) |  | 131303 (91828,179306) | 5245.87 (3660.51,7175.37) |  | 0.00 (0.00 - 0.00) |
| Republic of the Congo | 28075 (19440,38211) | 5512.52 (3810.61,7510.54) |  | 76517 (52920,104255) | 5512.52 (3810.61,7510.54) |  | 0.00 (0.00 - 0.00) |
| Republic of the Gambia | 11391 (7892,15492) | 5512.52 (3810.61,7510.54) |  | 31242 (21636,42523) | 5512.52 (3810.61,7510.54) |  | 0.00 (0.00 - 0.00) |
| Republic of the Marshall Islands | 244 (167,336) | 2695.47 (1843.92,3724.76) |  | 394 (269,544) | 2695.47 (1843.92,3724.76) |  | 0.00 (0.00 - 0.00) |
| Republic of the Niger | 87723 (60701,119456) | 5512.52 (3810.61,7510.54) |  | 259219 (179338,353062) | 5512.52 (3810.61,7510.54) |  | 0.00 (0.00 - 0.00) |
| Republic of the Philippines | 406440 (280759,561210) | 2789.74 (1920.25,3859.78) |  | 802159 (552725,1108657) | 2789.74 (1920.25,3859.78) |  | 0.00 (0.00 - 0.00) |
| Republic of the Union of Myanmar | 266183 (182438,366857) | 2695.47 (1843.92,3724.76) |  | 406055 (277747,561201) | 2695.47 (1843.92,3724.76) |  | 0.00 (0.00 - 0.00) |
| Republic of Trinidad and Tobago | 24034 (17261,31228) | 7953.65 (5710.25,10303.75) |  | 28109 (20224,36223) | 7953.65 (5710.25,10303.75) |  | -0.00 (-0.00 - 0.00) |
| Republic of Tunisia | 124365 (87332,167899) | 6412.25 (4494.31,8653.8) |  | 204814 (143479,276297) | 6412.25 (4494.31,8653.8) |  | -0.00 (-0.00 - -0.00) |
| Republic of Turkey | 935552 (656240,1268637) | 6874.17 (4822.17,9300.1) |  | 1617735 (1187166,2140181) | 7293.19 (5358.86,9648.4) |  | 0.18 (0.15 - 0.22) |
| Republic of Uganda | 188773 (130713,256828) | 5512.52 (3810.61,7510.54) |  | 519198 (359459,706714) | 5512.52 (3810.61,7510.54) |  | 0.00 (0.00 - 0.00) |
| Republic of Uzbekistan | 236698 (165783,323231) | 5245.87 (3660.51,7175.37) |  | 478991 (334578,654160) | 5245.87 (3660.51,7175.37) |  | 0.00 (0.00 - 0.00) |
| Republic of Vanuatu | 892 (612,1230) | 2695.47 (1843.92,3724.76) |  | 2049 (1403,2828) | 2695.47 (1843.92,3724.76) |  | 0.00 (0.00 - 0.00) |
| Republic of Yemen | 165238 (116048,223178) | 6412.25 (4494.31,8653.8) |  | 513023 (360106,693091) | 6412.25 (4494.31,8653.8) |  | -0.00 (-0.00 - -0.00) |
| Republic of Zambia | 89347 (61838,121632) | 5512.52 (3810.61,7510.54) |  | 249678 (172886,339812) | 5512.52 (3810.61,7510.54) |  | 0.00 (0.00 - 0.00) |
| Republic of Zimbabwe | 118551 (82102,161368) | 5512.52 (3810.61,7510.54) |  | 211397 (146318,287868) | 5512.52 (3810.61,7510.54) |  | 0.00 (0.00 - 0.00) |
| Romania | 274296 (190642,376385) | 4872.99 (3385.36,6682.06) |  | 213443 (147840,293274) | 4872.99 (3385.36,6682.06) |  | 0.00 (0.00 - 0.00) |
| Russian Federation | 2198924 (1530457,2984120) | 5763.89 (4009.34,7827.36) |  | 2142937 (1490000,2907435) | 5782.86 (4028.09,7848.82) |  | 0.01 (-0.03 - 0.05) |
| Saint Kitts and Nevis | 755 (543,985) | 7953.65 (5710.25,10303.75) |  | 1279 (919,1652) | 7953.65 (5710.25,10303.75) |  | -0.00 (-0.00 - 0.00) |
| Saint Lucia | 2548 (1827,3328) | 7953.65 (5710.25,10303.75) |  | 3759 (2698,4851) | 7953.65 (5710.25,10303.75) |  | -0.00 (-0.00 - 0.00) |
| Saint Vincent and the Grenadines | 1950 (1398,2550) | 7953.65 (5710.25,10303.75) |  | 2242 (1609,2899) | 7953.65 (5710.25,10303.75) |  | -0.00 (-0.00 - 0.00) |
| Slovak Republic | 65807 (45706,90273) | 4872.99 (3385.36,6682.06) |  | 67333 (46675,92439) | 4872.99 (3385.36,6682.06) |  | 0.00 (0.00 - 0.00) |
| Socialist Republic of Viet Nam | 433843 (297541,597208) | 2695.47 (1843.92,3724.76) |  | 715939 (489446,989878) | 2695.47 (1843.92,3724.76) |  | 0.00 (0.00 - 0.00) |
| Solomon Islands | 1834 (1256,2529) | 2695.47 (1843.92,3724.76) |  | 4481 (3067,6188) | 2695.47 (1843.92,3724.76) |  | 0.00 (0.00 - 0.00) |
| State of Eritrea | 40111 (27764,54600) | 5512.52 (3810.61,7510.54) |  | 86963 (60180,118398) | 5512.52 (3810.61,7510.54) |  | 0.00 (0.00 - 0.00) |
| State of Israel | 48780 (33773,66557) | 4072.59 (2816.92,5556.26) |  | 91738 (63366,125248) | 4072.59 (2816.92,5556.26) |  | -0.00 (-0.01 - 0.01) |
| State of Kuwait | 26058 (18352,35103) | 6412.25 (4494.31,8653.8) |  | 101464 (71212,136649) | 6412.25 (4494.31,8653.8) |  | -0.00 (-0.00 - -0.00) |
| State of Libya | 52810 (37047,71456) | 6412.25 (4494.31,8653.8) |  | 130457 (91379,176056) | 6412.25 (4494.31,8653.8) |  | -0.00 (-0.00 - -0.00) |
| State of Qatar | 5100 (3590,6872) | 6412.25 (4494.31,8653.8) |  | 38129 (26812,51310) | 6412.25 (4494.31,8653.8) |  | -0.00 (-0.00 - -0.00) |
| Sultanate of Oman | 20669 (14516,27904) | 6412.25 (4494.31,8653.8) |  | 67231 (47247,90585) | 6412.25 (4494.31,8653.8) |  | -0.00 (-0.00 - -0.00) |
| Swiss Confederation | 48977 (33315,67865) | 2651.15 (1806.52,3675.05) |  | 56048 (38073,77751) | 2651.15 (1806.52,3675.05) |  | 0.00 (-0.01 - 0.02) |
| Syrian Arab Republic | 162012 (113692,219065) | 6412.25 (4494.31,8653.8) |  | 240035 (167708,325168) | 6412.25 (4494.31,8653.8) |  | -0.00 (-0.00 - -0.00) |
| Taiwan (Province of China) | 140541 (97395,193934) | 2565.44 (1774.32,3549.57) |  | 155093 (106158,215430) | 2593.2 (1784.22,3591.24) |  | 0.03 (0.01 - 0.05) |
| Togolese Republic | 43207 (29914,58805) | 5512.52 (3810.61,7510.54) |  | 114837 (79473,156391) | 5512.52 (3810.61,7510.54) |  | 0.00 (0.00 - 0.00) |
| Tokelau | 9 (6,13) | 2695.47 (1843.92,3724.76) |  | 9 (6,12) | 2695.47 (1843.92,3724.76) |  | 0.00 (0.00 - 0.00) |
| Turkmenistan | 42919 (30053,58608) | 5245.87 (3660.51,7175.37) |  | 65743 (45886,89896) | 5245.87 (3660.51,7175.37) |  | 0.00 (0.00 - 0.00) |
| Tuvalu | 65 (45,90) | 2695.47 (1843.92,3724.76) |  | 76 (52,105) | 2695.47 (1843.92,3724.76) |  | 0.00 (0.00 - 0.00) |
| Ukraine | 767177 (536648,1047269) | 5873.74 (4106.16,8026.4) |  | 653894 (457044,892357) | 5873.74 (4106.16,8026.4) |  | 0.00 (0.00 - 0.00) |
| Union of the Comoros | 5275 (3651,7183) | 5512.52 (3810.61,7510.54) |  | 10452 (7228,14234) | 5512.52 (3810.61,7510.54) |  | 0.00 (0.00 - 0.00) |
| United Arab Emirates | 21683 (15283,29189) | 6412.25 (4494.31,8653.8) |  | 120727 (84548,163321) | 6412.25 (4494.31,8653.8) |  | -0.00 (-0.00 - -0.00) |
| United Kingdom of Great Britain and Northern Ireland | 746476 (519572,1017209) | 5116.68 (3565.01,6970.74) |  | 826738 (574946,1131399) | 5116.85 (3566.14,7003.66) |  | -0.00 (-0.02 - 0.02) |
| United Mexican States | 1665289 (1200448,2175718) | 8136.91 (5883.6,10540) |  | 2878858 (2081977,3724630) | 8136.91 (5883.6,10540) |  | 0.00 (0.00 - 0.00) |
| United Republic of Tanzania | 297736 (206159,405158) | 5512.52 (3810.61,7510.54) |  | 761449 (526903,1036742) | 5512.52 (3810.61,7510.54) |  | 0.00 (0.00 - 0.00) |
| United States of America | 3760741 (2605277,5079302) | 5399.36 (3736.88,7303.96) |  | 3761524 (2600882,5127865) | 4792.32 (3316.86,6533.07) |  | -0.37 (-0.46 - -0.28) |
| United States Virgin Islands | 2306 (1654,2980) | 7953.65 (5710.25,10303.75) |  | 1419 (1017,1829) | 7953.65 (5710.25,10303.75) |  | -0.00 (-0.00 - 0.00) |
| ASIR, age-standardized incidence rate; UI, uncertainty interval; AAPC, average annual percentage change; CI, confidence interval. | | | | | | | |

| Table S5. The prevalence and age-standardized prevalence rates of gastroesophageal reflux disease in women of childbearing age in 1990 and 2021, and estimated average annual percentage changes from 1990 to 2021 | | | | | | | |
| --- | --- | --- | --- | --- | --- | --- | --- |
|  | 1990 | |  | 2021 | |  | 1990 to 2021 |
| Location | Number of cases | ASPR per 100,000 people (95% UI) |  | Number of cases | ASPR per 100,000 people (95% UI) |  | AAPC (95% CI) |
| American Samoa | 731 (518,1010) | 6508.19 (4593.83,9018) |  | 752 (530,1042) | 6508.19 (4593.83,9018) |  | 0.00 (-0.00 - 0.00) |
| Antigua and Barbuda | 3274 (2399,4257) | 20768.52 (15234.9,26934.84) |  | 5264 (3865,6812) | 20768.52 (15234.9,26934.84) |  | 0.00 (0.00 - 0.00) |
| Arab Republic of Egypt | 1990996 (1422787,2678765) | 16135.88 (11519.36,21724.55) |  | 4104946 (2931832,5527048) | 16135.88 (11519.36,21724.55) |  | -0.00 (-0.00 - -0.00) |
| Argentine Republic | 1316253 (945568,1767382) | 16616.49 (11938.51,22309.36) |  | 2029094 (1457080,2724212) | 16616.49 (11938.51,22309.36) |  | -0.02 (-0.04 - 0.01) |
| Australia | 491849 (349190,672001) | 10802.04 (7674.15,14746.16) |  | 693691 (491974,947859) | 10802.04 (7674.15,14746.16) |  | -0.00 (-0.05 - 0.04) |
| Barbados | 14123 (10356,18349) | 20768.52 (15234.9,26934.84) |  | 15588 (11449,20155) | 20768.52 (15234.9,26934.84) |  | 0.00 (0.00 - 0.00) |
| Belize | 7723 (5653,10066) | 20768.52 (15234.9,26934.84) |  | 24160 (17711,31377) | 20768.52 (15234.9,26934.84) |  | 0.00 (0.00 - 0.00) |
| Bermuda | 3758 (2758,4873) | 20768.52 (15234.9,26934.84) |  | 3067 (2253,3959) | 20768.52 (15234.9,26934.84) |  | 0.00 (0.00 - 0.00) |
| Bolivarian Republic of Venezuela | 943947 (691479,1228549) | 20768.52 (15234.9,26934.84) |  | 1479767 (1086327,1915309) | 20768.52 (15234.9,26934.84) |  | 0.00 (0.00 - 0.00) |
| Bosnia and Herzegovina | 136094 (96858,185600) | 11712.94 (8329.79,15974.89) |  | 93618 (66412,127872) | 11712.94 (8329.79,15974.89) |  | 0.00 (0.00 - 0.00) |
| Brunei Darussalam | 4520 (3218,6198) | 7017.41 (4979.54,9636.69) |  | 9165 (6502,12593) | 7017.41 (4979.54,9636.69) |  | 0.00 (0.00 - 0.00) |
| Burkina Faso | 255792 (181949,345274) | 13512.46 (9575.55,18277.37) |  | 666009 (474019,898869) | 13512.46 (9575.55,18277.37) |  | 0.00 (-0.00 - 0.00) |
| Canada | 669080 (475264,911308) | 8640.28 (6136.99,11763.53) |  | 773094 (547667,1054355) | 8640.28 (6136.99,11763.53) |  | 0.00 (-0.00 - 0.00) |
| Central African Republic | 78413 (55815,105782) | 13512.46 (9575.55,18277.37) |  | 170289 (121045,229942) | 13512.46 (9575.55,18277.37) |  | 0.00 (-0.00 - 0.00) |
| Commonwealth of Dominica | 3243 (2376,4218) | 20768.52 (15234.9,26934.84) |  | 3418 (2508,4432) | 20768.52 (15234.9,26934.84) |  | 0.00 (0.00 - 0.00) |
| Commonwealth of the Bahamas | 14392 (10548,18719) | 20768.52 (15234.9,26934.84) |  | 22817 (16747,29550) | 20768.52 (15234.9,26934.84) |  | 0.00 (0.00 - 0.00) |
| Cook Islands | 281 (199,388) | 6508.19 (4593.83,9018) |  | 282 (199,390) | 6508.19 (4593.83,9018) |  | 0.00 (-0.00 - 0.00) |
| Czech Republic | 314798 (223344,430367) | 11712.94 (8329.79,15974.89) |  | 304604 (215821,416171) | 11712.94 (8329.79,15974.89) |  | 0.00 (0.00 - 0.00) |
| Democratic People's Republic of Korea | 320482 (226099,442117) | 5801.41 (4085.65,8022.07) |  | 394973 (277878,546768) | 5801.41 (4085.65,8022.07) |  | 0.00 (0.00 - 0.00) |
| Democratic Republic of Sao Tome and Principe | 3002 (2139,4048) | 13512.46 (9575.55,18277.37) |  | 7064 (5016,9545) | 13512.46 (9575.55,18277.37) |  | 0.00 (-0.00 - 0.00) |
| Democratic Republic of the Congo | 1033046 (735476,1393724) | 13512.46 (9575.55,18277.37) |  | 2599111 (1848677,3508626) | 13512.46 (9575.55,18277.37) |  | 0.00 (-0.00 - 0.00) |
| Democratic Republic of Timor-Leste | 11519 (8149,15908) | 6508.19 (4593.83,9018) |  | 20446 (14468,28198) | 6508.19 (4593.83,9018) |  | 0.00 (-0.00 - 0.00) |
| Democratic Socialist Republic of Sri Lanka | 289287 (204409,400339) | 6508.19 (4593.83,9018) |  | 375508 (264860,521116) | 6508.19 (4593.83,9018) |  | 0.00 (-0.00 - 0.00) |
| Dominican Republic | 356937 (261349,464863) | 20768.52 (15234.9,26934.84) |  | 598737 (439116,777010) | 20768.52 (15234.9,26934.84) |  | 0.00 (0.00 - 0.00) |
| Eastern Republic of Uruguay | 124753 (89625,167471) | 16616.49 (11938.51,22309.36) |  | 143263 (102835,192313) | 16616.49 (11938.51,22309.36) |  | -0.00 (-0.00 - -0.00) |
| Federal Democratic Republic of Ethiopia | 1406833 (1007410,1900959) | 14007.65 (9996.12,18965.36) |  | 3459249 (2474891,4677690) | 14007.65 (9996.12,18965.36) |  | 0.00 (0.00 - 0.00) |
| Federal Democratic Republic of Nepal | 655490 (469120,882082) | 15605.04 (11150.58,20990.69) |  | 1362427 (974457,1833162) | 15605.04 (11150.58,20990.69) |  | 0.00 (0.00 - 0.00) |
| Federal Republic of Germany | 1767577 (1255811,2409352) | 8646.91 (6142.7,11790.25) |  | 1634232 (1149998,2247339) | 8842.03 (6235.3,12137.9) |  | 0.07 (0.06 - 0.08) |
| Federal Republic of Nigeria | 2437504 (1745643,3291607) | 14007.65 (9996.12,18965.36) |  | 7091725 (5072803,9584177) | 14007.65 (9996.12,18965.36) |  | 0.00 (0.00 - 0.00) |
| Federal Republic of Somalia | 211710 (150387,286147) | 13512.46 (9575.55,18277.37) |  | 580748 (412812,784179) | 13512.46 (9575.55,18277.37) |  | 0.00 (-0.00 - 0.00) |
| Federated States of Micronesia | 1366 (966,1886) | 6508.19 (4593.83,9018) |  | 1629 (1151,2252) | 6508.19 (4593.83,9018) |  | 0.00 (-0.00 - 0.00) |
| Federative Republic of Brazil | 8047893 (5934927,10428588) | 21769.99 (16056.9,28114.26) |  | 13265923 (9830192,16981391) | 21594 (16001.78,27686.18) |  | -0.03 (-0.04 - -0.02) |
| French Republic | 1194722 (844416,1639652) | 8070.67 (5708.54,11060.55) |  | 1210687 (854660,1661721) | 8070.67 (5708.54,11060.55) |  | -0.01 (-0.08 - 0.05) |
| Gabonese Republic | 26302 (18756,35444) | 13512.46 (9575.55,18277.37) |  | 61808 (43922,83471) | 13512.46 (9575.55,18277.37) |  | 0.00 (-0.00 - 0.00) |
| Georgia | 177331 (126904,240311) | 12816.86 (9160.74,17373.36) |  | 110260 (78721,149609) | 12816.86 (9160.74,17373.36) |  | 0.00 (0.00 - 0.00) |
| Grand Duchy of Luxembourg | 9763 (6906,13382) | 9498.74 (6720.73,13016.58) |  | 16202 (11435,22217) | 9498.74 (6720.73,13016.58) |  | 0.00 (0.00 - 0.00) |
| Greenland | 1466 (1040,1995) | 9958.36 (7038.65,13581.58) |  | 1299 (919,1773) | 9958.36 (7038.65,13581.58) |  | 0.00 (0.00 - 0.00) |
| Grenada | 3720 (2725,4842) | 20768.52 (15234.9,26934.84) |  | 5348 (3925,6932) | 20768.52 (15234.9,26934.84) |  | 0.00 (0.00 - 0.00) |
| Guam | 2220 (1569,3072) | 6508.19 (4593.83,9018) |  | 2378 (1679,3294) | 6508.19 (4593.83,9018) |  | 0.00 (-0.00 - 0.00) |
| Hashemite Kingdom of Jordan | 117298 (83889,157638) | 16135.88 (11519.36,21724.55) |  | 480223 (342922,646349) | 16135.88 (11519.36,21724.55) |  | -0.00 (-0.00 - -0.00) |
| Hellenic Republic | 333336 (234019,458746) | 12954.81 (9095.2,17819.85) |  | 311373 (218541,429745) | 12954.81 (9095.2,17819.85) |  | 0.00 (0.00 - 0.00) |
| Hungary | 371647 (265880,503339) | 13917.41 (9967.4,18822.4) |  | 332614 (237876,450383) | 13917.41 (9967.4,18822.4) |  | -0.00 (-0.00 - -0.00) |
| Independent State of Papua New Guinea | 58121 (41117,80249) | 6508.19 (4593.83,9018) |  | 166037 (117316,229749) | 6508.19 (4593.83,9018) |  | 0.00 (-0.00 - 0.00) |
| Independent State of Samoa | 2143 (1516,2956) | 6508.19 (4593.83,9018) |  | 3001 (2121,4148) | 6508.19 (4593.83,9018) |  | 0.00 (-0.00 - 0.00) |
| Ireland | 82223 (58171,112730) | 9498.74 (6720.73,13016.58) |  | 119291 (84057,163721) | 9498.74 (6720.73,13016.58) |  | 0.00 (0.00 - 0.00) |
| Islamic Republic of Afghanistan | 309383 (220703,416239) | 16135.88 (11519.36,21724.55) |  | 1041157 (744738,1397889) | 16135.88 (11519.36,21724.55) |  | -0.00 (-0.00 - -0.00) |
| Islamic Republic of Iran | 1686787 (1213568,2266370) | 14938.71 (10718.12,20126.6) |  | 3764813 (2699807,5081873) | 14952.8 (10737.08,20128.23) |  | 0.00 (-0.02 - 0.02) |
| Islamic Republic of Mauritania | 57218 (40727,77217) | 13512.46 (9575.55,18277.37) |  | 131279 (93352,177233) | 13512.46 (9575.55,18277.37) |  | 0.00 (-0.00 - 0.00) |
| Islamic Republic of Pakistan | 3412616 (2449140,4552316) | 16114.05 (11567.31,21487.01) |  | 9290004 (6670586,12394361) | 16114.05 (11567.31,21487.01) |  | 0.00 (0.00 - 0.00) |
| Jamaica | 113248 (82943,147468) | 20768.52 (15234.9,26934.84) |  | 162134 (118945,210390) | 20768.52 (15234.9,26934.84) |  | 0.00 (0.00 - 0.00) |
| Japan | 1901038 (1339263,2639330) | 5654.76 (3995.69,7826.12) |  | 1566095 (1102593,2174663) | 5652.42 (3993.59,7823.07) |  | -0.00 (-0.02 - 0.01) |
| Kingdom of Bahrain | 18007 (12897,24209) | 16135.88 (11519.36,21724.55) |  | 54105 (38605,72868) | 16135.88 (11519.36,21724.55) |  | -0.00 (-0.00 - -0.00) |
| Kingdom of Belgium | 259874 (184814,357034) | 10297.82 (7329.01,14126.8) |  | 278153 (197064,378614) | 10485.19 (7444.38,14259.02) |  | 0.06 (0.05 - 0.06) |
| Kingdom of Bhutan | 19382 (13881,26085) | 15605.04 (11150.58,20990.69) |  | 32002 (22882,43074) | 15605.04 (11150.58,20990.69) |  | 0.00 (0.00 - 0.00) |
| Kingdom of Cambodia | 151840 (107402,209728) | 6508.19 (4593.83,9018) |  | 292589 (206653,405202) | 6508.19 (4593.83,9018) |  | 0.00 (-0.00 - 0.00) |
| Kingdom of Denmark | 163525 (115564,222409) | 11994.96 (8489.63,16310.41) |  | 159303 (112650,216546) | 11994.96 (8489.63,16310.41) |  | -0.00 (-0.00 - -0.00) |
| Kingdom of Eswatini | 23047 (16415,31085) | 13512.46 (9575.55,18277.37) |  | 40285 (28645,54411) | 13512.46 (9575.55,18277.37) |  | 0.00 (-0.00 - 0.00) |
| Kingdom of Lesotho | 47541 (33766,64228) | 13512.46 (9575.55,18277.37) |  | 63538 (45178,85798) | 13512.46 (9575.55,18277.37) |  | 0.00 (-0.00 - 0.00) |
| Kingdom of Morocco | 943734 (674811,1269190) | 16135.88 (11519.36,21724.55) |  | 1583022 (1129822,2131759) | 16135.88 (11519.36,21724.55) |  | -0.00 (-0.00 - -0.00) |
| Kingdom of Norway | 53289 (37172,74189) | 4902.93 (3424.93,6818.56) |  | 63452 (44409,87890) | 4903.75 (3435.79,6783.27) |  | 0.00 (0.00 - 0.00) |
| Kingdom of Saudi Arabia | 476812 (340994,641274) | 16135.88 (11519.36,21724.55) |  | 1740405 (1242237,2345842) | 16135.88 (11519.36,21724.55) |  | -0.00 (-0.00 - -0.00) |
| Kingdom of Spain | 828856 (587243,1130781) | 8649.38 (6128.96,11807.1) |  | 958456 (674546,1310533) | 8649.38 (6128.96,11807.1) |  | -0.01 (-0.03 - 0.02) |
| Kingdom of Sweden | 174288 (123725,236992) | 8040.45 (5724.54,10930.96) |  | 199572 (140045,277227) | 8461.19 (5947.54,11738.56) |  | 0.17 (0.16 - 0.18) |
| Kingdom of Thailand | 1000902 (707494,1383704) | 6508.19 (4593.83,9018) |  | 1140998 (804137,1584893) | 6508.19 (4593.83,9018) |  | 0.00 (-0.00 - 0.00) |
| Kingdom of the Netherlands | 282167 (200463,386231) | 6908.12 (4913.06,9450.04) |  | 267196 (189720,365473) | 6908.12 (4913.06,9450.04) |  | 0.00 (-0.00 - 0.00) |
| Kingdom of Tonga | 1317 (931,1818) | 6508.19 (4593.83,9018) |  | 1578 (1115,2184) | 6508.19 (4593.83,9018) |  | 0.00 (-0.00 - 0.00) |
| Kyrgyz Republic | 123791 (88717,167660) | 12816.86 (9160.74,17373.36) |  | 221498 (158456,300153) | 12816.86 (9160.74,17373.36) |  | 0.00 (0.00 - 0.00) |
| Lao People's Democratic Republic | 58008 (41026,80090) | 6508.19 (4593.83,9018) |  | 126483 (89366,175005) | 6508.19 (4593.83,9018) |  | 0.00 (-0.00 - 0.00) |
| Lebanese Republic | 116461 (83230,156675) | 16135.88 (11519.36,21724.55) |  | 253177 (180797,341095) | 16135.88 (11519.36,21724.55) |  | -0.00 (-0.00 - -0.00) |
| Malaysia | 277213 (195963,383359) | 6508.19 (4593.83,9018) |  | 550840 (388993,762952) | 6508.19 (4593.83,9018) |  | 0.00 (-0.00 - 0.00) |
| Mongolia | 58069 (41619,78534) | 12816.86 (9160.74,17373.36) |  | 113904 (81390,154498) | 12816.86 (9160.74,17373.36) |  | 0.00 (0.00 - 0.00) |
| Montenegro | 18257 (12987,24906) | 11712.94 (8329.79,15974.89) |  | 18073 (12828,24680) | 11712.94 (8329.79,15974.89) |  | 0.00 (0.00 - 0.00) |
| New Zealand | 107125 (76555,144761) | 11790.78 (8424.65,15929.39) |  | 147938 (105672,199885) | 11790.78 (8424.65,15929.39) |  | 0.00 (-0.00 - 0.00) |
| North Macedonia | 59801 (42521,81577) | 11712.94 (8329.79,15974.89) |  | 68173 (48392,93116) | 11712.94 (8329.79,15974.89) |  | 0.00 (0.00 - 0.00) |
| Northern Mariana Islands | 873 (617,1206) | 6508.19 (4593.83,9018) |  | 771 (544,1067) | 6508.19 (4593.83,9018) |  | 0.00 (-0.00 - 0.00) |
| Palestine | 63146 (45171,84852) | 16135.88 (11519.36,21724.55) |  | 198060 (141591,266352) | 16135.88 (11519.36,21724.55) |  | -0.00 (-0.00 - -0.00) |
| People's Democratic Republic of Algeria | 841633 (602058,1131314) | 16135.88 (11519.36,21724.55) |  | 1885078 (1345416,2540186) | 16135.88 (11519.36,21724.55) |  | -0.00 (-0.00 - -0.00) |
| People's Republic of Bangladesh | 3568776 (2588947,4750017) | 16488.36 (11951.01,21932.87) |  | 7461393 (5410316,9923881) | 16488.36 (11951.01,21932.87) |  | -0.01 (-0.10 - 0.08) |
| People's Republic of China | 17384272 (12224584,23971080) | 5673.75 (3979.53,7837.15) |  | 19491437 (13595210,27027515) | 5622.05 (3931.93,7777.45) |  | -0.01 (-0.05 - 0.03) |
| Plurinational State of Bolivia | 295006 (216123,383711) | 20768.52 (15234.9,26934.84) |  | 640433 (469609,831455) | 20768.52 (15234.9,26934.84) |  | 0.00 (0.00 - 0.00) |
| Portuguese Republic | 283570 (194976,397534) | 11156.9 (7674.71,15638.29) |  | 286594 (196395,402090) | 11156.9 (7674.71,15638.29) |  | 0.00 (0.00 - 0.00) |
| Principality of Andorra | 1468 (1039,2011) | 9498.74 (6720.73,13016.58) |  | 2150 (1513,2948) | 9498.74 (6720.73,13016.58) |  | 0.00 (0.00 - 0.00) |
| Principality of Monaco | 735 (518,1008) | 9498.74 (6720.73,13016.58) |  | 742 (523,1018) | 9498.74 (6720.73,13016.58) |  | 0.00 (0.00 - 0.00) |
| Puerto Rico | 198053 (145258,256866) | 20768.52 (15234.9,26934.84) |  | 163109 (119756,210947) | 20768.52 (15234.9,26934.84) |  | 0.00 (0.00 - 0.00) |
| Republic of Albania | 56768 (40570,77138) | 7312.84 (5215.1,9967.84) |  | 46642 (33239,63540) | 7312.84 (5215.1,9967.84) |  | -0.00 (-0.00 - -0.00) |
| Republic of Angola | 280633 (199780,378671) | 13512.46 (9575.55,18277.37) |  | 941896 (669685,1271837) | 13512.46 (9575.55,18277.37) |  | 0.00 (-0.00 - 0.00) |
| Republic of Armenia | 108653 (77845,147241) | 12816.86 (9160.74,17373.36) |  | 103302 (73801,140252) | 12816.86 (9160.74,17373.36) |  | 0.00 (0.00 - 0.00) |
| Republic of Austria | 223658 (149626,317193) | 10953.13 (7329.19,15545.56) |  | 235413 (157232,334221) | 10953.13 (7329.19,15545.56) |  | 0.00 (0.00 - 0.00) |
| Republic of Azerbaijan | 224148 (160739,303325) | 12816.86 (9160.74,17373.36) |  | 374310 (267650,507724) | 12816.86 (9160.74,17373.36) |  | 0.00 (0.00 - 0.00) |
| Republic of Belarus | 361456 (257112,489248) | 13927.01 (9902.52,18837.94) |  | 330054 (234040,446203) | 13927.01 (9902.52,18837.94) |  | 0.00 (-0.00 - 0.00) |
| Republic of Benin | 134290 (95680,181116) | 13512.46 (9575.55,18277.37) |  | 393990 (280346,531692) | 13512.46 (9575.55,18277.37) |  | 0.00 (-0.00 - 0.00) |
| Republic of Botswana | 38192 (27209,51511) | 13512.46 (9575.55,18277.37) |  | 92425 (65553,125032) | 13512.46 (9575.55,18277.37) |  | 0.00 (-0.00 - 0.00) |
| Republic of Bulgaria | 253933 (180215,346922) | 11712.94 (8329.79,15974.89) |  | 187963 (133198,256865) | 11712.94 (8329.79,15974.89) |  | 0.00 (0.00 - 0.00) |
| Republic of Burundi | 154167 (109798,208005) | 13512.46 (9575.55,18277.37) |  | 377966 (269047,510213) | 13512.46 (9575.55,18277.37) |  | 0.00 (-0.00 - 0.00) |
| Republic of Cabo Verde | 9348 (6669,12596) | 13512.46 (9575.55,18277.37) |  | 20122 (14285,27203) | 13512.46 (9575.55,18277.37) |  | 0.00 (-0.00 - 0.00) |
| Republic of Cameroon | 287350 (204600,387616) | 13512.46 (9575.55,18277.37) |  | 965158 (686633,1303030) | 13512.46 (9575.55,18277.37) |  | 0.00 (-0.00 - 0.00) |
| Republic of Chad | 161525 (114941,218007) | 13512.46 (9575.55,18277.37) |  | 454675 (323711,613432) | 13512.46 (9575.55,18277.37) |  | 0.00 (-0.00 - 0.00) |
| Republic of Chile | 581966 (418725,781261) | 16616.49 (11938.51,22309.36) |  | 820233 (589342,1100589) | 16616.49 (11938.51,22309.36) |  | -0.00 (-0.00 - -0.00) |
| Republic of Colombia | 1693529 (1240622,2204031) | 20768.52 (15234.9,26934.84) |  | 2764333 (2027871,3585233) | 20768.52 (15234.9,26934.84) |  | 0.00 (0.00 - 0.00) |
| Republic of Costa Rica | 153593 (112531,199900) | 20768.52 (15234.9,26934.84) |  | 278228 (204141,360755) | 20768.52 (15234.9,26934.84) |  | 0.00 (0.00 - 0.00) |
| Republic of Croatia | 146980 (104442,200667) | 11712.94 (8329.79,15974.89) |  | 115045 (81635,157140) | 11712.94 (8329.79,15974.89) |  | 0.00 (-0.00 - 0.00) |
| Republic of Cuba | 612666 (449078,795960) | 20768.52 (15234.9,26934.84) |  | 550530 (404730,711112) | 20768.52 (15234.9,26934.84) |  | 0.00 (0.00 - 0.00) |
| Republic of Cyprus | 19108 (13517,26185) | 9498.74 (6720.73,13016.58) |  | 38011 (26811,52175) | 9498.74 (6720.73,13016.58) |  | 0.00 (0.00 - 0.00) |
| Republic of C么te d'Ivoire | 327126 (233174,441009) | 13512.46 (9575.55,18277.37) |  | 840001 (597245,1134625) | 13512.46 (9575.55,18277.37) |  | 0.00 (0.00 - 0.00) |
| Republic of Djibouti | 11656 (8294,15728) | 13512.46 (9575.55,18277.37) |  | 43750 (31010,59202) | 13512.46 (9575.55,18277.37) |  | 0.00 (-0.00 - 0.00) |
| Republic of Ecuador | 482721 (353571,628238) | 20768.52 (15234.9,26934.84) |  | 975639 (715481,1266058) | 20768.52 (15234.9,26934.84) |  | 0.00 (0.00 - 0.00) |
| Republic of El Salvador | 246627 (180619,320968) | 20768.52 (15234.9,26934.84) |  | 368113 (269968,477624) | 20768.52 (15234.9,26934.84) |  | 0.00 (0.00 - 0.00) |
| Republic of Equatorial Guinea | 12110 (8615,16342) | 13512.46 (9575.55,18277.37) |  | 45492 (32352,61431) | 13512.46 (9575.55,18277.37) |  | 0.00 (-0.00 - 0.00) |
| Republic of Estonia | 55481 (39419,75048) | 13927.01 (9902.52,18837.94) |  | 42686 (30282,57685) | 13927.01 (9902.52,18837.94) |  | 0.00 (-0.00 - 0.00) |
| Republic of Fiji | 12150 (8589,16797) | 6508.19 (4593.83,9018) |  | 14883 (10504,20635) | 6508.19 (4593.83,9018) |  | 0.00 (-0.00 - 0.00) |
| Republic of Finland | 173553 (123551,236955) | 12793.84 (9125.31,17443.17) |  | 155375 (110214,211557) | 12852.39 (9124.41,17482.78) |  | 0.01 (0.01 - 0.02) |
| Republic of Ghana | 431956 (307523,582844) | 13512.46 (9575.55,18277.37) |  | 1176143 (835729,1588826) | 13512.46 (9575.55,18277.37) |  | 0.00 (-0.00 - 0.00) |
| Republic of Guatemala | 345316 (252912,449440) | 20768.52 (15234.9,26934.84) |  | 869554 (637100,1130185) | 20768.52 (15234.9,26934.84) |  | 0.00 (0.00 - 0.00) |
| Republic of Guinea | 173044 (122979,233749) | 13512.46 (9575.55,18277.37) |  | 408429 (290620,551359) | 13512.46 (9575.55,18277.37) |  | 0.00 (-0.00 - 0.00) |
| Republic of Guinea-Bissau | 28259 (20114,38137) | 13512.46 (9575.55,18277.37) |  | 65470 (46573,88408) | 13512.46 (9575.55,18277.37) |  | 0.00 (-0.00 - 0.00) |
| Republic of Guyana | 38898 (28487,50653) | 20768.52 (15234.9,26934.84) |  | 41537 (30456,53918) | 20768.52 (15234.9,26934.84) |  | 0.00 (0.00 - 0.00) |
| Republic of Haiti | 297725 (218136,387411) | 20768.52 (15234.9,26934.84) |  | 722834 (529933,938883) | 20768.52 (15234.9,26934.84) |  | 0.00 (0.00 - 0.00) |
| Republic of Honduras | 197178 (144405,256721) | 20768.52 (15234.9,26934.84) |  | 564028 (413326,733144) | 20768.52 (15234.9,26934.84) |  | 0.00 (0.00 - 0.00) |
| Republic of Iceland | 5080 (3607,6933) | 7856.05 (5575.02,10724.2) |  | 6671 (4749,9115) | 7987.5 (5691.34,10906.03) |  | 0.05 (0.05 - 0.06) |
| Republic of India | 28906474 (20690653,38500369) | 15298.04 (10941.47,20370.78) |  | 57048809 (40811470,75973552) | 15298.04 (10941.47,20370.78) |  | -0.00 (-0.00 - -0.00) |
| Republic of Indonesia | 3027915 (2146422,4155266) | 6780.99 (4796.84,9331.02) |  | 5241675 (3705937,7217400) | 6780.99 (4796.84,9331.02) |  | 0.00 (-0.00 - 0.00) |
| Republic of Iraq | 587915 (420393,790637) | 16135.88 (11519.36,21724.55) |  | 1640482 (1171904,2207068) | 16135.88 (11519.36,21724.55) |  | -0.00 (-0.00 - -0.00) |
| Republic of Italy | 1754953 (1259856,2359673) | 12028.86 (8637.37,16168.05) |  | 1627328 (1165894,2190570) | 12027.22 (8637.97,16169.08) |  | -0.00 (-0.03 - 0.03) |
| Republic of Kazakhstan | 514805 (368618,697739) | 12816.86 (9160.74,17373.36) |  | 642182 (458897,871119) | 12816.86 (9160.74,17373.36) |  | 0.00 (0.00 - 0.00) |
| Republic of Kenya | 612287 (438580,826700) | 14007.65 (9996.12,18965.36) |  | 1681768 (1202906,2274451) | 14007.65 (9996.12,18965.36) |  | 0.00 (0.00 - 0.00) |
| Republic of Kiribati | 1151 (815,1590) | 6508.19 (4593.83,9018) |  | 2035 (1438,2818) | 6508.19 (4593.83,9018) |  | 0.00 (-0.00 - 0.00) |
| Republic of Korea | 981592 (694871,1342853) | 8016.5 (5661.48,10990.34) |  | 1030581 (725410,1416715) | 8016.5 (5661.48,10990.34) |  | 0.01 (-0.10 - 0.11) |
| Republic of Latvia | 94404 (67099,127599) | 13927.01 (9902.52,18837.94) |  | 60120 (42647,81218) | 13927.01 (9902.52,18837.94) |  | 0.00 (-0.00 - 0.00) |
| Republic of Liberia | 67537 (48149,91080) | 13512.46 (9575.55,18277.37) |  | 174981 (124298,236401) | 13512.46 (9575.55,18277.37) |  | 0.00 (-0.00 - 0.00) |
| Republic of Lithuania | 135722 (96713,182140) | 14354.77 (10229.15,19274.47) |  | 89632 (63726,120276) | 14354.77 (10229.15,19274.47) |  | -0.00 (-0.00 - -0.00) |
| Republic of Madagascar | 321737 (229143,434028) | 13512.46 (9575.55,18277.37) |  | 886580 (630507,1196793) | 13512.46 (9575.55,18277.37) |  | 0.00 (-0.00 - 0.00) |
| Republic of Malawi | 268923 (191539,362675) | 13512.46 (9575.55,18277.37) |  | 592094 (421387,799113) | 13512.46 (9575.55,18277.37) |  | 0.00 (-0.00 - 0.00) |
| Republic of Maldives | 2739 (1940,3770) | 6508.19 (4593.83,9018) |  | 7898 (5569,10968) | 6508.19 (4593.83,9018) |  | 0.00 (-0.00 - 0.00) |
| Republic of Mali | 237246 (168740,320278) | 13512.46 (9575.55,18277.37) |  | 644341 (458825,869235) | 13512.46 (9575.55,18277.37) |  | 0.00 (-0.00 - 0.00) |
| Republic of Malta | 9511 (6712,13053) | 9498.74 (6720.73,13016.58) |  | 9864 (6958,13526) | 9498.74 (6720.73,13016.58) |  | 0.00 (0.00 - 0.00) |
| Republic of Mauritius | 19053 (13468,26362) | 6508.19 (4593.83,9018) |  | 21361 (15070,29636) | 6508.19 (4593.83,9018) |  | 0.00 (-0.00 - 0.00) |
| Republic of Moldova | 160157 (113934,216797) | 13927.01 (9902.52,18837.94) |  | 137483 (97529,185910) | 13927.01 (9902.52,18837.94) |  | 0.00 (-0.00 - 0.00) |
| Republic of Mozambique | 388276 (275976,524421) | 13512.46 (9575.55,18277.37) |  | 902531 (642575,1217370) | 13512.46 (9575.55,18277.37) |  | 0.00 (-0.00 - 0.00) |
| Republic of Namibia | 40231 (28655,54260) | 13512.46 (9575.55,18277.37) |  | 85496 (60731,115510) | 13512.46 (9575.55,18277.37) |  | 0.00 (-0.00 - 0.00) |
| Republic of Nauru | 150 (106,208) | 6508.19 (4593.83,9018) |  | 178 (126,246) | 6508.19 (4593.83,9018) |  | 0.00 (-0.00 - 0.00) |
| Republic of Nicaragua | 166850 (122141,217376) | 20768.52 (15234.9,26934.84) |  | 373397 (273807,484781) | 20768.52 (15234.9,26934.84) |  | 0.00 (0.00 - 0.00) |
| Republic of Niue | 31 (22,43) | 6508.19 (4593.83,9018) |  | 25 (18,35) | 6508.19 (4593.83,9018) |  | 0.00 (-0.00 - 0.00) |
| Republic of Palau | 257 (182,356) | 6508.19 (4593.83,9018) |  | 258 (182,358) | 6508.19 (4593.83,9018) |  | 0.00 (-0.00 - 0.00) |
| Republic of Panama | 119018 (87192,154811) | 20768.52 (15234.9,26934.84) |  | 222151 (162948,288063) | 20768.52 (15234.9,26934.84) |  | 0.00 (0.00 - 0.00) |
| Republic of Paraguay | 188040 (138183,244632) | 21456.29 (15771.15,27836.65) |  | 401847 (295363,521851) | 21456.29 (15771.15,27836.65) |  | -0.00 (-0.00 - -0.00) |
| Republic of Peru | 1040396 (762015,1353791) | 20768.52 (15234.9,26934.84) |  | 2034823 (1492748,2639284) | 20768.52 (15234.9,26934.84) |  | 0.00 (0.00 - 0.00) |
| Republic of Poland | 1574883 (1128561,2109326) | 16063.4 (11518.74,21517.58) |  | 1598503 (1144112,2138934) | 16063.4 (11518.74,21517.58) |  | 0.00 (0.00 - 0.00) |
| Republic of Rwanda | 194707 (138720,262667) | 13512.46 (9575.55,18277.37) |  | 442413 (314334,597716) | 13512.46 (9575.55,18277.37) |  | 0.00 (-0.00 - 0.00) |
| Republic of San Marino | 594 (420,814) | 9498.74 (6720.73,13016.58) |  | 739 (521,1013) | 9498.74 (6720.73,13016.58) |  | 0.00 (0.00 - 0.00) |
| Republic of Senegal | 205227 (146103,276899) | 13512.46 (9575.55,18277.37) |  | 484211 (344323,653752) | 13512.46 (9575.55,18277.37) |  | 0.00 (-0.00 - 0.00) |
| Republic of Serbia | 281031 (199771,383390) | 11712.94 (8329.79,15974.89) |  | 256809 (182260,350739) | 11712.94 (8329.79,15974.89) |  | 0.00 (0.00 - 0.00) |
| Republic of Seychelles | 1090 (772,1503) | 6508.19 (4593.83,9018) |  | 1641 (1157,2277) | 6508.19 (4593.83,9018) |  | 0.00 (-0.00 - 0.00) |
| Republic of Sierra Leone | 124172 (88454,167512) | 13512.46 (9575.55,18277.37) |  | 277129 (197258,373970) | 13512.46 (9575.55,18277.37) |  | 0.00 (-0.00 - 0.00) |
| Republic of Singapore | 77220 (54527,105925) | 8161.24 (5754.11,11202) |  | 137481 (96201,189184) | 8223.25 (5774.97,11272.89) |  | 0.03 (0.02 - 0.03) |
| Republic of Slovenia | 60248 (42846,82208) | 11712.94 (8329.79,15974.89) |  | 55262 (39166,75548) | 11712.94 (8329.79,15974.89) |  | 0.00 (0.00 - 0.00) |
| Republic of South Africa | 1230149 (880206,1663054) | 14007.65 (9996.12,18965.36) |  | 2218747 (1584289,3006208) | 14007.65 (9996.12,18965.36) |  | 0.00 (0.00 - 0.00) |
| Republic of South Sudan | 152323 (108512,205432) | 13512.46 (9575.55,18277.37) |  | 283061 (200810,382624) | 13512.46 (9575.55,18277.37) |  | 0.00 (-0.00 - 0.00) |
| Republic of Sudan | 690405 (493688,928458) | 16135.88 (11519.36,21724.55) |  | 1722577 (1231040,2318309) | 16135.88 (11519.36,21724.55) |  | -0.00 (-0.00 - -0.00) |
| Republic of Suriname | 18902 (13850,24573) | 20768.52 (15234.9,26934.84) |  | 30633 (22479,39688) | 20768.52 (15234.9,26934.84) |  | 0.00 (0.00 - 0.00) |
| Republic of Tajikistan | 135500 (97169,183314) | 12816.86 (9160.74,17373.36) |  | 319110 (228371,432401) | 12816.86 (9160.74,17373.36) |  | 0.00 (0.00 - 0.00) |
| Republic of the Congo | 66586 (47432,89800) | 13512.46 (9575.55,18277.37) |  | 185804 (131817,251225) | 13512.46 (9575.55,18277.37) |  | 0.00 (-0.00 - 0.00) |
| Republic of the Gambia | 27038 (19280,36452) | 13512.46 (9575.55,18277.37) |  | 74552 (53065,100614) | 13512.46 (9575.55,18277.37) |  | 0.00 (-0.00 - 0.00) |
| Republic of the Marshall Islands | 573 (406,792) | 6508.19 (4593.83,9018) |  | 945 (667,1310) | 6508.19 (4593.83,9018) |  | 0.00 (-0.00 - 0.00) |
| Republic of the Niger | 208964 (148687,282011) | 13512.46 (9575.55,18277.37) |  | 610301 (434713,822985) | 13512.46 (9575.55,18277.37) |  | 0.00 (-0.00 - 0.00) |
| Republic of the Philippines | 966001 (684805,1325677) | 6780.99 (4796.84,9331.02) |  | 1936009 (1370714,2661094) | 6780.99 (4796.84,9331.02) |  | 0.00 (-0.00 - 0.00) |
| Republic of the Union of Myanmar | 630376 (445915,870384) | 6508.19 (4593.83,9018) |  | 979310 (691292,1356680) | 6508.19 (4593.83,9018) |  | 0.00 (-0.00 - 0.00) |
| Republic of Trinidad and Tobago | 62050 (45491,80659) | 20768.52 (15234.9,26934.84) |  | 74871 (54967,96927) | 20768.52 (15234.9,26934.84) |  | 0.00 (0.00 - 0.00) |
| Republic of Tunisia | 306243 (218998,411793) | 16135.88 (11519.36,21724.55) |  | 523352 (373212,705414) | 16135.88 (11519.36,21724.55) |  | -0.00 (-0.00 - -0.00) |
| Republic of Turkey | 2396474 (1719643,3191816) | 17974.33 (12878.41,23935.58) |  | 4398513 (3558229,5341559) | 19614.72 (15857.84,23852.7) |  | 0.27 (0.23 - 0.32) |
| Republic of Uganda | 445022 (317370,599690) | 13512.46 (9575.55,18277.37) |  | 1231541 (877132,1661123) | 13512.46 (9575.55,18277.37) |  | 0.00 (-0.00 - 0.00) |
| Republic of Uzbekistan | 563302 (404080,762157) | 12816.86 (9160.74,17373.36) |  | 1178601 (842739,1598117) | 12816.86 (9160.74,17373.36) |  | 0.00 (0.00 - 0.00) |
| Republic of Vanuatu | 2111 (1494,2915) | 6508.19 (4593.83,9018) |  | 4895 (3459,6768) | 6508.19 (4593.83,9018) |  | 0.00 (-0.00 - 0.00) |
| Republic of Yemen | 405817 (290328,545945) | 16135.88 (11519.36,21724.55) |  | 1269271 (906963,1709099) | 16135.88 (11519.36,21724.55) |  | -0.00 (-0.00 - -0.00) |
| Republic of Zambia | 210112 (149746,283203) | 13512.46 (9575.55,18277.37) |  | 595569 (423975,803660) | 13512.46 (9575.55,18277.37) |  | 0.00 (-0.00 - 0.00) |
| Republic of Zimbabwe | 280234 (199680,377916) | 13512.46 (9575.55,18277.37) |  | 508780 (361516,687300) | 13512.46 (9575.55,18277.37) |  | 0.00 (-0.00 - 0.00) |
| Romania | 659733 (468551,900761) | 11712.94 (8329.79,15974.89) |  | 524611 (371676,716755) | 11712.94 (8329.79,15974.89) |  | 0.00 (0.00 - 0.00) |
| Russian Federation | 5426177 (3918447,7318341) | 14100.15 (10176.55,18991.44) |  | 5392723 (3888733,7268451) | 14171.92 (10233.64,19074.92) |  | 0.02 (-0.03 - 0.07) |
| Saint Kitts and Nevis | 1916 (1404,2497) | 20768.52 (15234.9,26934.84) |  | 3387 (2487,4385) | 20768.52 (15234.9,26934.84) |  | 0.00 (0.00 - 0.00) |
| Saint Lucia | 6433 (4711,8376) | 20768.52 (15234.9,26934.84) |  | 9976 (7325,12909) | 20768.52 (15234.9,26934.84) |  | 0.00 (0.00 - 0.00) |
| Saint Vincent and the Grenadines | 4901 (3589,6386) | 20768.52 (15234.9,26934.84) |  | 5898 (4328,7639) | 20768.52 (15234.9,26934.84) |  | 0.00 (0.00 - 0.00) |
| Slovak Republic | 159118 (113082,217329) | 11712.94 (8329.79,15974.89) |  | 165986 (117692,226886) | 11712.94 (8329.79,15974.89) |  | 0.00 (0.00 - 0.00) |
| Socialist Republic of Viet Nam | 1025372 (725844,1414140) | 6508.19 (4593.83,9018) |  | 1750788 (1234613,2430168) | 6508.19 (4593.83,9018) |  | 0.00 (-0.00 - 0.00) |
| Solomon Islands | 4290 (3036,5916) | 6508.19 (4593.83,9018) |  | 10706 (7563,14816) | 6508.19 (4593.83,9018) |  | 0.00 (-0.00 - 0.00) |
| State of Eritrea | 95817 (68188,129287) | 13512.46 (9575.55,18277.37) |  | 209847 (149108,283447) | 13512.46 (9575.55,18277.37) |  | 0.00 (-0.00 - 0.00) |
| State of Israel | 117234 (82867,158906) | 9829.24 (6952.96,13320.73) |  | 222207 (157119,301312) | 9829.24 (6952.96,13320.73) |  | -0.00 (-0.01 - 0.01) |
| State of Kuwait | 64968 (46487,87409) | 16135.88 (11519.36,21724.55) |  | 262804 (187452,354618) | 16135.88 (11519.36,21724.55) |  | -0.00 (-0.00 - -0.00) |
| State of Libya | 127511 (91202,171411) | 16135.88 (11519.36,21724.55) |  | 331138 (236209,446099) | 16135.88 (11519.36,21724.55) |  | -0.00 (-0.00 - -0.00) |
| State of Qatar | 12862 (9197,17337) | 16135.88 (11519.36,21724.55) |  | 98436 (70295,132823) | 16135.88 (11519.36,21724.55) |  | -0.00 (-0.00 - -0.00) |
| Sultanate of Oman | 50987 (36475,68595) | 16135.88 (11519.36,21724.55) |  | 171210 (122273,230813) | 16135.88 (11519.36,21724.55) |  | -0.00 (-0.00 - -0.00) |
| Swiss Confederation | 106996 (76308,146935) | 5737.36 (4097.21,7877.36) |  | 123249 (87824,169495) | 5737.36 (4097.21,7877.36) |  | 0.00 (-0.01 - 0.02) |
| Syrian Arab Republic | 393371 (281381,528784) | 16135.88 (11519.36,21724.55) |  | 595085 (423804,802085) | 16135.88 (11519.36,21724.55) |  | -0.00 (-0.00 - -0.00) |
| Taiwan (Province of China) | 346487 (247060,473000) | 6335.62 (4506.8,8662.69) |  | 390638 (274817,538849) | 6399.17 (4511.35,8805.34) |  | 0.02 (-0.00 - 0.05) |
| Togolese Republic | 102994 (73332,138962) | 13512.46 (9575.55,18277.37) |  | 278454 (197709,376349) | 13512.46 (9575.55,18277.37) |  | 0.00 (-0.00 - 0.00) |
| Tokelau | 22 (15,30) | 6508.19 (4593.83,9018) |  | 21 (15,29) | 6508.19 (4593.83,9018) |  | 0.00 (-0.00 - 0.00) |
| Turkmenistan | 102300 (73364,138436) | 12816.86 (9160.74,17373.36) |  | 160465 (114732,217438) | 12816.86 (9160.74,17373.36) |  | 0.00 (0.00 - 0.00) |
| Tuvalu | 158 (111,218) | 6508.19 (4593.83,9018) |  | 182 (129,252) | 6508.19 (4593.83,9018) |  | 0.00 (-0.00 - 0.00) |
| Ukraine | 1902563 (1358658,2562722) | 14429.14 (10303.56,19440.33) |  | 1653016 (1178805,2229101) | 14429.14 (10303.56,19440.33) |  | 0.00 (-0.00 - 0.00) |
| Union of the Comoros | 12540 (8926,16919) | 13512.46 (9575.55,18277.37) |  | 25379 (18013,34295) | 13512.46 (9575.55,18277.37) |  | 0.00 (-0.00 - 0.00) |
| United Arab Emirates | 54242 (38835,72995) | 16135.88 (11519.36,21724.55) |  | 313902 (223004,424987) | 16135.88 (11519.36,21724.55) |  | -0.00 (-0.00 - -0.00) |
| United Kingdom of Great Britain and Northern Ireland | 1900920 (1355507,2565244) | 12934.87 (9232.56,17445.2) |  | 2119890 (1514260,2884648) | 12922.8 (9240.42,17572.79) |  | -0.01 (-0.03 - 0.02) |
| United Mexican States | 4121502 (3016440,5378712) | 20823.28 (15250.21,27085.99) |  | 7399537 (5419473,9618302) | 20823.28 (15250.21,27085.99) |  | 0.00 (0.00 - 0.00) |
| United Republic of Tanzania | 704996 (502439,950512) | 13512.46 (9575.55,18277.37) |  | 1822968 (1296369,2460873) | 13512.46 (9575.55,18277.37) |  | 0.00 (-0.00 - 0.00) |
| United States of America | 9689056 (6927303,13100338) | 13781.07 (9851.67,18617.13) |  | 9376592 (6630620,12826467) | 11819.07 (8364.03,16159.79) |  | -0.48 (-0.64 - -0.33) |
| United States Virgin Islands | 6071 (4455,7860) | 20768.52 (15234.9,26934.84) |  | 3784 (2780,4889) | 20768.52 (15234.9,26934.84) |  | 0.00 (0.00 - 0.00) |
| ASPR, age-standardized prevalence rate; UI, uncertainty interval; AAPC, average annual percentage change; CI, confidence interval. | | | | | | | |

| Table S6. The YLDs and age-standardized YLD rates of gastroesophageal reflux disease in women of childbearing age in 1990 and 2021, and estimated average annual percentage changes from 1990 to 2021 | | | | | | | |
| --- | --- | --- | --- | --- | --- | --- | --- |
|  | 1990 | |  | 2021 | |  | 1990 to 2021 |
| Location | Number of cases | ASYR per 100,000 people (95% UI) |  | Number of cases | ASYR per 100,000 people (95% UI) |  | AAPC (95% CI) |
| American Samoa | 6 (3,11) | 50.89 (23.9,97.13) |  | 6 (3,11) | 50.63 (23.53,96.73) |  | -0.01 (-0.03 - -0.00) |
| Antigua and Barbuda | 26 (12,47) | 161.8 (79.24,296.61) |  | 41 (20,75) | 161.78 (78.9,295.61) |  | 0.00 (-0.00 - 0.00) |
| Arab Republic of Egypt | 15457 (7333,28854) | 125.12 (59.54,234.18) |  | 31885 (14988,59866) | 125.25 (59,235.41) |  | 0.00 (-0.01 - 0.01) |
| Argentine Republic | 10250 (4869,19180) | 129.38 (61.49,242.13) |  | 15775 (7523,29535) | 129.23 (61.62,241.85) |  | -0.01 (-0.04 - 0.02) |
| Australia | 3821 (1784,7258) | 83.93 (39.18,159.35) |  | 5392 (2545,10245) | 84.02 (39.53,159.47) |  | -0.00 (-0.04 - 0.03) |
| Barbados | 110 (54,203) | 161.99 (79.17,297.89) |  | 121 (59,223) | 161.75 (79.08,297.52) |  | -0.00 (-0.01 - 0.00) |
| Belize | 60 (29,111) | 161.89 (78.97,297.24) |  | 188 (92,345) | 161.58 (78.86,296.36) |  | -0.01 (-0.02 - 0.00) |
| Bermuda | 29 (14,54) | 162.25 (79.43,300.26) |  | 24 (12,44) | 162.28 (78.85,297.82) |  | 0.00 (-0.00 - 0.00) |
| Bolivarian Republic of Venezuela | 7377 (3580,13641) | 162.07 (79,299.21) |  | 11520 (5606,21197) | 161.83 (78.52,298.37) |  | -0.00 (-0.01 - -0.00) |
| Bosnia and Herzegovina | 1065 (498,2015) | 91.65 (42.9,173.53) |  | 730 (345,1377) | 91.51 (43.07,171.79) |  | -0.00 (-0.01 - 0.00) |
| Brunei Darussalam | 35 (16,67) | 54.99 (25.56,103.78) |  | 72 (34,137) | 55.02 (25.68,104.99) |  | 0.00 (-0.00 - 0.00) |
| Burkina Faso | 1981 (925,3701) | 104.49 (48.98,195.97) |  | 5187 (2443,9655) | 105.04 (49.66,196.58) |  | 0.02 (0.00 - 0.03) |
| Canada | 5219 (2418,9911) | 67.4 (31.24,127.86) |  | 6021 (2825,11553) | 67.36 (31.53,128.8) |  | 0.00 (-0.02 - 0.02) |
| Central African Republic | 605 (285,1136) | 103.95 (49.14,196.16) |  | 1316 (617,2460) | 104.21 (49.01,195.45) |  | 0.01 (0.00 - 0.02) |
| Commonwealth of Dominica | 25 (12,46) | 161.77 (78.71,294.99) |  | 27 (13,49) | 161.6 (78.48,296.32) |  | -0.00 (-0.01 - 0.00) |
| Commonwealth of the Bahamas | 112 (54,207) | 161.71 (78.58,297.88) |  | 177 (86,327) | 161.52 (78.04,297.88) |  | -0.00 (-0.01 - 0.00) |
| Cook Islands | 2 (1,4) | 50.88 (24.06,96.47) |  | 2 (1,4) | 50.81 (24,97) |  | -0.00 (-0.02 - 0.01) |
| Czech Republic | 2458 (1156,4662) | 91.55 (43.04,173.35) |  | 2377 (1123,4468) | 91.61 (42.99,170.89) |  | 0.00 (-0.01 - 0.01) |
| Democratic People's Republic of Korea | 2519 (1174,4821) | 45.57 (21.25,87.51) |  | 3099 (1438,5897) | 45.54 (21.07,86.46) |  | -0.00 (-0.00 - 0.00) |
| Democratic Republic of Sao Tome and Principe | 23 (11,44) | 105.24 (49.42,198.29) |  | 55 (26,104) | 105.27 (49.33,198.95) |  | 0.00 (0.00 - 0.00) |
| Democratic Republic of the Congo | 7912 (3720,14695) | 103.27 (48.64,192.79) |  | 20101 (9320,37725) | 104.32 (48.48,196.57) |  | 0.03 (0.02 - 0.04) |
| Democratic Republic of Timor-Leste | 90 (42,172) | 50.8 (23.99,97.09) |  | 160 (74,305) | 51 (23.76,97.41) |  | 0.01 (0.00 - 0.03) |
| Democratic Socialist Republic of Sri Lanka | 2258 (1066,4332) | 50.75 (24.01,97.43) |  | 2938 (1371,5609) | 50.94 (23.75,97.24) |  | 0.01 (0.01 - 0.02) |
| Dominican Republic | 2786 (1356,5166) | 161.79 (79.12,298.66) |  | 4658 (2266,8583) | 161.53 (78.67,297.38) |  | -0.00 (-0.01 - -0.00) |
| Eastern Republic of Uruguay | 973 (465,1819) | 129.6 (61.89,242.29) |  | 1113 (529,2097) | 129.15 (61.44,243.17) |  | -0.01 (-0.02 - -0.00) |
| Federal Democratic Republic of Ethiopia | 10903 (5181,20416) | 108.32 (51.65,203.21) |  | 26910 (12757,50615) | 108.74 (51.76,204.89) |  | 0.01 (0.01 - 0.02) |
| Federal Democratic Republic of Nepal | 5075 (2405,9545) | 120.6 (57.37,227.57) |  | 10586 (4984,20083) | 121.1 (57.15,229.74) |  | 0.01 (0.00 - 0.02) |
| Federal Republic of Germany | 13755 (6482,26604) | 67.31 (31.69,130.06) |  | 12689 (5958,24188) | 68.72 (32.19,130.35) |  | 0.06 (0.05 - 0.08) |
| Federal Republic of Nigeria | 18834 (8954,35123) | 107.95 (51.51,202.04) |  | 55015 (26174,102866) | 108.46 (51.72,203.35) |  | 0.01 (0.01 - 0.02) |
| Federal Republic of Somalia | 1632 (766,3040) | 104.09 (48.97,194.59) |  | 4504 (2113,8440) | 104.61 (49.27,196.72) |  | 0.02 (0.01 - 0.02) |
| Federated States of Micronesia | 11 (5,20) | 50.82 (23.96,96.46) |  | 13 (6,24) | 50.76 (23.86,96.74) |  | -0.00 (-0.00 - 0.00) |
| Federative Republic of Brazil | 62406 (30160,115532) | 168.59 (81.86,312.14) |  | 102566 (49802,187438) | 167.08 (80.99,305.37) |  | -0.03 (-0.05 - -0.02) |
| French Republic | 9292 (4342,17672) | 62.78 (29.34,119.14) |  | 9397 (4340,17976) | 62.71 (29,119.43) |  | -0.01 (-0.06 - 0.04) |
| Gabonese Republic | 203 (96,376) | 104.03 (49.23,193.99) |  | 477 (223,905) | 104.16 (48.66,198.14) |  | 0.00 (-0.01 - 0.01) |
| Georgia | 1391 (656,2644) | 100.49 (47.39,190.93) |  | 862 (411,1650) | 100.28 (47.62,191.72) |  | -0.00 (-0.01 - 0.00) |
| Grand Duchy of Luxembourg | 76 (36,143) | 73.98 (34.6,138.71) |  | 126 (59,238) | 73.97 (34.41,139.28) |  | 0.00 (0.00 - 0.00) |
| Greenland | 11 (5,21) | 77.13 (36.04,145.11) |  | 10 (5,19) | 77.22 (36.06,145.65) |  | 0.00 (-0.00 - 0.01) |
| Grenada | 29 (14,53) | 161.51 (78.66,296.75) |  | 42 (20,77) | 161.55 (78.41,299.03) |  | -0.00 (-0.00 - 0.00) |
| Guam | 17 (8,33) | 50.95 (24,97.09) |  | 19 (9,36) | 50.87 (24,98.22) |  | -0.01 (-0.02 - 0.01) |
| Hashemite Kingdom of Jordan | 913 (428,1706) | 125.15 (59.19,235.1) |  | 3720 (1738,6946) | 124.89 (58.45,233.52) |  | -0.01 (-0.01 - -0.00) |
| Hellenic Republic | 2598 (1210,4946) | 101 (47.06,192.22) |  | 2416 (1130,4637) | 100.68 (46.93,193.25) |  | -0.01 (-0.03 - 0.01) |
| Hungary | 2903 (1364,5485) | 108.83 (51.11,205.31) |  | 2595 (1222,4884) | 108.83 (51.18,203.69) |  | 0.01 (0.00 - 0.01) |
| Independent State of Papua New Guinea | 452 (212,862) | 50.53 (23.79,96.58) |  | 1295 (602,2450) | 50.74 (23.58,96.07) |  | 0.01 (0.00 - 0.02) |
| Independent State of Samoa | 17 (8,32) | 50.88 (23.77,96.58) |  | 23 (11,45) | 50.82 (23.91,97.49) |  | -0.00 (-0.01 - -0.00) |
| Ireland | 640 (299,1201) | 73.97 (34.53,138.72) |  | 927 (437,1737) | 73.88 (34.73,137.83) |  | -0.00 (-0.00 - -0.00) |
| Islamic Republic of Afghanistan | 2385 (1126,4467) | 124.17 (58.76,232.64) |  | 8041 (3773,15135) | 124.25 (58.55,234.35) |  | 0.00 (-0.00 - 0.01) |
| Islamic Republic of Iran | 13090 (6247,24550) | 115.62 (55.35,217.68) |  | 29090 (13928,54726) | 115.69 (55.4,217.46) |  | 0.00 (-0.02 - 0.03) |
| Islamic Republic of Mauritania | 444 (209,834) | 104.74 (49.42,197.54) |  | 1023 (484,1924) | 105.1 (49.83,198.6) |  | 0.01 (0.00 - 0.01) |
| Islamic Republic of Pakistan | 26469 (12478,49370) | 124.79 (59,233.49) |  | 72026 (34334,134025) | 124.78 (59.62,232.69) |  | -0.00 (-0.00 - 0.00) |
| Jamaica | 885 (430,1633) | 161.99 (79.01,298.1) |  | 1264 (615,2322) | 161.84 (78.8,297.01) |  | -0.00 (-0.00 - 0.00) |
| Japan | 14881 (6985,28429) | 44.3 (20.76,84.12) |  | 12255 (5749,23599) | 44.3 (20.71,84.52) |  | -0.00 (-0.02 - 0.02) |
| Kingdom of Bahrain | 140 (66,261) | 125.14 (59,234.57) |  | 419 (196,782) | 124.95 (58.35,233.17) |  | -0.00 (-0.01 - 0.00) |
| Kingdom of Belgium | 2027 (967,3892) | 80.34 (38.31,154.1) |  | 2163 (1030,4167) | 81.6 (38.78,156.95) |  | 0.05 (0.03 - 0.07) |
| Kingdom of Bhutan | 151 (71,286) | 121.33 (57.41,230.71) |  | 250 (118,474) | 121.6 (57.36,231.01) |  | 0.01 (0.01 - 0.01) |
| Kingdom of Cambodia | 1184 (559,2235) | 50.66 (24.04,95.8) |  | 2291 (1080,4385) | 50.93 (24.04,97.4) |  | 0.02 (0.01 - 0.02) |
| Kingdom of Denmark | 1272 (598,2457) | 93.34 (43.83,179.94) |  | 1240 (582,2347) | 93.43 (43.79,176.56) |  | 0.00 (-0.01 - 0.01) |
| Kingdom of Eswatini | 180 (83,339) | 105.17 (48.86,199.27) |  | 310 (145,575) | 103.66 (48.65,192.89) |  | -0.05 (-0.06 - -0.04) |
| Kingdom of Lesotho | 369 (173,692) | 104.79 (49.17,197.01) |  | 487 (230,914) | 103.38 (48.85,194.39) |  | -0.04 (-0.06 - -0.03) |
| Kingdom of Morocco | 7324 (3460,13758) | 124.96 (59.31,235.11) |  | 12233 (5764,22963) | 124.74 (58.75,234.01) |  | -0.01 (-0.01 - -0.00) |
| Kingdom of Norway | 415 (196,793) | 38.15 (18.05,72.86) |  | 493 (229,940) | 38.17 (17.73,72.52) |  | 0.00 (0.00 - 0.01) |
| Kingdom of Saudi Arabia | 3703 (1741,6952) | 124.99 (59.02,235.8) |  | 13481 (6371,25472) | 125.06 (59.07,236.33) |  | -0.00 (-0.01 - 0.01) |
| Kingdom of Spain | 6476 (3009,12289) | 67.58 (31.4,128.21) |  | 7453 (3509,14168) | 67.38 (31.49,126.81) |  | -0.01 (-0.04 - 0.01) |
| Kingdom of Sweden | 1357 (648,2571) | 62.68 (29.84,118.28) |  | 1556 (733,2991) | 66 (31.06,126.39) |  | 0.17 (0.15 - 0.19) |
| Kingdom of Thailand | 7845 (3657,14937) | 50.95 (23.81,97.17) |  | 8928 (4199,17123) | 51 (23.89,97.65) |  | 0.01 (0.01 - 0.01) |
| Kingdom of the Netherlands | 2205 (1046,4265) | 54 (25.62,104.27) |  | 2083 (986,4015) | 53.89 (25.5,103.53) |  | -0.01 (-0.02 - 0.01) |
| Kingdom of Tonga | 10 (5,20) | 50.88 (24.1,97.59) |  | 12 (6,23) | 50.81 (23.87,96.59) |  | -0.01 (-0.01 - 0.00) |
| Kyrgyz Republic | 969 (453,1849) | 100.15 (47.08,191.52) |  | 1732 (822,3293) | 100.21 (47.59,190.46) |  | 0.00 (-0.01 - 0.01) |
| Lao People's Democratic Republic | 453 (212,867) | 50.78 (23.91,97.49) |  | 991 (464,1878) | 50.94 (23.9,96.7) |  | 0.01 (-0.00 - 0.02) |
| Lebanese Republic | 903 (420,1700) | 124.95 (58.25,235.86) |  | 1957 (924,3670) | 124.82 (58.82,233.78) |  | -0.00 (-0.01 - 0.01) |
| Malaysia | 2173 (1010,4137) | 50.94 (23.78,97.27) |  | 4309 (2004,8295) | 50.9 (23.69,97.97) |  | 0.00 (-0.00 - 0.00) |
| Mongolia | 454 (212,866) | 100.08 (46.84,191.12) |  | 890 (419,1696) | 100.16 (47.1,190.85) |  | 0.00 (-0.01 - 0.02) |
| Montenegro | 143 (67,269) | 91.7 (42.84,172.9) |  | 141 (67,265) | 91.51 (43.14,171.62) |  | -0.01 (-0.02 - -0.00) |
| New Zealand | 829 (395,1583) | 91.19 (43.51,174.28) |  | 1147 (552,2190) | 91.45 (43.94,174.4) |  | 0.01 (0.00 - 0.01) |
| North Macedonia | 468 (219,880) | 91.59 (42.94,172.27) |  | 532 (250,990) | 91.47 (42.95,169.64) |  | -0.00 (-0.01 - 0.01) |
| Northern Mariana Islands | 7 (3,13) | 50.96 (23.95,97.51) |  | 6 (3,12) | 50.94 (24.06,97.37) |  | 0.00 (-0.00 - 0.01) |
| Palestine | 491 (229,918) | 125.11 (58.48,235.11) |  | 1538 (719,2878) | 125.06 (58.67,235.02) |  | -0.00 (-0.01 - 0.01) |
| People's Democratic Republic of Algeria | 6550 (3096,12190) | 125.21 (59.45,233.96) |  | 14619 (6883,27584) | 125.21 (58.88,236.17) |  | 0.00 (-0.00 - 0.00) |
| People's Republic of Bangladesh | 27705 (13072,51905) | 127.65 (60.6,239.77) |  | 57948 (27680,109540) | 127.99 (61.18,242.11) |  | -0.00 (-0.08 - 0.08) |
| People's Republic of China | 136383 (63581,259237) | 44.46 (20.78,84.78) |  | 152951 (71764,292842) | 44.18 (20.66,84.22) |  | -0.00 (-0.04 - 0.03) |
| Plurinational State of Bolivia | 2298 (1120,4251) | 161.55 (79.01,298.15) |  | 4986 (2433,9181) | 161.63 (78.97,297.36) |  | -0.00 (-0.01 - 0.00) |
| Portuguese Republic | 2205 (1036,4197) | 86.76 (40.77,165.08) |  | 2223 (1040,4209) | 86.67 (40.42,163.52) |  | -0.00 (-0.01 - 0.01) |
| Principality of Andorra | 11 (5,21) | 74.1 (34.59,139.1) |  | 17 (8,32) | 73.92 (34.63,139.91) |  | -0.01 (-0.02 - 0.00) |
| Principality of Monaco | 6 (3,11) | 74.13 (34.89,138.85) |  | 6 (3,11) | 73.86 (34.78,138.65) |  | -0.01 (-0.01 - -0.01) |
| Puerto Rico | 1546 (751,2835) | 162.07 (78.8,297.31) |  | 1271 (621,2330) | 162.01 (78.99,297.69) |  | -0.00 (-0.01 - 0.00) |
| Republic of Albania | 445 (208,837) | 57.25 (26.8,108.12) |  | 365 (172,691) | 57.18 (26.88,108.24) |  | -0.00 (-0.02 - 0.02) |
| Republic of Angola | 2170 (1017,4043) | 104.3 (49.05,195.19) |  | 7288 (3435,13602) | 104.38 (49.33,195.66) |  | 0.00 (-0.01 - 0.01) |
| Republic of Armenia | 852 (398,1619) | 100.36 (46.97,191) |  | 808 (382,1538) | 100.39 (47.4,190.9) |  | 0.00 (-0.00 - 0.01) |
| Republic of Austria | 1745 (797,3367) | 85.47 (39.01,164.88) |  | 1836 (840,3562) | 85.49 (39.04,165.27) |  | 0.00 (-0.02 - 0.02) |
| Republic of Azerbaijan | 1758 (824,3342) | 100.33 (47.32,191.24) |  | 2928 (1381,5603) | 100.29 (47.23,191.53) |  | 0.00 (-0.00 - 0.01) |
| Republic of Belarus | 2824 (1322,5341) | 108.78 (50.94,205.87) |  | 2569 (1213,4882) | 108.64 (51.2,205.91) |  | -0.00 (-0.01 - 0.00) |
| Republic of Benin | 1038 (488,1932) | 104.21 (49.08,195.03) |  | 3060 (1421,5704) | 104.74 (48.78,196.05) |  | 0.02 (0.01 - 0.03) |
| Republic of Botswana | 297 (138,558) | 104.83 (48.71,197.92) |  | 712 (335,1335) | 104.1 (49.1,195.36) |  | -0.02 (-0.02 - -0.02) |
| Republic of Bulgaria | 1984 (930,3727) | 91.57 (42.94,171.51) |  | 1465 (695,2793) | 91.45 (43.16,173.39) |  | -0.01 (-0.01 - 0.00) |
| Republic of Burundi | 1197 (559,2252) | 104.75 (49.09,198.48) |  | 2940 (1371,5498) | 104.89 (49.1,197.63) |  | 0.01 (0.01 - 0.02) |
| Republic of Cabo Verde | 73 (34,135) | 105.2 (49.81,196.27) |  | 157 (73,295) | 105.11 (49.2,198.74) |  | -0.00 (-0.01 - 0.00) |
| Republic of Cameroon | 2226 (1041,4121) | 104.45 (49.07,194.51) |  | 7496 (3497,13897) | 104.74 (49.07,194.9) |  | 0.01 (0.00 - 0.02) |
| Republic of Chad | 1251 (587,2354) | 104.45 (49.24,197.62) |  | 3529 (1647,6553) | 104.62 (49.07,195.43) |  | 0.01 (0.00 - 0.01) |
| Republic of Chile | 4527 (2153,8511) | 129.15 (61.42,243.26) |  | 6363 (3017,11984) | 128.97 (61.01,242.7) |  | -0.01 (-0.01 - 0.00) |
| Republic of Colombia | 13253 (6448,24508) | 162.28 (79.29,298.93) |  | 21582 (10469,39879) | 162.15 (78.65,299.56) |  | -0.00 (-0.01 - 0.00) |
| Republic of Costa Rica | 1201 (580,2199) | 162.12 (78.64,296.23) |  | 2168 (1057,3972) | 161.86 (78.91,296.41) |  | -0.00 (-0.01 - 0.00) |
| Republic of Croatia | 1148 (540,2191) | 91.54 (43,174.49) |  | 899 (426,1699) | 91.69 (43.27,172.33) |  | 0.00 (-0.00 - 0.01) |
| Republic of Cuba | 4771 (2328,8825) | 161.6 (79.08,298.61) |  | 4287 (2101,7887) | 161.94 (78.98,298.34) |  | 0.01 (0.00 - 0.01) |
| Republic of Cyprus | 149 (70,280) | 74.14 (34.77,139.15) |  | 296 (141,563) | 74.07 (35.13,140.12) |  | -0.00 (-0.00 - -0.00) |
| Republic of C么te d'Ivoire | 2523 (1180,4712) | 103.89 (48.85,195.17) |  | 6523 (3044,12293) | 104.77 (49.03,198.36) |  | 0.02 (0.01 - 0.03) |
| Republic of Djibouti | 91 (43,169) | 105.06 (49.66,196.59) |  | 341 (160,637) | 105.16 (49.42,196.9) |  | 0.00 (-0.01 - 0.01) |
| Republic of Ecuador | 3775 (1816,6951) | 162.17 (78.41,297.65) |  | 7610 (3689,14064) | 161.95 (78.57,299.18) |  | -0.01 (-0.01 - -0.00) |
| Republic of El Salvador | 1926 (933,3548) | 161.95 (78.8,297.97) |  | 2876 (1399,5340) | 162.2 (78.96,301.1) |  | 0.01 (0.01 - 0.01) |
| Republic of Equatorial Guinea | 93 (44,174) | 103.7 (48.79,194.3) |  | 352 (164,659) | 104.3 (48.83,195.89) |  | 0.02 (0.01 - 0.03) |
| Republic of Estonia | 433 (203,816) | 108.7 (51.05,204.78) |  | 332 (157,632) | 108.65 (51.15,205.5) |  | -0.00 (-0.01 - 0.01) |
| Republic of Fiji | 95 (44,181) | 50.78 (23.64,96.94) |  | 116 (54,221) | 50.76 (23.42,96.5) |  | -0.00 (-0.01 - 0.01) |
| Republic of Finland | 1353 (629,2549) | 99.82 (46.33,187.93) |  | 1209 (574,2297) | 100.13 (47.4,189.92) |  | 0.01 (-0.01 - 0.03) |
| Republic of Ghana | 3346 (1568,6236) | 104.44 (49.24,195.82) |  | 9132 (4250,17140) | 104.79 (48.89,197.42) |  | 0.01 (0.00 - 0.02) |
| Republic of Guatemala | 2681 (1302,4939) | 160.97 (78.44,295.61) |  | 6757 (3265,12393) | 161.18 (78.15,295.3) |  | 0.00 (-0.00 - 0.01) |
| Republic of Guinea | 1340 (628,2496) | 104.48 (48.99,195.4) |  | 3172 (1472,5946) | 104.72 (48.79,197.37) |  | 0.00 (-0.00 - 0.01) |
| Republic of Guinea-Bissau | 219 (103,409) | 104.35 (49.13,196.28) |  | 508 (236,957) | 104.63 (48.88,197.98) |  | 0.01 (0.01 - 0.01) |
| Republic of Guyana | 302 (147,556) | 160.85 (78.52,295.36) |  | 321 (156,589) | 160.47 (78.29,294.37) |  | -0.01 (-0.02 - 0.00) |
| Republic of Haiti | 2299 (1115,4240) | 160.13 (77.95,294.42) |  | 5581 (2725,10262) | 160.25 (78.37,294.28) |  | 0.00 (-0.01 - 0.01) |
| Republic of Honduras | 1541 (749,2851) | 162 (79.17,299.22) |  | 4395 (2131,8145) | 161.64 (78.6,298.97) |  | -0.01 (-0.01 - -0.00) |
| Republic of Iceland | 40 (19,75) | 61.29 (28.79,116.83) |  | 52 (24,98) | 62.3 (29.21,117.52) |  | 0.05 (0.04 - 0.07) |
| Republic of India | 223218 (106928,421854) | 117.97 (56.63,223.45) |  | 441264 (212534,834357) | 118.27 (57.01,223.75) |  | 0.01 (0.00 - 0.01) |
| Republic of Indonesia | 23744 (11139,45153) | 53.11 (24.97,101.17) |  | 41068 (19357,77459) | 53.15 (25.03,100.22) |  | 0.00 (-0.00 - 0.01) |
| Republic of Iraq | 4564 (2136,8599) | 124.91 (58.81,236.62) |  | 12685 (5991,23740) | 124.65 (58.91,233.53) |  | -0.00 (-0.01 - 0.00) |
| Republic of Italy | 13642 (6592,25722) | 93.53 (45.17,176.25) |  | 12646 (6125,23918) | 93.62 (45.1,176.6) |  | 0.00 (-0.01 - 0.02) |
| Republic of Kazakhstan | 4025 (1913,7649) | 100.1 (47.61,190.2) |  | 5013 (2374,9571) | 100.12 (47.34,190.96) |  | -0.00 (-0.01 - 0.01) |
| Republic of Kenya | 4766 (2262,8920) | 108.72 (51.76,204.27) |  | 13073 (6211,24473) | 108.66 (51.75,203.93) |  | -0.00 (-0.01 - 0.00) |
| Republic of Kiribati | 9 (4,17) | 50.64 (23.82,96.4) |  | 16 (7,30) | 50.69 (23.69,96.69) |  | 0.00 (-0.00 - 0.01) |
| Republic of Korea | 7710 (3598,14608) | 62.92 (29.45,119.39) |  | 8075 (3751,15512) | 62.9 (29.04,120.22) |  | 0.01 (-0.09 - 0.11) |
| Republic of Latvia | 737 (349,1386) | 108.74 (51.46,204.26) |  | 469 (223,891) | 108.8 (51.66,205.8) |  | 0.00 (-0.00 - 0.01) |
| Republic of Liberia | 516 (243,965) | 103.08 (48.54,193.73) |  | 1342 (627,2533) | 103.51 (48.47,195.83) |  | 0.01 (0.01 - 0.01) |
| Republic of Lithuania | 1059 (499,1983) | 112.05 (52.78,209.63) |  | 698 (325,1313) | 111.98 (51.98,210.2) |  | -0.00 (-0.01 - 0.01) |
| Republic of Madagascar | 2497 (1159,4669) | 104.66 (48.79,196.93) |  | 6898 (3210,12997) | 104.97 (49.01,198.77) |  | 0.01 (0.00 - 0.02) |
| Republic of Malawi | 2072 (969,3880) | 103.85 (48.78,195.32) |  | 4599 (2145,8649) | 104.69 (49.07,197.93) |  | 0.03 (0.01 - 0.04) |
| Republic of Maldives | 21 (10,41) | 50.65 (23.81,97.42) |  | 62 (29,118) | 50.8 (23.82,97.53) |  | 0.01 (0.01 - 0.01) |
| Republic of Mali | 1836 (850,3445) | 104.4 (48.54,196.76) |  | 4997 (2353,9438) | 104.53 (49.41,197.89) |  | 0.01 (-0.01 - 0.02) |
| Republic of Malta | 74 (35,139) | 74.08 (35.12,138.48) |  | 77 (36,144) | 73.97 (34.65,138.35) |  | -0.00 (-0.01 - -0.00) |
| Republic of Mauritius | 149 (69,280) | 50.78 (23.73,95.78) |  | 166 (79,320) | 50.74 (23.92,97.32) |  | -0.00 (-0.01 - 0.01) |
| Republic of Moldova | 1251 (591,2347) | 108.77 (51.41,203.95) |  | 1072 (513,2046) | 108.81 (51.82,206.78) |  | 0.00 (-0.01 - 0.01) |
| Republic of Mozambique | 2994 (1399,5604) | 104.04 (48.74,195.67) |  | 6924 (3264,12913) | 103.41 (48.99,193.57) |  | -0.02 (-0.03 - 0.00) |
| Republic of Namibia | 313 (146,587) | 104.76 (49.08,197.4) |  | 662 (310,1246) | 104.56 (49.07,197.35) |  | -0.01 (-0.01 - -0.00) |
| Republic of Nauru | 1 (1,2) | 50.8 (23.82,97.14) |  | 1 (1,3) | 50.73 (23.87,97.16) |  | -0.00 (-0.00 - -0.00) |
| Republic of Nicaragua | 1304 (625,2419) | 162.02 (78.18,299.34) |  | 2912 (1415,5369) | 161.88 (78.77,298.25) |  | -0.00 (-0.01 - 0.01) |
| Republic of Niue | 0 (0,0) | 50.88 (23.94,97.25) |  | 0 (0,0) | 50.83 (23.88,97.54) |  | -0.00 (-0.00 - 0.00) |
| Republic of Palau | 2 (1,4) | 50.8 (23.79,97.09) |  | 2 (1,4) | 50.8 (23.86,96.26) |  | -0.00 (-0.01 - 0.00) |
| Republic of Panama | 930 (453,1718) | 162.1 (79.17,298.72) |  | 1732 (850,3186) | 161.94 (79.42,297.97) |  | -0.00 (-0.01 - 0.00) |
| Republic of Paraguay | 1466 (699,2705) | 166.99 (80.06,307.95) |  | 3121 (1500,5769) | 166.52 (80.24,307.78) |  | -0.00 (-0.01 - 0.00) |
| Republic of Peru | 8121 (3931,15031) | 161.86 (78.78,298.71) |  | 15869 (7708,29185) | 161.97 (78.67,297.85) |  | -0.00 (-0.01 - 0.00) |
| Republic of Poland | 12287 (5870,22950) | 125.36 (59.89,234.23) |  | 12485 (5987,23391) | 125.67 (60.04,235.18) |  | 0.01 (0.00 - 0.01) |
| Republic of Rwanda | 1513 (705,2859) | 104.8 (48.97,198.71) |  | 3441 (1605,6442) | 104.91 (49.09,197.04) |  | 0.00 (-0.01 - 0.02) |
| Republic of San Marino | 5 (2,9) | 74.04 (34.8,139.66) |  | 6 (3,11) | 73.97 (34.71,138.38) |  | -0.01 (-0.01 - 0.00) |
| Republic of Senegal | 1588 (745,2972) | 104.32 (49.14,196.29) |  | 3755 (1771,7010) | 104.64 (49.47,195.92) |  | 0.01 (0.00 - 0.02) |
| Republic of Serbia | 2198 (1037,4109) | 91.63 (43.19,171.24) |  | 2005 (944,3797) | 91.57 (42.99,172.82) |  | -0.00 (-0.01 - 0.00) |
| Republic of Seychelles | 9 (4,16) | 50.98 (24.29,98.25) |  | 13 (6,25) | 50.9 (23.7,97.47) |  | -0.00 (-0.01 - 0.01) |
| Republic of Sierra Leone | 963 (450,1799) | 104.57 (49.07,196.31) |  | 2155 (1004,4045) | 104.83 (49.04,197.46) |  | 0.01 (0.00 - 0.01) |
| Republic of Singapore | 606 (285,1152) | 64.03 (30.08,121.72) |  | 1079 (506,2054) | 64.64 (30.37,122.36) |  | 0.03 (0.02 - 0.05) |
| Republic of Slovenia | 470 (222,885) | 91.48 (43.11,172.07) |  | 431 (203,819) | 91.57 (42.85,173.04) |  | 0.00 (-0.00 - 0.01) |
| Republic of South Africa | 9528 (4508,17777) | 108.26 (51.35,202.31) |  | 16992 (8105,31909) | 107.27 (51.17,201.38) |  | -0.03 (-0.03 - -0.02) |
| Republic of South Sudan | 1179 (557,2220) | 104.31 (49.31,197.32) |  | 2194 (1023,4126) | 104.62 (49.01,197) |  | 0.01 (-0.00 - 0.02) |
| Republic of Sudan | 5348 (2509,9976) | 124.7 (58.79,233.17) |  | 13345 (6228,25005) | 124.8 (58.41,234.38) |  | 0.00 (-0.01 - 0.01) |
| Republic of Suriname | 147 (72,271) | 161.53 (79.27,297.08) |  | 237 (116,435) | 160.87 (78.56,295.41) |  | -0.01 (-0.02 - -0.00) |
| Republic of Tajikistan | 1062 (497,2022) | 100.2 (47.28,191.3) |  | 2497 (1169,4716) | 100.18 (46.98,189.5) |  | -0.00 (-0.01 - 0.01) |
| Republic of the Congo | 515 (240,976) | 104.15 (48.8,198.44) |  | 1435 (668,2713) | 104.25 (48.65,197.33) |  | 0.00 (-0.01 - 0.02) |
| Republic of the Gambia | 210 (99,390) | 104.57 (49.34,195.36) |  | 577 (269,1077) | 104.32 (48.89,195.41) |  | 0.00 (-0.01 - 0.01) |
| Republic of the Marshall Islands | 4 (2,9) | 50.79 (23.9,97.96) |  | 7 (3,14) | 50.66 (23.68,96.9) |  | -0.01 (-0.01 - -0.00) |
| Republic of the Niger | 1618 (752,3039) | 104.45 (48.62,197.07) |  | 4756 (2213,8930) | 105.02 (49.21,197.94) |  | 0.02 (0.00 - 0.03) |
| Republic of the Philippines | 7550 (3542,14207) | 52.91 (24.88,99.73) |  | 15132 (7135,28641) | 52.97 (24.98,100.32) |  | 0.00 (-0.00 - 0.01) |
| Republic of the Union of Myanmar | 4924 (2298,9303) | 50.76 (23.8,96.04) |  | 7668 (3594,14658) | 50.96 (23.89,97.42) |  | 0.01 (0.01 - 0.02) |
| Republic of Trinidad and Tobago | 483 (234,885) | 161.52 (78.52,295.4) |  | 581 (286,1066) | 161.45 (79.34,296.32) |  | 0.00 (0.00 - 0.00) |
| Republic of Tunisia | 2385 (1111,4461) | 125.42 (58.52,235.27) |  | 4051 (1904,7672) | 125.06 (58.62,236.39) |  | -0.01 (-0.01 - -0.00) |
| Republic of Turkey | 18658 (8781,34856) | 139.72 (66.04,261.65) |  | 34069 (16864,61539) | 152.08 (75.11,274.46) |  | 0.27 (0.24 - 0.30) |
| Republic of Uganda | 3436 (1611,6430) | 104.07 (49.02,196.08) |  | 9586 (4497,18089) | 104.91 (49.48,199.33) |  | 0.02 (0.01 - 0.03) |
| Republic of Uzbekistan | 4400 (2075,8294) | 99.9 (47.23,188.59) |  | 9199 (4340,17593) | 100.04 (47.16,191.26) |  | 0.01 (0.00 - 0.01) |
| Republic of Vanuatu | 17 (8,31) | 50.83 (23.84,97.13) |  | 38 (18,73) | 50.8 (24.14,96.8) |  | -0.00 (-0.01 - 0.00) |
| Republic of Yemen | 3118 (1467,5826) | 123.69 (58.44,232.23) |  | 9757 (4614,18417) | 123.85 (58.78,234.64) |  | 0.01 (0.00 - 0.01) |
| Republic of Zambia | 1629 (769,3035) | 104.46 (49.45,195.45) |  | 4609 (2147,8563) | 104.33 (48.78,194.56) |  | -0.00 (-0.01 - 0.01) |
| Republic of Zimbabwe | 2177 (1010,4062) | 104.72 (48.77,196.7) |  | 3940 (1851,7422) | 104.44 (49.28,197.45) |  | -0.01 (-0.01 - -0.00) |
| Romania | 5150 (2389,9716) | 91.45 (42.44,172.38) |  | 4097 (1925,7764) | 91.62 (42.8,172.82) |  | 0.01 (-0.00 - 0.02) |
| Russian Federation | 42272 (19911,79577) | 109.84 (51.77,206.79) |  | 41895 (19750,78904) | 110.34 (52.07,207.56) |  | 0.02 (-0.04 - 0.07) |
| Saint Kitts and Nevis | 15 (7,27) | 161.36 (78.93,296.18) |  | 26 (13,48) | 161.79 (78.45,297.13) |  | 0.01 (0.00 - 0.01) |
| Saint Lucia | 50 (24,93) | 161.34 (78.28,297.96) |  | 77 (38,142) | 161.33 (78.48,296.07) |  | -0.00 (-0.01 - 0.00) |
| Saint Vincent and the Grenadines | 38 (19,71) | 161.79 (79.14,297.37) |  | 46 (22,84) | 161.5 (77.84,296.48) |  | -0.00 (-0.00 - 0.00) |
| Slovak Republic | 1243 (585,2353) | 91.55 (43.07,173.06) |  | 1296 (609,2426) | 91.63 (42.89,170.85) |  | 0.00 (-0.00 - 0.01) |
| Socialist Republic of Viet Nam | 8045 (3758,15393) | 50.99 (23.9,97.83) |  | 13738 (6528,26116) | 51.1 (24.25,97.11) |  | 0.01 (0.01 - 0.01) |
| Solomon Islands | 33 (16,63) | 50.7 (24,95.97) |  | 84 (39,160) | 50.8 (23.68,97.48) |  | 0.00 (-0.00 - 0.01) |
| State of Eritrea | 743 (347,1388) | 104.68 (49.07,196.37) |  | 1635 (768,3069) | 105.15 (49.48,197.99) |  | 0.02 (0.01 - 0.02) |
| State of Israel | 917 (428,1737) | 76.84 (35.92,145.74) |  | 1734 (807,3310) | 76.73 (35.7,146.22) |  | -0.01 (-0.02 - 0.01) |
| State of Kuwait | 506 (238,945) | 125.33 (59.39,235.53) |  | 2036 (972,3838) | 125.26 (59.44,235.5) |  | -0.00 (-0.00 - 0.00) |
| State of Libya | 992 (462,1862) | 125.15 (58.77,236.27) |  | 2559 (1198,4847) | 124.79 (58.33,236.08) |  | -0.01 (-0.02 - -0.00) |
| State of Qatar | 100 (46,187) | 125.12 (58.49,235.18) |  | 762 (358,1428) | 125.07 (58.73,234.97) |  | -0.00 (-0.01 - 0.01) |
| Sultanate of Oman | 396 (186,741) | 125.16 (59,234.97) |  | 1328 (625,2495) | 125.2 (58.93,235.43) |  | 0.00 (-0.01 - 0.01) |
| Swiss Confederation | 832 (393,1594) | 44.64 (21.04,85.23) |  | 957 (447,1807) | 44.58 (20.76,83.81) |  | -0.00 (-0.03 - 0.03) |
| Syrian Arab Republic | 3059 (1428,5685) | 125.08 (58.7,233.55) |  | 4595 (2184,8621) | 124.64 (59.14,233.75) |  | -0.01 (-0.01 - -0.00) |
| Taiwan (Province of China) | 2726 (1265,5200) | 49.79 (23.16,95.3) |  | 3061 (1421,5806) | 50.22 (23.29,95) |  | 0.02 (-0.01 - 0.04) |
| Togolese Republic | 798 (372,1505) | 104.52 (48.92,197.88) |  | 2162 (1004,4058) | 104.83 (48.84,197.03) |  | 0.01 (-0.00 - 0.02) |
| Tokelau | 0 (0,0) | 50.86 (24.13,97.63) |  | 0 (0,0) | 50.82 (24,96.7) |  | -0.00 (-0.01 - -0.00) |
| Turkmenistan | 800 (375,1527) | 100.06 (47.01,191.29) |  | 1255 (590,2407) | 100.23 (47.15,192.19) |  | 0.00 (-0.00 - 0.01) |
| Tuvalu | 1 (1,2) | 50.87 (23.58,97.04) |  | 1 (1,3) | 50.88 (23.82,97.47) |  | -0.00 (-0.01 - 0.01) |
| Ukraine | 14806 (7028,28082) | 112.35 (53.28,212.94) |  | 12839 (6165,24218) | 112.31 (53.75,210.66) |  | 0.00 (-0.01 - 0.01) |
| Union of the Comoros | 98 (45,184) | 105 (48.97,198.8) |  | 198 (93,369) | 105.16 (49.43,196.49) |  | 0.01 (-0.01 - 0.02) |
| United Arab Emirates | 421 (198,788) | 124.99 (59.2,234.38) |  | 2420 (1149,4599) | 124.91 (59.06,235.74) |  | -0.00 (-0.01 - 0.01) |
| United Kingdom of Great Britain and Northern Ireland | 14765 (7043,27320) | 100.51 (47.94,185.94) |  | 16458 (7799,31152) | 100.41 (47.54,189.63) |  | -0.01 (-0.04 - 0.02) |
| United Mexican States | 32158 (15462,59759) | 162.12 (78.36,300.8) |  | 57485 (27863,106548) | 161.83 (78.36,300.06) |  | -0.01 (-0.01 - -0.00) |
| United Republic of Tanzania | 5410 (2531,10053) | 103.48 (48.63,193.54) |  | 14157 (6626,26404) | 104.76 (49.17,196.19) |  | 0.04 (0.03 - 0.05) |
| United States of America | 75341 (36455,141669) | 107.17 (51.82,201.52) |  | 72510 (34714,137541) | 91.46 (43.72,173.28) |  | -0.50 (-0.64 - -0.36) |
| United States Virgin Islands | 47 (23,87) | 162.08 (78.76,298.73) |  | 29 (14,54) | 161.87 (79.29,297.29) |  | -0.00 (-0.01 - 0.01) |
| YLDs, years lived with disability; ASYR, age-standardized YLD rate; UI, uncertainty interval; AAPC, average annual percentage change; CI, confidence interval. | | | | | | | |
